# Synthesis and Comparative Structure–Activity Study of Carbohydrate-Based Phenolic Compounds as α-Glucosidase Inhibitors and Antioxidants

**DOI:** 10.3390/molecules24234340

**Published:** 2019-11-27

**Authors:** Shota Machida, Saki Mukai, Rina Kono, Megumi Funato, Hiroaki Saito, Taketo Uchiyama

**Affiliations:** School of Pharmacy, Nihon University, 7-7-1 Narashinodai, Funabashi, Chiba 274-8555, Japan; phsh18002@g.nihon-u.ac.jp (S.M.); phsa12222@g.nihon-u.ac.jp (S.M.); rinachammm1026ml@icloud.com (R.K.); mgm27.04f@ezweb.ne.jp (M.F.); saito.hiroaki@nihon-u.ac.jp (H.S.)

**Keywords:** 1,5-AG, tellimagrandin I, acertannin, maplexin, ginnalin, polyphenol, α-glucosidase, antioxidant

## Abstract

Twenty-one natural and unnatural phenolic compounds containing a carbohydrate moiety were synthesized and their structure–activity relationship (SAR) was evaluated for α-glucosidase inhibition and antioxidative activity. Varying the position of the galloyl unit on the 1,5-anhydro-d-glucitol (1,5-AG) core resulted in changes in the α-glucosidase inhibitory activity and notably, particularly strong activity was demonstrated when the galloyl unit was present at the C-2 position. Furthermore, increasing the number of the galloyl units significantly affected the α-glucosidase inhibition, and 2,3,4,6-tetra-galloyl-1,5-AG (**54**) and 2,3,4,6-tetra-galloyl-d-glucopyranose (**61**) exhibited excellent activities, which were more than 13-fold higher than the α-glucosidase inhibitory activity of acertannin (**37**). Moreover, a comparative structure-activity study suggested that a hemiacetal hydroxyl functionality in the carbohydrate core and a biaryl bond of the 4,6-*O*-hexahydroxydiphenoyl (HHDP) group, which are components of ellagitannins including tellimagrandin I, are not necessary for the α-glucosidase inhibitory activity. Lastly, the antioxidant activity increased proportionally with the number of galloyl units.

## 1. Introduction

Impaired glucose tolerance increases the risk of vascular events such as atherosclerotic coronary artery disease [1,2]. Particularly, postprandial hyperglycemia is a serious risk factor for cardiovascular diseases and is believed to be the cause of oxidative stress that leads to vascular events [3,4,5,6,7]. Thus, controlling postprandial hyperglycemia is an important target to prevent diabetes as well as diabetic complications. In clinical medicine, α-glucosidase inhibitors such as acarbose, miglitol, and voglibose, belong to the class of antidiabetic drugs used for improving postprandial hyperglycemia [8,9,10,11]. Currently, natural products and their derivatives constitute more than half of the drugs in the clinic [12,13,14,15]. Therefore, finding inspiration in nature to develop more efficient and effective medicines has attracted significant interest.

**Figure 1:**
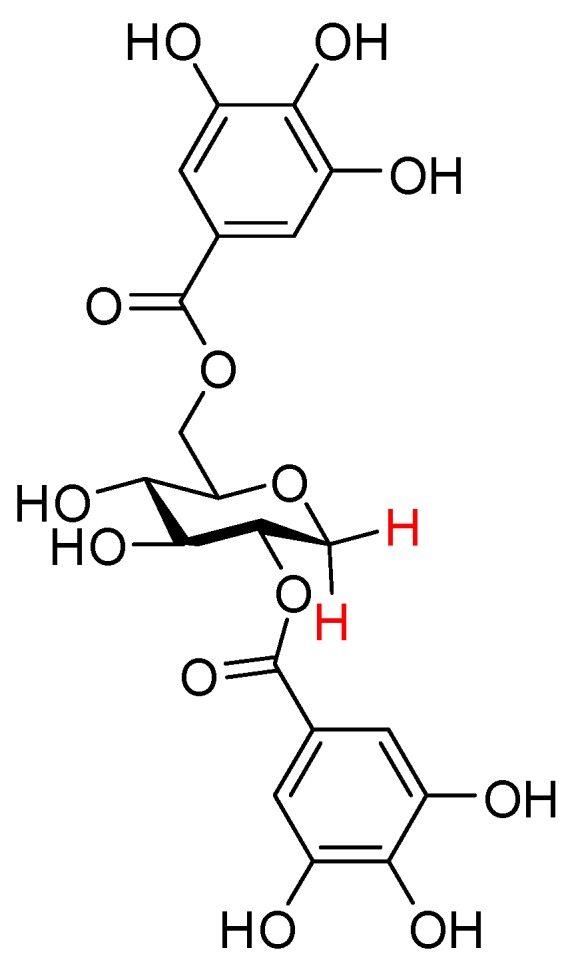
Structure of acertannin.

Trees belonging to the *Acer* species have been used as traditional medicinal plants for many years and are widely known for their sap, which can be concentrated to produce maple syrup [16]. It has been demonstrated that *Acer* extracts display various bioactivities such as anti-cancer [17,18], antioxidant [19,20,21,22], and antihyperglycemic effects [23,24]. A. Honma et al. identified a compound from *Acer saccharum* extracts able to suppress hyperglycemia, namely acertannin, and revealed that its effects are a consequence of potent inhibitory activity toward α-glucosidase [25]. The structural components of acertannin include the characteristic 1,5-anhydro-d-glucitol (1,5-AG) sugar moiety, which lacks the hemiacetal hydroxyl group present in d-glucose, as the carbohydrate core, and two gallic acid functionalities as the phenolic units (Figure 1) [26]. However, only a few plants belonging to the *Acer* genus produce the 1,5-AG core containing polyphenols [27,28]. To date, maplexin A–J and ginnalin A–C have been isolated and characterized. The molecules possess varying numbers and positions of the phenol units esterified with the 1,5-AG core [29,30,31,32]. These polyphenols were shown to exhibit different bioactivities such as α-glucosidase inhibition and antioxidant activity. It is noteworthy that different numbers, positions, and types of the phenol units on the 1,5-AG core display non-identical bioactivities [32,33,34,35,36].

**Figure 2:**
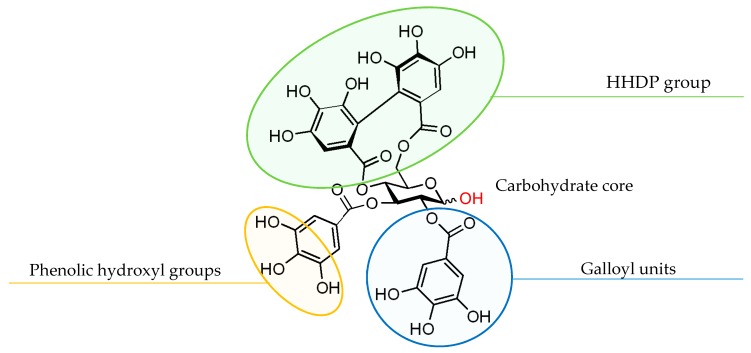
The structural components of tellimagrandin I. HHDP, hexahydroxydiphenoyl.

Tellimagrandin I, which belongs to ellagitannins, has also been demonstrated to be an α-glucosidase inhibitor and to show antioxidant activity [37,38]. The molecule is characterized by the presence of a hexahydroxydiphenoyl (HHDP) group, two galloyl units and the d-glucose core possesses a hemiacetal hydroxyl functionality (Figure 2) [39]. The HHDP group provides structural diversity in polyphenols, and the macro-lactone structure is considered to be the element responsible for the pharmacological activity [40]. Nonetheless, to our knowledge, no reports on the evaluation of the synthesis and/or bioactivity of compounds comprising the HHDP functionality on the 1,5-AG core have been reported so far. Furthermore, the hemiacetal hydroxyl group is a fundamental moiety in the carbohydrate chemistry; however, its effects on the bioactivity remain largely unexplored.

In the present study, we report the synthesis of a series of 21 carbohydrate-based phenolic compounds to investigate the structure–activity relationship (SAR). α-glucosidase inhibition and antioxidant activity were examined by studying the effects of (1) the position and number of galloyl units, (2) the type of phenol units, (3) the existence the 4,6-*O*-HHDP group, and (4) the presence of the hemiacetal hydroxyl group.

## 2. Results

### 2.1. Syntheses of 1,5-AG-Based Polyphenols

#### 2.1.1. Syntheses of Galloylated 1,5-AGs

Recently, A. Kamori et al. reported the synthesis of various natural and unnatural acertannin derivatives and evaluated their SAR against ceramidase and ceramide synthase enzymes [35]. In addition, we have also previously reported a facile method for the preparation of 1,5-anhydroalditol via treatment of per-*O*-TMS-glycopyranosyl iodide with LiBH_4_ [41]. In total, 1,5-AG, which can be easily synthesized from d-glucose on multi-gram scale in three days, possesses four hydroxyl groups; hence, 15 different combinations are possible for mono-, di-, tri-, and tetra-galloylation of 1,5-AG. In the current study, we attempted the synthesis of all of these galloylated compounds starting from 1,5-AG.

**Scheme 1:**
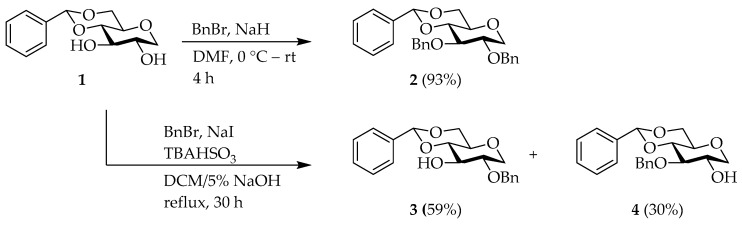
Selective protection of 1,5-AG.

Firstly, 4,6-*O*-benzylidene-1,5-AG (**1**) [42] was protected with benzyl (Bn) group using BnBr and NaH to afford di-benzylated compound **2 [42]**. However, reacting **1** in a 2-phase dichloromethane (DCM)/5% NaOH system with BnBr, NaI, and tetra-*n*-butylammonium hydrogen sulfate provided the 3-OH analog **3** [35] and the 2-OH analog **4** [35] as a mixture of products, which could be separated by column chromatography (C.C.) (Scheme 1) [43].

**Scheme 2:**
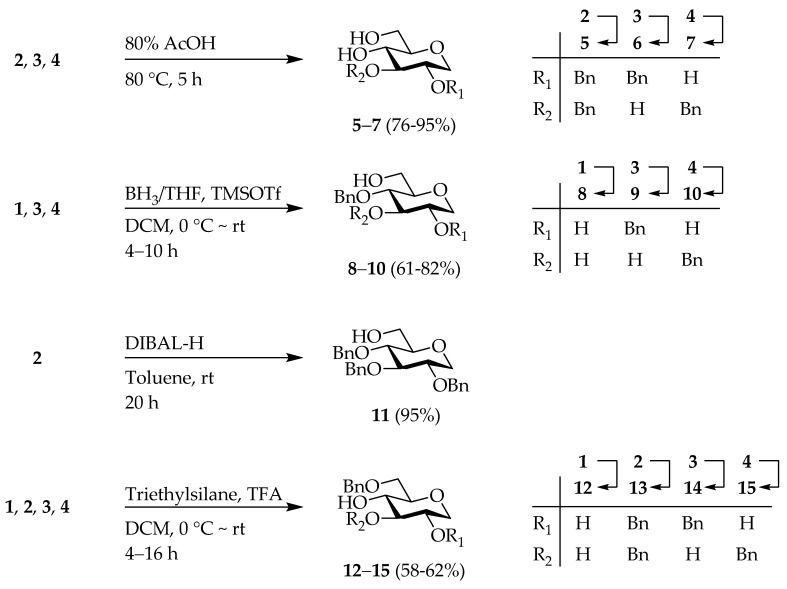
Deprotection of **1**–**4**.

**Scheme 3:**
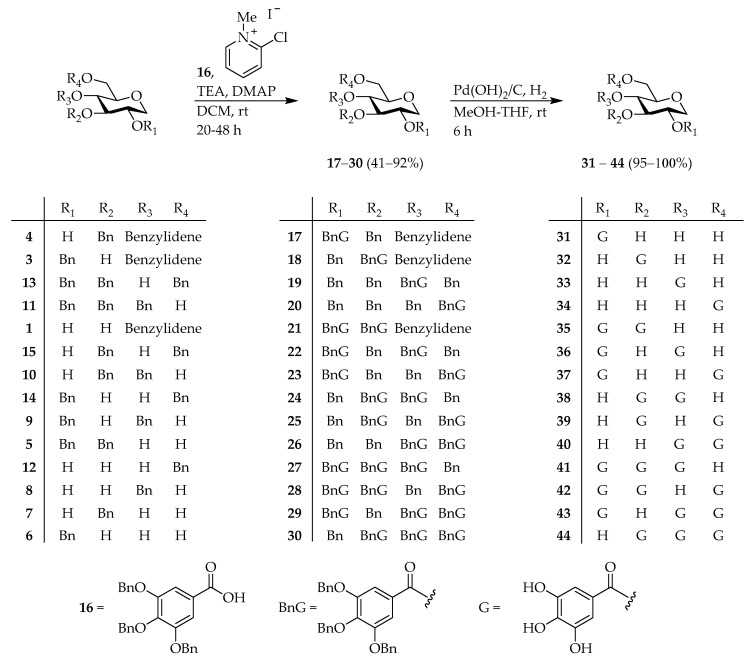
Synthesis of galloylated 1,5-AGs.

Subsequently, the 4,6-*O*-benzylidene groups on the 1,5-AG derivatives **1**–**4** were converted to the corresponding hydroxyl moieties using an acid or reducing reagent (Scheme 2). Compounds **2**–**4** were then deprotected with 80% acetic acid/$H_{2}O$ solution to give the corresponding diol analogs **5**–**7** [35,42]. Reduction of **1**, **3**, and **4** by BH_3_/THF and trimethylsilyl trifluoromethanesulfonate (TMSOTf) [44], and reduction of **2** by diisobutylaluminium hydride (DIBAL-H) [45] resulted in the formation of the 6-OH derivatives **8**–**11** [35,46,47]. Moreover, compounds **1**–**4** were treated with triethylsilane and trifluoroacetic acid (TFA) [48] to afford the 4-OH derivatives **12**–**15** [35,49]. Esterification of these -OH derivatives with Bn-protected gallic acid (**16**) [50] using *N*,*N*-dimethyl-4-aminopyridine (DMAP) and triethylamine (TEA), as well as 2-chloro-1-methylpyridinium iodide as the condensing reagent, provided 1,5-AG-based galloylated derivatives **17**–**30**. Finally, hydrogenolysis using Pd(OH)_2_ as the catalyst in a MeOH/THF solvent mixture gave 14 types of the 1,5-AG core containing polyphenol analogs **31**–**44** (Scheme 3).

#### 2.1.2. Synthesis of Maplexin J Analogs

**Scheme 4:**
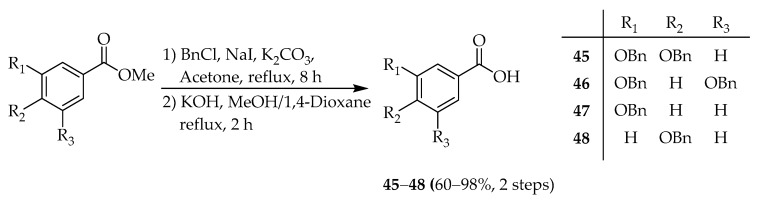
Preparation of the Bn-protected phenol units **45**–**48**.

**Scheme 5:**
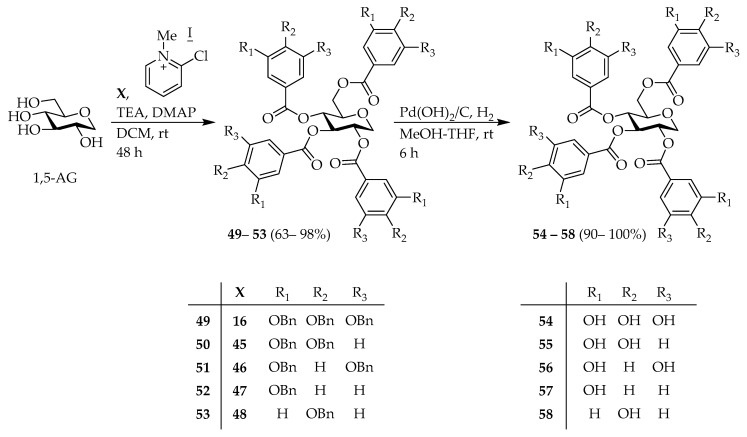
Synthesis of maplexin J analogs **54**–**58**.

**Scheme 6:**
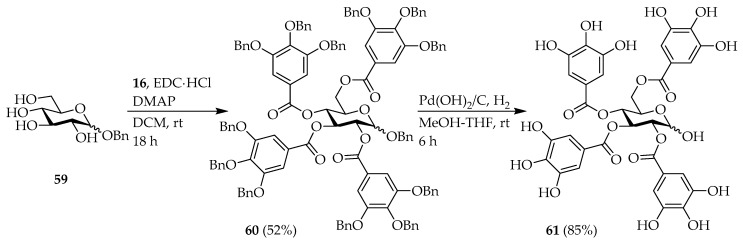
Synthesis of tellimagrandin I analog **61**.

Maplexin J (**54**) [32,50], which is per-galloylated 1,5-AG, could be easily synthesized from 1,5-AG. Therefore, we attempted to synthesize of maplexin J analogs, which in addition to the galloyl moiety, contained different numbers of phenolic groups at various positions intending to elucidate the effect of this functionality on the bioactivity. Phenol derivatives **45**–**48** [51,52,53] were prepared in 2-steps from commercially available methyl esters (Scheme 4). Subsequently, 1,5-AG was condensed with **16** [50], and **45**–**48** using DMAP, TEA, and 2-chloro-methylpyridinium iodide to obtain the corresponding Bn-protected 1,5-AG analogs **49**–**53**. Deprotection under hydrogenolysis conditions provided compounds **54**–**58** (Scheme 5). However, esterification of benzyl glucoside **59** [54] with **16** using 1-ethyl-3-(3-dimethylaminopropyl)carbodiimide (EDC) and DMAP resulted in the formation of intermediate **60**. Following hydrogenolysis, tetra-galloylated d-glucopyranose **61** containing a hemiacetal moiety was formed. Notably, the structure of **61 [55]** is analogous to tellimagrandin I; however, the biaryl bond of the HHDP group is missing (Scheme 6).

#### 2.1.3. Synthesis of a 1,5-AG-Based Tellimagrandin I Analog

**Scheme 7:**
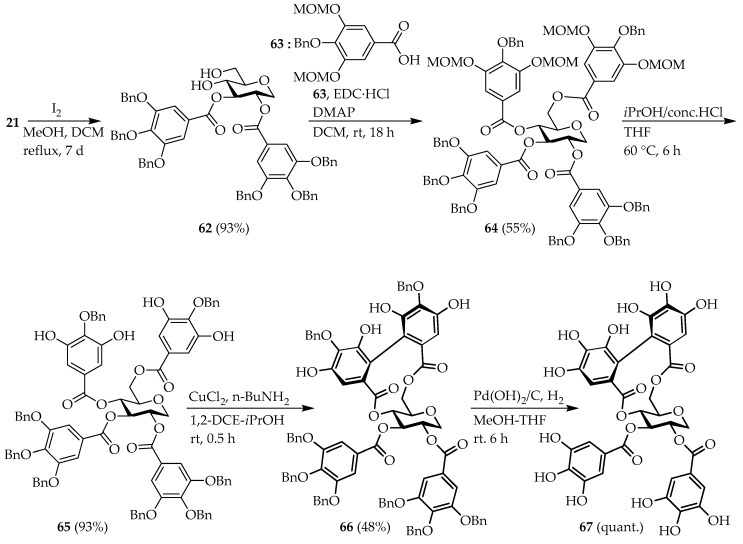
Synthesis of the 1,5-AG-based tellimagrandin I analog **67**.

To consider the effect of the hemiacetal group on the activity of tellimagrandin I, we also focused on the synthesis of the 1-deoxy analog **67**. Deprotection of benzylidene acetal in **21** in a methanol/DCM solvent system using iodine, according to the method reported by Feldman et al. [56], gave diol **62** in a high yield of 93% (Scheme 7). Intermediate **62** was then condensed with the gallic acid derivative **63** [57] to afford the galloylated compound **64**. The subsequent removal of the methoxymethyl (MOM) protecting groups provided **65** in 93% yield.

The construction of the HHDP group was performed in accordance with the approach previously reported by Yamada et al. [57,58,59,60]. Compound **65** was treated with ${n-\mathrm{BuNH}}_{2}$ and ${\mathrm{CuCl}}_{2}$ to provide the biaryl derivative **66**. Finally, hydrogenation of **66** in MeOH/THF with Pd(OH)_2_ as the catalyst gave the desired 1,5-AG-based tellimagrandin I analog **67**.

### 2.2. Evaluation of α-Glucosidase Inhibition and Antioxidant Activity

#### 2.2.1. The α-Glucosidase Inhibitory Activity

The α-glucosidase inhibitory activity of all samples was assayed utilizing a commercially available FUJIFILM α-glucosidase inhibitory activity assay kit. In total, 25 μL of each sample (25–2000 μg/mL in $H_{2}O$) or acarbose (0.5–8 μg/mL in $H_{2}O$), 50 μL of 18.5 mM maltose diluted in maleic anhydride buffer (100 mM, pH = 6.0), and 25 μL of the rat α-glucosidase solution were incubated in a micro-tube at 37 °C for 30 min. Subsequently, 400 μL of purified water was added to the solution and the reaction mixture was boiled for 3 min to deactivate α-glucosidase. The generated glucose was measured by LabAssay™ glucose (mutarotase-GOD method). In total, 100 μL of the reaction solution and 150 μL of the coloring solution were incubated in 96-well plates at 37 °C for 10 min, and the absorbance was recorded at 505 nm using a microplate reader (Bio-Rad, Model 680). The inhibition percentage was calculated using the following Equation:

$$\mathrm{Inhibition}\;\mathrm{percentage}\;\left(\%\right)\;=\;1-\frac{\mathrm{As}-\mathrm{Ab}}{\mathrm{Ac}}\;\times 100$$

where As is the absorbance of the analyzed sample, Ab is the absorbance of the blank (immediate deactivation), and Ac is the absorbance of the control (without α-glucosidase). Acarbose was used as the positive control.

#### 2.2.2. Evaluation of Antioxidant Activity

The antioxidant activity was assessed by employing the 2,2-diphenyl-1-picrylhydrazyl (DPPH) radical scavenging assay, according to the previously published method [61]. In total, 100 μL of each sample (1000–10 μg/mL in 50%EtOH/$H_{2}O$) or trolox (160–0 μM in 50% EtOH/$H_{2}O$), and 50 μL of 2-(*N*-morpholino)ethanesulfonic acid (MES)-NaOH buffer (200 mM, pH = 6.0), and 50 μL of an 800 μM DPPH diluted in 99.5% EtOH were mixed in 96-well plates. Following shaking the microplate for 15 min in the dark, the absorbance was recorded at 520 nm using a microplate reader (Bio-Rad, Model 680). Effective percentages (%) were calculated as follows:

$$\mathrm{Effective}\;\mathrm{percentages}\;\left(\%\right)\;=\;1-\frac{\mathrm{As}}{\mathrm{Ab}}\;\times 100$$

where As is the absorbance of the sample and Ab is the absorbance of the blank (without samples, 50% EtOH/$H_{2}O$ solution was used instead).

In addition, correlation of the absorbance on the *y*-axis and the concentrations on the *x*-axis resulted in the formation of an approximately straight-line plot. The trolox-equivalent (sample-mol/trolox-mol) was calculated as follows:

$$\mathrm{Trolox}-\mathrm{equivalent}\;(\frac{\mathrm{mol}-\mathrm{trolox}}{\mathrm{mol}-\mathrm{sample}})\;=\;\frac{\mathrm{Ss}}{\mathrm{St}}$$

where Ss is the slope of the sample and St is the slope of trolox.

## 3. Discussion

The results of the biological evaluation of the 21 synthesized compounds considering the α-glucosidase inhibitory and antioxidant activities are summarized in Table 1.

The comparison of the 1,5-AG-based polyphenol analogs **31**–**44** and **54** revealed that the α-glucosidase inhibitory activity significantly increased with the number of the galloyl units in the compounds and the highest inhibition was observed for tetra-*O*-galloyl-1,5-AG (maplexin J) **54** (IC_50_ = 2.56 μM). This result is in accordance with the previous reports [32]. In addition, different the position of the galloyl unit on the 1,5-AG core appeared to influence the α-glucosidase inhibitory activity, even for the compounds with the same number of these moieties. Analogous outcomes were noted for 2-galloyl-1,5-AG (**31**) and methyl gallate (IC_50_ = 95.1, 90.5 μM, respectively). Conversely, differing α-glucosidase inhibitory activities were obtained for mono-galloyl analogs **32**–**34**, which are weaker inhibitors than methyl gallate (IC_50_ = 127.1–143.9 μM). Furthermore, compound **33** (galloyl unit at the C-4 position) exhibited lowest activity (IC_50_ = 143.9 μM). Higher inhibitory activity was detected for di-galloylated analogs **35**–**37** (IC_50_ = 20.5–48.3 μM), which possessed the galloyl unit at the C-2 position than for analogs **38**–**40** (IC_50_ = 26.6–91.4 μM). In particular, compound **35** that contains esterified galloyl units at the C-2 and C-3 positions exhibited three-times higher inhibitory activity than gallic acid. Moreover, analog **38**, galloylated at the C-3 and C-4 positions, displayed significantly lower activity (IC_50_ = 91.4). Among the tri-galloyllated analogs, compound **42** without a galloyl unit at the C-4 position showed stronger inhibitory activity (IC_50_ = 5.34 μM) than analogs **41**, **43**, and **44** (IC_50_ = 6.72, 9.34, and 12.6 μM, respectively). In addition, analog **44** without a galloyl moiety at the C-2 position displayed weak activity. Thus, our results suggested that a galloyl unit at the C-2 position considerably increases the α-glucosidase inhibitory activity, while the presence of this group at the C-4 position causes a decrease in the activity.

Subsequently, we compared maplexin J (**54**) and its analogs (**55** and **56**) to elucidate the effect of the phenolic hydroxyl group. The 3′,4′-di-hydroxybenzoyl analog **55** exhibited good inhibitory activity, whereas the 3′,5′-di-hydroxybenzoyl analog **56** was a weaker inhibitor of the α-glucosidase enzyme (IC_50_ = 3.28, 9.34 μM, respectively). Consequently, these results implied that the presence of two adjacent phenolic hydroxyl groups is essential for the desired activity. We then focused on the evaluation of the influence of the hemiacetal hydroxyl and the HHDP functionalities against the α-glucosidase inhibitory activity. The effect of the hemiacetal hydroxyl moiety can be observed by the comparison of the activity of maplexin J (**54**) and its analog **61** (IC_50_ = 1.68, 2.56 μM, respectively). Furthermore, tellimagrandin I and **67** showed analogous inhibitory activity (IC_50_ = 3.37, 3.22 μM, respectively). Therefore, the obtained outcomes suggested that the presence of a hemiacetal hydroxyl group did not have a significant effect on the α-glucosidase inhibitory activity. Lastly, to examine the influence of the HHDP group, the results for maplexin J (**54**) and the 4,6-*O*-HHDP analog **67** were compared and it transpired that maplexin J (**54**) displayed marginally higher activity than its analog **67** (IC_50_ = 2.56, 3.22 μM, respectively). Likewise, the assessment of the tellimagrandin I activity and the activity of analog **61** without the HHDP group revealed that **61** was a stronger inhibitor than tellimagrandin I (IC_50_ = 3.37, 1.68 μM, respectively). Intriguingly, our results suggested that the 4,6-*O*-HHDP group has a weakening effect on the α-glucosidase inhibitory activity [38,62].

Meanwhile, we also investigated the antioxidant activity of these polyphenols and their analogs. Firstly, the mono-galloylated analogs **31**–**34** showed nearly equivalent antioxidative activity with methyl gallate (EC_50_ = 19.4–22.9 μM, TE = 2.04–2.42). Moreover, the activity improved as the number of galloyl units increased, with di-galloylated analogs (EC_50_ = 11.9–15.2 μM, TE = 3.32–4.29) exhibiting lower activity than the tri-galloylated analogs (EC_50_ = 7.94–10.3 μM, TE = 4.31–5.75) and the tetra-galloylated analogs **54**, **61** displaying the highest activity (EC_50_ = 5.60, 6.61 μM, TE = 8.33, 6.77, respectively). Unlike the significant increase observed for the α-glucosidase inhibitory activity, the antioxidant activity increased proportionally to the number of galloyl units. Moreover, the antioxidant activity was not affected by the position of the galloyl units on the carbohydrate core. In addition, the comparison of compounds **54**, **61**, and **67** with tellimagrandin I revealed similar activity (EC_50_ = 6.61, 5.60, 6.79, and 6.31 μM, TE = 6.77, 8.33, 6.44, and 7.04, respectively). This implied that the antioxidative activity was not influenced by the presence of a hemiacetal hydroxyl and the 4,6-*O*-HHDP groups. The presence of the phenolic hydroxyl groups in 3,4-dihydroxybenzoyl analogs **55** and methyl 3,4-dihydroxybenzoate appeared to result in improved antioxidative activity (EC_50_ = 6.99 μM, TE = 6.54); however, no antioxidant activity was observed for 3,5-dihydroxybenzoyl analog **56** and the monohydroxybenzoyl analogs (**57** and **58)**. Likewise, the activity of methyl 3,4-dihydroxybenzoate was comparable with methyl gallate (EC_50_ = 21.1 μM, TE = 2.20), whereas methyl 3,5-dihydroxybenzoate, methyl 3- and 4-hydroxybenzoate did not exhibit any notable activity. This data therefore suggested that the presence of two adjacent phenolic hydroxyl groups is necessary for the antioxidant activity, as was the case with α-glucosidase inhibitory activity.

## 4. Conclusions

We synthesized 21 carbohydrate-based phenolic analogs including a series of compounds containing all possible combinations of galloylation on 1,5-AG. The α-glucosidase inhibition and antioxidant activities of these compounds were further studied to evaluate the SAR. Our results suggested that the α-glucosidase inhibitory activity; 1) is significantly enhanced with the increasing number of galloyl units, and changing the position of the galloyl moiety substitution on the 1,5-AG unit tends to affect the activity; particularly, the presence of this functionality at the C-2 position improves the α-glucosidase inhibition, whereas substitution at the C-4 position reduces it, 2) requires two adjacent phenolic hydroxyl groups, 3) is not affected by the presence of the biaryl bond on the 4,6-*O*-HHDP group, 4) is not influenced by the hemiacetal hydroxyl functionality on the carbohydrate unit. Moreover, the following trends were determined for the antioxidant activity; 1) the activity is dependent on the number of galloyl units; however, it is not affected by their position, 2) the presence of two adjacent phenolic hydroxyl groups is significant, 3) the activity is not affected by the HHDP group or the hemiacetal hydroxyl group. The α-glucosidase inhibitory activity is undoubtedly affected by the position of the galloyl group on 1,5-AG. This outcome indicates that the carbohydrate core is not only a store unit for the galloyl moiety but can also act as a carrier to the biological targets. Thus, derivatives modified at the C-2 or C-4 positions on the carbohydrate unit, which is not d-glucose, have the potential to exhibit stronger antidiabetic activity. The synthesis and profiling of further analogs will be reported in due course.

## 5. Materials and Methods

### 5.1. General Information

Proton nuclear magnetic resonance (H1 NMR) spectra were recorded on JEOL JNM-ECX600 spectrometers. Chemical shifts are reported relative to internal standard (tetramethylsilane; $\delta_{H}$ 0.00, ${\mathrm{CDCl}}_{3}$; $\delta_{H}$ 7.26). Data are presented as follows: chemical shift (δ, ppm), multiplicity (s = singlet, d = doublet, t = triplet, q = quartet, m = multiplet), coupling constant and integration. Carbon nuclear magnetic resonance (C13 NMR) spectra were recorded on JEOL JNM-ECX600 (150 MHz) spectrometers. The following internal reference was used: (tetramethylsilane: δ 0.00, ${\mathrm{CDCl}}_{3}$ δ 77.0, ${\mathrm{Acetone}-d}_{6}$; δ 29.8, ${\mathrm{CD}}_{3}\mathrm{OD}$; δ 49.0). Optical rotations were measured on a JASCO P-1030 digital polarimeter at the sodium D line (589 nm). Electron impact (EI) mass analyses and fast atom bombardment (FAB) mass analyses were carried out with a JEOL JMS-GCMATE. C.C. was carried out on Kanto Silica gel 60N spherical (63–210 mesh). Analytical thin-layer chromatography (TLC) was carried out using Merck Kieselgel 60 F254 plates with visualization by ultraviolet light or stained by 8% $H_{2}{\mathrm{SO}}_{4}$/EtOH solution on hot-plate. Tellimagrandin I was purchased from Nagara Science (Gifu, Japan). Methyl gallate, methyl 3,4-dihydroxybenzoate, methyl 3,5-dihydroxybenzoate, methyl 3-hydroxybenzoate, methyl 4-hydroxybenzoate, Trolox and MES were purchased from TOKYO CHEMICAL INDUSTRY CO., LTD (Tokyo, Japan). DPPH was purchased from Sigma-Aldrich Japan (Tokyo, Japan).

### 5.2. Chemical Synthesis

*2-O-Benzyl-4,6-O-benzylidene-1,5-anhydro-d-glucitol* (**3**) [35] and *3-O-Benzyl-4,6-O-benzylidene-1,5-anhydro-d-glucitol* (**4**) [35]; Compound **1** (2.5 g, 10 mmol) and ${\;\mathrm{TBAHSO}}_{4}$ (0.68 g, 2.0 mmol) in 160 mL of DCM and 14 mL of 5% NaOH was stirred at rt. Then, BnBr (2.1 mL, 17 mmol) was slowly added, and the mixture was refluxed for 30 h. After the addition 50 mL of water, the mixture was extracted with DCM (3 × 100). The combined organic layers were washed with brine, dried over ${\mathrm{Na}}_{2}{\mathrm{SO}}_{4}$, filtered and concentrated under reduced pressure. The crude product was separated by C.C (Hex/EtOAc = 4/1) to obtain 2-*O*-Bn compound **3** (2.0 g, 59%) as colorless needles and 3-*O*-Bn compound **4** (1.0 g, 30% yield) as a white solid. Compound **3**: m.p. 163 °C; ${\left[\alpha \right]}_{D}^{20}$ = −3.16 (c 1.0, ${\mathrm{CHCl}}_{3}$); H1 NMR (${\mathrm{CDCl}}_{3}$, 600 MHz) δ 7.49–7.47 (m, 2H), 7.38–7.29 (m, 8H), 5.50 (s, 1H), 4.76, 4.67 (ABq, *J* = 11.7 Hz, 2H), 4.30 (dd, *J* = 10.5, 5.0 Hz, 1H), 4.01 (dd, *J* = 11.3, 5.5 Hz, 1H), 3.84 (m, 1H), 3.65 (t, *J* = 10.3 Hz, 1H), 3.59–3.55 (m, 1H), 3.45 (t, *J* = 9.3 Hz, 1H), 3.35 (m, 1H), 3.30 (t, *J* = 11.0 Hz, 1H); C13 NMR (${\mathrm{CDCl}}_{3}$, 150 MHz) δ 138.02, 137.05, 129.23, 128.56, 128.33, 128.03, 127.89, 126.29, 101.88, 81.05, 77.74, 74.81, 73.43, 70.91, 68.79, 68.45. Compound **4**: m.p. 137 °C; ${\left[\alpha \right]}_{D}^{20}$ = +5.3 (c 1.0, ${\mathrm{CHCl}}_{3}$); H1 NMR (${\mathrm{CDCl}}_{3},$ 600 MHz) δ 7.50–7.48 (m, 2H), 7.41–7.29 (m, 8H), 5.58 (s, 1H), 5.03, 4.72 (ABq, *J* = 11.3 Hz, 2H), 4.34 (dd, *J* = 10.5, 5.0 Hz, 1H), 4.06 (dd, *J* = 11.2, 5.7 Hz, 1H), 3.80–3.76 (m, 1H), 3.72 (t, *J* = 10.3 Hz, 1H), 3.66 (t, *J* = 9.1 Hz, 1H), 3.58 (t, *J* = 8.8 Hz, 1H), 3.44–3.40 (m, 1H), 3.34 (t, *J* = 10.8 Hz, 1H); C13 NMR (${\mathrm{CDCl}}_{3}$, 150 MHz) δ 138.30, 137.32, 129.00, 128.61, 128.30, 128.13, 128.01, 125.98, 101.21, 82.66, 82.13, 74.71, 71.53, 69.91, 69.79, 68.88.

*2,3-Di-O-benzyl-1,5-anhydro-d-glucitol* (**5**) [42]; Compound **2** (0.86g, 2.0 mmol) in 10 mL of 80% AcOH/$H_{2}O$ solution was stirred at 80 °C for 5 h. After the addition 5 mL of saturated aq. ${\mathrm{NaHCO}}_{3}$, the reaction solution was extracted by EtOAc (3 × 30). The organic layer was washed with brine, dried over ${\;\mathrm{Na}}_{2}{\mathrm{SO}}_{4}$, filtered and concentrated under reduced pressure. The crude was purified by recrystallization (EtOAc/Hex) to obtain **5** (0.65 g, 95%) as colorless needles. m.p. 129 °C; ${\left[\alpha \right]}_{D}^{20}$ = −10.4 (c 1.00, ${\mathrm{CHCl}}_{3}$); H1 NMR (${\mathrm{CDCl}}_{3}$, 600 MHz) δ 7.43–7.29 (m, 10H), 5.03, 4.70 (ABq, *J* = 11.7 Hz, 2H), 4.64, 4.03 (ABq, *J* = 11.3, 2H), 3.86–3.82 (m, 1H), 3.72–3.68 (m, 1H), 3.63–3.59 (m, 1H), 3.50–3.44 (m, 2H), 3.28–3.23 (m, 2H); C13 NMR (${\mathrm{CDCl}}_{3}$, 150 MHz) δ 138.54, 137.97, 128.67, 128.53, 127.98, 127.93, 127.83, 85.30, 79.53, 78.29, 75.10, 73.07, 70.45, 67.94, 62.89.

*1,5-Anhydro-2-O-benzyl-d-glucitol* (**6**) [35]; Compound **3** (860 mg, 2.5 mmol) in 10 mL of 80% AcOH/$H_{2}O$ solution was stirred at 80 °C for 5 h. By the same procedure previously described for the preparation of compound **5**, compound **6** (503 mg, 76%) was obtained as colorless needles. m.p. 131 °C; ${\left[\alpha \right]}_{D}^{20}$ = +8.4 (c 1.00, MeOH); H1 NMR (${\mathrm{CD}}_{3}\mathrm{OD}$, 600 MHz); δ 7.40–7.36 (m, 2H), 7.33–7.31 (m, 2H), 7.28–7.25 (m, 1H), 4.75, 4.64 (ABq, *J* = 11.7 Hz, 2H), 3.96 (dd, *J* = 11.0, 5.2 Hz, 1H), 3.81 (dd, *J* = 11.9, 2.2 Hz, 1H), 3.59 (dd, *J* = 11.9, 6.0 Hz, 1H), 3.43 (t, *J* = 8.9 Hz, 1H), 3.39–3.35 (m, 1H), 3.26–3.22 (m, 1H), 3.15–3.11 (m, 2H); C13 NMR (${\mathrm{CD}}_{3}\mathrm{OD}$, 150 MHz); δ 140.09, 129.37, 129.05, 128.76, 82.42, 79.31, 79.27, 74.14, 71.92, 68.95, 63.07.

*1,5-Anhydro-3-O-benzyl-d-glucitol* (**7**); Compound **4** (680 mg, 2.0 mmol) in 10 mL of 80% AcOH/$H_{2}O$ solution was stirred at 80 °C for 5 h. By the same procedure previously described for the preparation of compound **5**, compound **7** (410 mg, 80%) was obtained as colorless needles. m.p. 153 °C; ${\left[\alpha \right]}_{D}^{20}$ = +28.6 (c 1.00, MeOH); H1 NMR (${\mathrm{CD}}_{3}\mathrm{OD}$, 600 MHz); δ 7.44 (m, 2H), 7.32–7.30 (m, 2H), 7.26–7.23 (m, 1H), 4.90 (overlap, 2H), 3.89 (dd, *J* = 11.3, 5.5 Hz, *1*H), 3.83 (dd, *J* = 11.9, 2.2 Hz, 1H), 3.62–3.57 (m, 2H), 3.37 (dd, *J* = 18.0, 8.71 Hz, 1H), 3.31–3.28 (overlap, 1H), 3.22–3.16 (m, 2H); C13 NMR (${\mathrm{CD}}_{3}\mathrm{OD}$, 150 MHz); δ 140.53, 129.20, 129.09, 128.49, 88.20, 82.68, 76.10, 71.67, 71.54, 71.09, 63.05. HRMS (ESI, *m/z*): ${\left[M+\mathrm{Na}\right]}^{+}$, calcd for ${{[C}_{13}H_{18}O_{5}\mathrm{Na}]}^{+}$: 277.1052; found 277.1052.

*1,5-Anhydro-4-O-benzyl-d-glucitol* (**8**) [35]; Compound **1** (0.76 g, 3.0 mmol) in 15 mL of DCM was stirred at 0 °C. Then, 15 mL of borane-THF (ca. 1M THF solution) and TMSOTf (0.11 mL, 0.60 mmol) were successively added to the mixture. The mixture was allowed to stir for 4 h and added MeOH carefully. After the addition 1 mL of saturated aq. ${\mathrm{NaHCO}}_{3}$, the reaction solution was extracted with DCM (5 × 40 mL). The combined organic layer was dried over ${\mathrm{Na}}_{2}{\mathrm{SO}}_{4}$, filtered and concentrated under reduced pressure. The crude product was purified by C.C. $({\mathrm{CHCl}}_{3}$/MeOH = 8/1) to obtain **8** (0.64 g, 74%) as a white solid. ${\left[\alpha \right]}_{D}^{20}$ = +27.7 (c 0.45, ${\mathrm{CHCl}}_{3}$); H1 NMR (${\mathrm{CD}}_{3}\mathrm{OD}$, 600 MHz) δ 7.38–7.25 (m, 5H), 4.94, 4.64 (ABq, *J* = 11.0 Hz, 2H), 3.89 (dd, *J* = 13.3, 5.4 Hz, 1H), 3.78 (dd, *J* = 12.0, 2.1 Hz, 1H), 3.60 (dd, 1H, *J* = 11.4, 5.73 Hz,), 3.49–3.46 (m, 2H), 3.33–3.30 (overlap, 1H), 3.20 (ddd, *J* = 9.7, 5.2, 2.1 Hz, 1H), 3.14 (t, *J* = 10.7, 1H); C13 NMR (${\mathrm{CD}}_{3}\mathrm{OD}$, 150 MHz); δ 140.09, 129.33, 129.14, 128.70, 81.75, 80.43, 79.50, 75.90, 71.77, 70.93, 62.72.

*2,4-Di-O-benzyl-1,5-anhydro-d-glucitol* (**9**) [46]; Compound **3** (1.0 g, 3.0 mmol) in 15 mL of DCM was successively added 15 mL of borane-THF (ca. 1M THF solution) and TMSOTf (0.11 mL, 0.6 mmol) at 0 °C. The mixture was allowed to stir at rt for 10 h and added 1 mL of MeOH carefully. After the addition 1 mL of saturated aq. ${\mathrm{NaHCO}}_{3}$, the mixture was extracted with DCM (3 × 40 mL). The organic layer was washed with brine, dried over ${\mathrm{Na}}_{2}{\mathrm{SO}}_{4}$, filtered and concentrated under reduced pressure. The crude was purified by recrystallization (Hex/EtOAc) to obtain **9** (820 mg, 82%) as colorless needles. m.p. 112 °C; ${\left[\alpha \right]}_{D}^{20}$ = +34.6 (c 1.00, ${\mathrm{CHCl}}_{3}$); H1 NMR (${\mathrm{CDCl}}_{3}$, 600 MHz) δ 7.37–7.28 (m, 10H), 4.86, 4.70 (ABq, *J* = 11.3 Hz, 2H), 4.65 (s, 2H), 3.99 (dd, *J* = 11.3, 5.2 Hz, 1H), 3.84 (ddd, *J* = 11.8, 5.8, 2.7 Hz, 1H), 3.74 (td, *J* = 8.9, 2.1 Hz, 1H), 3.68–3.64 (m, 1H), 3.45–3.41 (m, 1H), 3.41 (t, *J* = 9.2 Hz, 1H), 3.26 (ddd, *J* = 9.6, 4.5, 2.7 Hz, 1H), 3.19 (t, *J* = 10.8 Hz, 1H); C13 NMR (${\mathrm{CDCl}}_{3}$, 150 MHz) δ 138.18, 138.00, 128.60, 128.56, 128.09, 127.98, 127.85, 79.34, 78.18, 77.97, 77.39, 74.74, 73.05, 67.44, 62.27.

*3,4-Di-O-benzyl-1,5-anhydro-d-glucitol* (**10**) [47]; Compound **4** (850 g, 2.5 mmol) in 15 mL of DCM was successively added 12.5 mL of borane-THF (ca. 1M THF solution) and TMSOTf (90 μL, 0.5 mmol) at 0 °C. The mixture was allowed to stir at rt for 7 h and added 5 mL of MeOH carefully. After the addition 1 mL of saturated aq. ${\mathrm{NaHCO}}_{3}$, the mixture was extracted with DCM (3 × 40 mL). The organic layer was washed with brine, dried over ${\mathrm{Na}}_{2}{\mathrm{SO}}_{4}$, filtered and concentrated under reduced pressure. The crude was purified by recrystallization (Hex/EtOAc) to obtain **10** (523 mg, 61%) as colorless needles. m.p. 99 °C; ${\left[\alpha \right]}_{D}^{20}$ = +48.3 (c 1.40, ${\mathrm{CHCl}}_{3}$); H1 NMR (${\mathrm{CDCl}}_{3}$, 600 MHz) δ 7.38–7.30 (m, 10H), 4.97, 4.77 (ABq, *J* = 11.3 Hz, 2H), 4.86, 4.68 (ABq, *J* = 11.0 Hz, 2H), 3.98 (dd, *J* = 11.2, 5.3 Hz, 1H), 3.84 (ddd, *J* = 11.9, 5.5, 2.6 Hz, 1H), 3.71–3.65 (m, 2H), 3.52 (t, *J* = 9.1 Hz, 1H), 3.47 (t, *J* = 8.8 Hz, 1H), 3.32–3.29 (m, 1H), 3.23 (t, *J* = 10.8 Hz, 1H); C13 NMR (${\mathrm{CDCl}}_{3}$, 150 MHz) δ 138.45, 137.84, 128.71, 128.56, 128.02, 127.86, 86.73, 79.92, 77.79, 75.25, 74.95, 70.16, 69.30, 61.99.

*2,3,4-Tri-O-benzyl-1,5-anhydro-d-glucitol* (**11**) [46]; Compound **2** (860 mg, 2.0 mmol) in 10 mL of toluene was stirred at rt. The reaction solution was added DIBAL-H (ca. 1M toluene solution, 6 mL) and stirred at rt for 20 h. The reaction solution was slowly added 4.2 mL of MeOH and 7.2 mL of 30% Rochelle salt solution and stirred for 1 h. After the addition 20 mL of EtOAc, the mixture was extracted with 30% Rochelle salt solution (3 × 15 mL). The organic layer was washed with brine, dried over ${\;\mathrm{Na}}_{2}{\mathrm{SO}}_{4}$, filtered and concentrated under reduced pressure. The crude was purified by C.C. (Hex/EtOAc = 4/1–2/1) to obtain **11** (760 mg, 90% yield) as colorless needles. m.p. 83 °C; ${\left[\alpha \right]}_{D}^{20}$ = +8.9 (c 0.90, ${\mathrm{CHCl}}_{3}$); H1 NMR (${\mathrm{CDCl}}_{3}$, 600 MHz) δ 7.37–7.28 (m, 15H), 4.98–4.64 (m, 6H), 3.99 (dd, *J* = 11.3, 5.2 Hz, 1H), 3.84–3.81 (m, 1H), 3.67–3.58 (m, 3H), 3.48 (t, *J* = 9.3 Hz, 1H), 3.29–3.25 (m, 1H), 3.23 (t, *J* = 10.8 Hz, 1H); C13 NMR (${\mathrm{CDCl}}_{3}$, 150 MHz) δ 138.61, 138.09, 138.01, 128.48, 128.41, 128.05, 127.89, 127.83, 127.64, 86.17, 79.69, 78.55, 77.57, 75.55, 75.13, 73.34, 67.96, 62.25.

*1,5-Anhydro-6-O-benzyl-d-glucitol* (**12**) [35]; Compound **1** (760 mg, 3.0 mmol) in 15 mL of DCM was added triethylsilane (2.4 mL, 15 mmol) and trifluoracetic acid (1.2 mL, 15 mmol) at 0 °C. The reaction solution was stirred at rt for 8 h. After the addition 10 mL of saturated aq. ${\mathrm{NaHCO}}_{3}$, the mixture was extracted with DCM (5 × 30 mL). The combined organic layer was dried over ${\;\mathrm{Na}}_{2}{\mathrm{SO}}_{4}$, filtered and concentrated under reduced pressure. The crude was purified by C.C (${\mathrm{CHCl}}_{3}$/MeOH = 100/1–10/1) to obtain **12** (471 mg, 62%) as a colorless oil. ${\left[\alpha \right]}_{D}^{20}$ = +8.9 (c 0.30, ${\mathrm{CHCl}}_{3}$); H1 NMR (${\mathrm{CDCl}}_{3}$, 600 MHz) δ 7.35–7.25 (m, 5H), 4.55 (d, *J* = 2.1 Hz, 2H), 3.88 (dd, *J* = 11.0, 5.5 Hz, 1H), 3.77 (dd, *J* = 10.8, 1.9 Hz, 1H), 3.60 (dd, *J* = 10.8, 5.7 Hz, 1H), 3.48–3.42 (m, 1H), 3.32–3.24 (overlap, 3H), 3.15 (t, *J* = 10.8 Hz, 1H); C13 NMR (${\mathrm{CDCl}}_{3}$, 150 MHz) δ 139.57, 129.34, 128.90, 128.67, 81.38, 79.95, 74.48, 71.85, 71.31, 71.19, 70.95.

*2,3,6-Tri-O-benzyl-1,5-anhydro-d-glucitol* (**13**) [49]; Compound **2** (860 mg, 2.0 mmol) in 10 mL of DCM was added triethylsilane (1.6 mL, 10 mmol) and tifluoroacetic acid (0.8 mL, 10 mmol) at 0 °C. The reaction solution was stirred at rt for 15 h. After the addition 5 mL of saturated aq. ${\mathrm{NaHCO}}_{3}$, the mixture was extracted with DCM (3 × 30 mL). The organic layer was washed with brine, dried over ${\;\mathrm{Na}}_{2}{\mathrm{SO}}_{4}$, filtered and concentrated under reduced pressure. The crude product was purified by C.C. (Hex/EtOAc = 4/1) to obtain **13** (505 mg, 59%) as a colorless oil. ${\left[\alpha \right]}_{D}^{20}$ = −7.0 (c 1.00, ${\mathrm{CHCl}}_{3}$); H1 NMR (${\mathrm{CDCl}}_{3}$, 600 MHz) δ 7.37–7.25 (m, 15H), 5.00, 4.76 (ABq, *J* = 11.5 Hz, 2H), 4.69, 4.63 (ABq, *J* = 11.7 Hz, 2H), 4.58, 4.54 (ABq, *J* = 12.2 Hz, 2H), 4.04 (dd, *J* = 11.3, 5.2 Hz, 1H), 3.70 (dd, *J* = 10.5, 3.3 Hz, 1H), 3.64–3.60 (m, 2H), 3.54 (td, *J* = 9.2, 2.1 Hz, 1H), 3.44 (t, *J* = 8.9 Hz, 1H), 3.37–3.34 (m, 1H), 3.23 (t, *J* = 11.0 Hz, 1H); C13 NMR (${\mathrm{CDCl}}_{3}$, 150 MHz) δ 138.64, 138.05, 137.84, 128.58, 128.48, 128.39, 127.95, 127.90, 127.87, 127.83, 127.77, 127.70, 85.40, 78.75, 78.04, 75.10, 73.65, 73.05, 70.87, 69.98, 68.06.

*2,6-Di-O-benzyl-1,5-anhydro-d-glucitol* (**14**); Compound **3** (860 mg, 2.5 mmol) in 15 mL of DCM was added triethylsilane (2.0 mL, 12.5 mmol) and tifluoroacetic acid (1.0 mL, 12.5 mmol) at 0 °C. The reaction solution was stirred at rt for 8 h. After the addition 10 mL of saturated aq. ${\mathrm{NaHCO}}_{3}$, the mixture was extracted with DCM (3 × 40 mL). The organic layer was washed with brine, dried over ${\;\mathrm{Na}}_{2}{\mathrm{SO}}_{4}$, filtered and concentrated under reduced pressure. The crude product was purified by C.C. $({\mathrm{CHCl}}_{3}$/MeOH = 8/1) to obtain **14** (500 mg, 58%) as a white solid. ${\left[\alpha \right]}_{D}^{20}$ = +17.4 (c 1.00, ${\mathrm{CHCl}}_{3}$); H1 NMR (${\mathrm{CDCl}}_{3}$, 600 MHz) δ 7.36–7.27 (m, 10H), 4.64 (s, 2H), 4.59, 4.54 (ABq, *J* = 12.0 Hz, 2H), 4.02 (dd, *J* = 11.2, 5.0 Hz, 1H), 3.69 (dd, *J* = 10.5, 3.6 Hz, 1H), 3.65 (dd, *J* = 10.5, 5.0 Hz, 1H), 3.55 (td, *J* = 8.8, 2.1 Hz, 1H), 3.50 (td, *J* = 9.0, 2.6 Hz, 1H), 3.47–3.43 (m, 1H), 3.37–3.34 (m, 1H), 3.19 (t, *J* = 10.8 Hz, 1H); C13 NMR (${\mathrm{CDCl}}_{3}$, 150 MHz) δ 137.96, 137.71, 128.59, 128.46, 128.07, 127.90, 127.81, 78.31, 77.74, 77.41, 73.68, 72.95, 71.34, 70.01, 67.70. HRMS (ESI, *m/z*): ${\left[M+\mathrm{Na}\right]}^{+}$, calcd for ${{[C}_{20}H_{24}O_{5}\mathrm{Na}]}^{+}$: 367.1521; found 367.1521.

*1,5-Anhydro-3,6-di-O-benzyl-d-glucitol* (**15**); Compound **4** (680 mg, 2.0 mmol) in 15 mL of DCM was added triethylsilane (1.6 mL, 10 mmol) and trifluoroacetic acid (0.80 mL, 10 mmol) at 0 °C. The reaction solution was stirred at rt for 4 h. After the addition 10 mL of saturated aq. ${\mathrm{NaHCO}}_{3}$, the mixture was extracted with DCM (3 × 40 mL). The combined organic layer was washed with brine, dried over ${\;\mathrm{Na}}_{2}{\mathrm{SO}}_{4}$, filtered and concentrated under reduced pressure. The crude product was purified by C.C. $({\mathrm{CHCl}}_{3}$/MeOH = 8/1) to obtain **15** (420 mg, 62%) as a white solid. ${\left[\alpha \right]}_{D}^{20}$ = +22.7 (c 1.00, ${\mathrm{CHCl}}_{3}$); H1 NMR (${\mathrm{CDCl}}_{3}$, 600 MHz) δ 7.39–7.27 (m, 10H), 4.91, 4.83 (ABq, *J* = 11.7 Hz, 2H), 4.59, 4.54 (ABq, *J* = 12.2 Hz, 2H), 3.96 (dd, *J* = 11.2, 5.3 Hz, 1H), 3.72–3.66 (m, 3H), 3.61 (dt, *J* = 9.1, 2.6 Hz, 1H), 3.39–3.36 (m, 1H), 3.31 (t, *J* = 8.8 Hz, 1H), 3.21 (t, *J* = 10.8 Hz, 1H); C13 NMR (${\mathrm{CDCl}}_{3}$, 150 MHz) δ 138.60, 137.61, 128.70, 128.47, 128.02, 127.93, 127.86, 127.82, 86.47, 78.47, 74.87, 73.74, 72.11, 70.38, 69.79, 69.57. HRMS (ESI, *m/z*): ${\left[M+\mathrm{Na}\right]}^{+}$, calcd for ${{[C}_{20}H_{24}O_{5}\mathrm{Na}]}^{+}$: 367.1521; found 367.1522.

*1,5-Anhydro-2-O-(3′,4′,5′-tribenzyloxybenzoyl)-3-O-benzyl-4,6-O-benzylidene-d-glucitol* (**17**); Compound **3** (340 mg, 1.0 mmol), compound **16** [39] (650 mg, 1.5 mmol), 2-chloro-1-methylpyridinium iodide (380 mg, 1.5 mmol), DMAP (37 mg, 0.30 mmol), TEA (416 μL, 3.0 mmol) in 15 mL of DCM was stirred at rt for 20 h. After the addition 100 mL of saturated aq. ${\mathrm{NH}}_{4}\mathrm{Cl}$, the reaction solution was extracted with DCM (3 × 60 mL). The combined organic layer was washed with brine, dried over ${\mathrm{Na}}_{2}{\mathrm{SO}}_{4}$, filtered and concentrated under reduced pressure. The crude was purified by C.C. (DCM/MeOH = 500/1 − 200/1) to obtain **17** (350 mg, 41%) as a colorless amorphous oil. ${\left[\alpha \right]}_{D}^{20}$ = +8.4 (c 1.04, ${\mathrm{CHCl}}_{3}$); H1 NMR (${\mathrm{CDCl}}_{3}$, 600 MHz) δ 7.53–7.11 (m, 29H), 5.61 (s, 1H), 5.27–5.20 (m, 1H), 5.15 (s, 2H), 5.09 (s, 4H), 4.86, 4.71 (ABq, *J* = 12.0 Hz, 2H), 4.36 (dd, *J* = 10.3, 4.8 Hz, 1H), 4.19 (dd, *J* = 11.0, 5.8 Hz, 1H), 3.87 (t, *J* = 9.1 Hz, 1H), 3.79–3.74 (m, 2H), 3.45 (td, *J* = 9.7, 4.9 Hz, 1H), 3.36 (t, *J* = 10.8 Hz, 1H); C13 NMR (${\mathrm{CDCl}}_{3}$, 150 MHz) δ 165.06, 152.55, 142.74, 138.10, 137.31, 137.28, 136.56, 129.03, 128.57, 128.30, 128.25, 128.22, 128.07, 128.02, 127.89, 127.58, 127.47, 126.02, 124.50, 109.41, 101.32, 81.96, 79.12, 75.14, 74.33, 71.50, 71.30, 68.76, 67.68; HRMS (ESI, *m/z*): ${\left[M+\mathrm{Na}\right]}^{+}$, calcd for ${{[C}_{48}H_{44}O_{9}\mathrm{Na}]}^{+}$: 787.2883; found 787.2881.

*1,5-Anhydro-2-O-benzyl-3-O-(3′,4′,5′-tribenzyloxybenzoyl)-4,6-O-benzylidene-d-glucitol* (**18**); Compound **2** (0.51 g, 1.5 mmol), compound **16** (1.0 g, 2.3 mmol), 2-chloro-1-methylpyridinium iodide (0.59 g, 2.3 mmol), DMAP (28 mg, 0.23 mmol), TEA (0.62 mL, 4.5 mmol) in 20 mL of DCM was stirred at rt for 20 h. By the same procedure previously described for the preparation of compound **17**, compound **18** (0.87 g, 68%) was obtained as a colorless amorphous oil. ${\left[\alpha \right]}_{D}^{20}$ = −45.6 (c 0.95, ${\mathrm{CHCl}}_{3}$); H1 NMR (${\mathrm{CDCl}}_{3}$, 600 MHz) δ 7.24–7.44 (m, 24H), 7.20–7.14 (m, 5H), 5.53 (t, *J* = 9.3 Hz, 1H), 5.46 (s, 1H), 5.15–5.10 (m, 6H), 4.55, 4.46 (ABq, *J* = 12.4 Hz, 2H), 4.35 (dd, *J* = 10.5, 5.0 Hz, 1H), 4.13 (dd, *J* = 11.3, 5.5 Hz, 1H), 3.76–3.72 (m, 1H), 3.70 (t, *J* = 10.3 Hz, 1H), 3.65 (t, *J* = 9.5 Hz, 1H), 3.51 (td, *J* = 9.7, 4.8 Hz, 1H), 3.48 (t, *J* = 11.0 Hz, 1H); C13 NMR (${\mathrm{CDCl}}_{3}$, 150 MHz) δ 165.16, 152.47, 142.58, 137.53, 137.39, 136.69, 128.95, 128.54, 128.38, 128.21, 128.16, 128.02, 127.98, 127.87, 127.53, 126.14, 125.05, 109.55, 101.35, 79.18, 75.62, 75.15, 74.94, 72.97, 71.48, 71.33, 68.80, 68.74; HRMS (ESI, *m/z*): ${\left[M+\mathrm{Na}\right]}^{+}$, calcd for ${{[C}_{48}H_{44}O_{9}\mathrm{Na}]}^{+}$: 787.2883; found 787.2882.

*1,5-Anhydro-2,3,6-tris-O-benzyl-4-O-(3′,4′,5′-tribenzyloxybenzoyl)-d-glucitol* (**19**); Compound **13** (340 mg, 0.78 mmol), compound **16** (530 mg, 1.2 mmol), 2-chloro-1-methylpyridinium iodide (307 mg, 1.2 mmol), DMAP (95 g, 0.78 mmol), TEA (315 μL, 2.3 mmol) in 15 mL of DCM was stirred at rt for 2 d. By the same procedure previously described for the preparation of compound **17**, compound **19** (607 mg, 91%) was obtained as a colorless amorphous oil. ${\left[\alpha \right]}_{D}^{20}$ = −32.0 (c 0.95, ${\mathrm{CHCl}}_{3}$); H1 NMR (${\mathrm{CDCl}}_{3}$, 600 MHz) δ 7.44–7.26 (m, 20H), 7.23–7.15 (m, 7H), 7.11–7.03 (m, 5H), 5.18–5.15 (m, 3H), 5.13–5.07 (m, 4H), 4.76, 4.54 (ABq, *J* = 11.3 Hz, 2H), 4.74, 4.65 (ABq, *J* = 11.7 Hz, 1H), 4.45 (s, 2H), 4.08 (dd, *J* = 11.3, 5.2 Hz, 1H), 3.75–3.71 (m, 1H), 3.65 (t, *J* = 9.1 Hz, 1H), 3.59–3.56 (m, 1H), 3.50 (dd, *J* = 10.8, 2.6 Hz, 1H), 3.45 (dd, *J* = 10.7, 5.8 Hz, 1H), 3.29 (t, *J* = 11.0 Hz, 1H); C13 NMR (${\mathrm{CDCl}}_{3}$, 150 MHz) δ 164.83, 152.41, 142.58, 138.03, 137.58, 137.37, 136.62, 128.57, 128.50, 128.23, 128.14, 128.06, 128.01, 127.94, 127.86, 127.83, 127.58, 127.49, 127.45, 124.67, 109.34, 83.08, 78.18, 78.08, 75.13, 74.94, 73.67, 73.40, 71.24, 69.47, 68.23; HRMS (ESI, *m/z*): ${\left[M+\mathrm{Na}\right]}^{+}$, calcd for ${{[C}_{55}H_{52}O_{9}\mathrm{Na}]}^{+}$: 879.3509; found 879.3511.

*1,5-Anhydro-2,3,4-tris-O-benzyl-6-O-(3′,4′,5′-tribenzyloxybenzoyl)-d-glucitol* (**20**); Compound **11** (0.43 g, 1.0 mmol), compound **16** (0.66 g, 1.5 mmol), 2-chloro-1-methylpyridinium iodide (0.38 g, 1.5 mmol), DMAP (0.18 g, 1.5 mmol), TEA (0.42 mL, 3.0 mmol) in 20 mL of DCM was stirred at rt for 2 d. By the same procedure previously described for the preparation of compound **17**, compound **20** (0.50 g, 58%) was obtained as a colorless amorphous oil. ${\left[\alpha \right]}_{D}^{20}$ = +27.9 (c 1.00, ${\mathrm{CHCl}}_{3}$); H1 NMR (${\mathrm{CDCl}}_{3}$, 600 MHz) δ 7.43–7.18 (m, 32H), 5.14–5.10 (m, 6H), 5.03, 4.88 (ABq, *J* = 10.7 Hz, 2H), 4,85, 4.53 (ABq, *J* = 11.8 Hz, 2H), 4.74, 4.67 (ABq, *J* = 11.7 Hz, 2H), 4.52 (dd, *J* = 12.0, 2.1 Hz, 1H), 4.37 (dd, *J* = 12.0, 4.5 Hz, 1H), 4.03 (dd, *J* = 11.3, 5.2 Hz, 1H), 3.69 (t, *J* = 8.8 Hz, 1H), 3.66–3.62 (m, 1H), 3.52–3.49 (m, 1H), 3.46 (t, *J* = 8.9 Hz, 1H), 3.23 (t, *J* = 10.8 Hz, 1H); C13 NMR (${\mathrm{CDCl}}_{3}$, 150 MHz) δ 165.82, 152.41, 142.46, 138.55, 138.04, 137.69, 137.37, 136.65, 128.59, 128.53, 128.48, 128.46, 128.20, 128.08, 128.01, 127.96, 127.93, 127.85, 127.76, 127.45, 124.91, 109.30, 86.33, 78.48, 77.83, 77.57, 75.73, 75.23, 75.09, 73.29, 71.17, 68.12, 63.86; HRMS (ESI, *m/z*): ${\left[M+\mathrm{Na}\right]}^{+}$, calcd for ${{[C}_{55}H_{52}O_{9}\mathrm{Na}]}^{+}$: 879.3509; found 879.3509.

*1,5-Anhydro-2,3-bis-O-(3′,4′,5′-tribenzyloxybenzoyl)-4,6-O-benzylidene-d-glucitol* (**21**); Compound **1** (380 mg, 1.5 mmol), compound **16** (2.0 g, 4.5 mmol), 2-chloro-1-methylpyridinium iodide (1.2 g, 4.5 mmol), DMAP (0.55 g, 4.5 mmol), TEA (1.3 mL, 9.0 mmol) in 20 mL of DCM was stirred at rt for 2 d. By the same procedure previously described for the preparation of compound **17**, compound **21** (1.6 g, 88%) was obtained as a colorless amorphous oil. ${\left[\alpha \right]}_{D}^{20}$ = +46.4 (c 1.00, ${\mathrm{CHCl}}_{3}$); H1 NMR ( ${\mathrm{CDCl}}_{3}$, 600 MHz) δ 7.44–7.42 (m, 6H), 7.34–7.31 (m, 26H), 7.25–7.22 (m, 4H), 5.80 (t, *J* = 9.5 Hz, 1H), 5.56 (s, 1H), 5.27–5.23 (m, 1H), 5.11–4.93 (m, 12H), 4.45–4.41 (m, 2H), 3.87 (t, *J* = 16.0 Hz, 1H), 3.82 (t, *J* = 17.0 Hz, 1H), 3.65–3.62 (m, 1H), 3.55 (t, *J* = 10.5 Hz, 1H); C13 NMR (${\mathrm{CDCl}}_{3}$, 150 MHz) δ 165.6, 165.4, 152.6, 142.9, 142.8, 137.4, 136.8, 136.5, 129.1, 128.5, 128.4, 128.3, 128.2, 128.1, 128.0, 127.5, 126.2, 109.3, 109.1, 101.6, 78.8, 75.1, 73.0, 71.9, 71.2, 71.2, 71.1, 68.7, 67.7; HRMS (ESI, *m/z*): ${\left[M+\mathrm{Na}\right]}^{+}$, calcd for ${{[C}_{48}H_{44}O_{9}\mathrm{Na}]}^{+}$: 1119.3932; found 1119.3901.

*1,5-Anhydro-2,4-bis-O-(3′,4′,5′-tribenzyloxybenzoyl)-3,6-bis-O-benzyl-d-glucitol* (**22**); Compound **15** (0.30 mg, 0.87 mmol), compound **16** (1.3 g, 3.0 mmol), 2-chloro-1-methylpyridinium iodide (0.77 g, 3.0 mmol), DMAP (0.37 g, 3.0 mmol), TEA (0.83 mL, 6.0 mmol) in 20 mL of DCM was stirred at rt for 2 d. By the same procedure previously described for the preparation of compound **17**, compound **22** (0.93 g, 77%) was obtained as a colorless amorphous oil. ${\left[\alpha \right]}_{D}^{20}$ = +7.87 (c 1.00, ${\mathrm{CHCl}}_{3}$); H1 NMR H1 NMR (${\mathrm{CDCl}}_{3}$, 600 MHz) δ 7.58–7.14 (m, 45H), 7.08–6.95 (m, 5H), 5.32–5.25 (m, 2H), 5.19–5.08 (m, 13H), 4.61–4.45 (m, 4H), 4.29 (dd, *J* = 11.3, 5.5 Hz, 1H), 3.94 (t, *J* = 9.1 Hz, 1H), 3.72–3.68 (m, 1H), 3.58–3.53 (m, 2H), 3.39 (t, *J* = 10.8 Hz, 1H); C13 NMR (${\mathrm{CDCl}}_{3}$, 150 MHz) δ 164.87, 164.71, 152.61, 152.51, 142.88, 142.79, 137.54, 137.52, 137.32, 137.27, 136.56, 136.49, 128.59, 128.57, 128.55, 128.28, 128.24, 128.22, 128.16, 128.10, 128.05, 128.02, 127.85, 127.76, 127.65, 127.53, 127.49, 124.51, 109.43, 80.72, 78.29, 75.16, 74.16, 73.72, 72.12, 71.35, 71.32, 69.44, 67.03; HRMS (ESI, *m/z*): ${\left[M+\mathrm{Na}\right]}^{+}$, calcd for ${{[C}_{76}H_{68}O_{13}\mathrm{Na}]}^{+}$: 1211.4558; found 1211.4554.

*1,5-Anhydro-2,6-bis-O-(3′,4′,5′-tribenzyloxybenzoyl)-3,4-bis-O-benzyl-d-glucitol* (**23**); Compound **10** (0.45 g, 1.3 mmol), compound **16** (1.8 g, 4.0 mmol), 2-chloro-1-methylpyridinium iodide (1.0 g, 4.0 mmol), DMAP (0.49 g, 4.0 mmol), TEA (1.1 mL, 8.0 mmol) in 30 mL of DCM was stirred at rt for 2 d. By the same procedure previously described for the preparation of compound **17**, compound **23** (1.4 g, 92%) was obtained as a colorless amorphous oil. ${\left[\alpha \right]}_{D}^{20}$ = +51.2 (c 1.00, ${\mathrm{CHCl}}_{3}$); H1 NMR (${\mathrm{CDCl}}_{3}$, 600 MHz) δ 7.43–7.18 (m, 44H), 5.27–5.21 (m, 1H), 5.17–5.07 (m, 12H), 4.82, 4.52 (ABq, *J* = 11.0 Hz, 2H), 4.77, 4.70 (ABq, *J* = 11.3 Hz, 2H), 4.56 (m, 1H), 4.42–4.39 (m, 1H), 4.19 (dd, *J* = 11.2, 5.7 Hz, 1H), 3.85–3.82 (m, 1H), 3.60–3.56 (m, 2H), 3.31 (t, *J* = 10.7 Hz, 1H); C13 NMR (${\mathrm{CDCl}}_{3}$, 150 MHz) δ 165.80, 165.05, 152.56, 152.43, 142.76, 142.47, 137.88, 137.49, 137.37, 137.28, 136.66, 136.53, 128.62, 128.60, 128.57, 128.51, 128.39, 128.21, 128.12, 128.06, 128.01, 127.88, 127.80, 127.40, 127.37, 124.80, 124.49, 109.32, 109.25, 84.32, 77.91, 77.42, 75.45, 75.26, 75.13, 75.09, 72.22, 71.26, 71.15, 67.11, 63.52; HRMS (ESI, *m/z*): ${\left[M+\mathrm{Na}\right]}^{+}$, calcd for ${{[C}_{76}H_{68}O_{13}\mathrm{Na}]}^{+}$: 1211.4558; found 1211.4550.

*1,5-Anhydro-2,6-bis-O-benzyl-3,4-bis-O-(3′,4′,5′-tribenzyloxybenzoyl)-d-glucitol* (**24**); Compound **14** (380 mg, 1.1 mmol), compound **16** (1.5 g, 3.3 mmol), 2-chloro-1-methylpyridinium iodide (0.84 g, 3.3 mmol), DMAP (0.91 g, 3.3 mmol), TEA (0.91 mL, 6.6 mmol) in 30 mL of DCM was stirred at rt for 2 d. By the same procedure previously described for the preparation of compound **17**, compound **24** (1.2 g, 92%) was obtained as a colorless amorphous oil. ${\left[\alpha \right]}_{D}^{20}$ = −80.8 (c 1.00, ${\mathrm{CHCl}}_{3}$); H1 NMR (${\mathrm{CDCl}}_{3}$, 600 MHz) δ 7.41–7.14 (m, 44H), 5.58 (t, *J* = 9.5 Hz, 1H), 5.31 (t, *J* = 9.8 Hz, 1H), 5.09–5.00 (m, 12H), 4.54, 4.48 (ABq, *J* = 12.2 Hz, 2H), 4.52, 4.47 (ABq, *J* = 12.0 Hz, 2H), 4.19 (dd, *J* = 11.5, 5.3 Hz, 1H), 3.79 (td, *J* = 9.9, 5.2 Hz, 1H), 3.73 (m, 1H), 3.59 (dd, *J* = 10.7, 2.4 Hz, 1H), 3.51 (dd, *J* = 10.8, 5.3 Hz, 1H), 3.46 (t, *J* = 11.0 Hz, 1H); C13 NMR (${\mathrm{CDCl}}_{3}$, 150 MHz) δ 165.62, 165.18, 152.43, 142.67, 142.53, 137.56, 137.48, 137.43, 137.39, 136.57, 136.56, 128.49, 128.41, 128.38, 128.36, 128.27, 128.16, 127.98, 127.89, 127.87, 127.84, 127.65, 127.55, 124.67, 124.20, 109.15, 77.99, 76.35, 75.22, 75.10, 75.08, 73.70, 72.96, 71.12, 70.07, 69.06, 68.30; HRMS (ESI, *m/z*): ${\left[M+\mathrm{Na}\right]}^{+}$, calcd for ${{[C}_{76}H_{68}O_{13}\mathrm{Na}]}^{+}$: 1211.4558; found 1211.4558.

*1,5-Anhydro-2,4-bis-O-benzyl-3,6-bis-O-(3′,4′,5′-tribenzyloxybenzoyl)-d-glucitol* (**25**); Compound **9** (0.52 mg, 1.5 mmol), compound **16** (2.0 g, 4.5 mmol), 2-chloro-1-methylpyridinium iodide (1.2 g, 4.5 mmol), DMAP (0.55 g, 4.5 mmol), TEA (1.2 mL, 9.0 mmol) in 20 mL of DCM was stirred at rt for 2 d. By the same procedure previously described for the preparation of compound **17**, compound **25** (1.6 g, 91%) was obtained as a colorless amorphous oil. ${\left[\alpha \right]}_{D}^{20}$ = +16.1 (c 1.00, ${\mathrm{CHCl}}_{3}$); H1 NMR (${\mathrm{CDCl}}_{3}$, 600 MHz) δ 7.43–7.24 (m, 34H), 7.18–7.05 (m, 10H), 5.50 (t, *J* = 9.3 Hz, 1H), 5.19–5.08 (m, 12H), 4.56, 4.43 (ABq, *J* = 12.4 Hz, 2H), 4.55 (dd, *J* = 12.0 Hz, 2.0 Hz, 1H), 4.45 (dd, *J* = 12.0, 5.5 Hz, 1H) 4.41 (s, 2H), 4.11 (dd, *J* = 11.3, 5.2 Hz, 1H), 3.67–3.62 (m, 2H), 3.54 (t, *J* = 9.5 Hz, 1H), 3.36 (t, *J* = 11.0 Hz, 1H); C13 NMR (${\mathrm{CDCl}}_{3}$, 150 MHz) δ 165.84, 165.22, 152.49, 142.66, 137.65, 137.31, 136.99, 136.59, 128.56, 128.36, 128.22, 128.16, 128.05, 127.94, 127.83, 127.52, 127.47, 124.96, 124.88, 109.54, 109.51, 78.19, 77.87, 76.42, 75.26, 75.13, 74.68, 72.63, 71.32, 67.97, 63.91; HRMS (ESI, *m/z*): ${\left[M+\mathrm{Na}\right]}^{+}$, calcd for ${{[C}_{76}H_{68}O_{13}\mathrm{Na}]}^{+}$: 1211.4558; found 1211.4557.

*1,5-Anhydro-2,3-bis-O-benzyl-4,6-bis-O-(3′,4′,5′-tribenzyloxybenzoyl)-d-glucitol* (**26**); Compound **5** (0.34 g, 1.0 mmol), compound **16** (1.3 g, 3.0 mmol), 2-chloro-1-methylpyridinium iodide (0.77 g, 3.0 mmol), DMAP (0.37 g, 3.0 mmol), TEA (0.83 mL, 6.0 mmol) in 20 mL of DCM was stirred at rt for 2 d. By the same procedure previously described for the preparation of compound **17**, compound **26** (1.0 g, 87%) was obtained as a colorless amorphous oil. ${\left[\alpha \right]}_{D}^{20}$ = +13.7 (c 1.00, ${\mathrm{CHCl}}_{3}$); H1 NMR (${\mathrm{CDCl}}_{3}$, 600 MHz) δ 7.45–7.21 (m, 39H), 7.12–7.03 (m, 5H), 5.32 (t, *J* = 9.5 Hz, 1H), 5.15–5.08 (m, 8H), 5.03–5.01 (m, 4H), 4.79, 4.58 (ABq, *J* = 11.5, 2H), 4.76, 4.66 (ABq, *J* = 11.5 Hz, 2H), 4.63 (dd, *J* = 12.0, 2.8 Hz, 1H), 4.12 (dd, *J* = 12.2, 5.3 Hz, 1H), 4.07 (dd, *J* = 11.5, 5.0 Hz, 1H), 3.70–3.68 (m, 2H), 3.70 (t, *J* = 8.9 Hz, 1H), 3.30 (t, *J* = 10.8 Hz, 1H); C13 NMR (${\mathrm{CDCl}}_{3}$, 150 MHz) δ 165.80, 164.76, 152.48, 152.44, 142.77, 142.42, 137.98, 137.91, 137.47, 137.37, 136.73, 136.56, 128.53, 128.50, 128.47, 128.20, 128.17, 128.07, 128.03, 128.01, 127.94, 127.84, 127.60, 127.53, 124.75, 124.54, 109.40, 109.14, 82.95, 78.22, 76.56, 75.15, 75.08, 73.47, 71.24, 71.06, 70.96, 68.32, 63.44; HRMS (ESI, *m/z*): ${\left[M+\mathrm{Na}\right]}^{+}$, calcd for ${{[C}_{76}H_{68}O_{13}\mathrm{Na}]}^{+}$: 1211.4558; found 1211.4553.

*1,5-Anhydro-2,3,4-tris-O-(3′,4′,5′-tribenzyloxybenzoyl)-6-O-benzyl-d-glucitol* (**27**); Compound **12** (180 mg, 0.70 mmol), compound **16** (1.4 g, 3.2 mmol), 2-chloro-1-methylpyridinium iodide (0.82 g, 3.2 mmol), DMAP (0.39 g, 3.2 mmol), TEA (0.89 mL, 6.4 mmol) in 20 mL of DCM was stirred at rt for 2 d. By the same procedure previously described for the preparation of compound **17**, compound **27** (0.94 g, 91%) was obtained as a colorless amorphous oil. ${\left[\alpha \right]}_{D}^{20}$ = −4.9 (c 0.65, ${\mathrm{CHCl}}_{3}$); H1 NMR (${\mathrm{CDCl}}_{3}$, 600 MHz) δ 7.43–7.16 (m, 56H), 5.82 (t, *J* = 9.6 Hz, 1H), 5.55 (t, *J* = 10.0 Hz, 1H), 5.29 (td, *J* = 10.0, 5.5 Hz, 1H), 5.13–4.96 (m, 14H), 4.90 (s, 4H), 4.58, 4.53 (ABq, *J* = 12.0, 2H), (dd, *J* = 10.2, 5.6 Hz, 1H), 3.88–3.84 (m, 1H), 3.67 (dd, *J* = 11.0, 2.4 Hz, 1H), 3.61 (dd, *J* = 10.8, 5.3 Hz, 1H), 3.57 (t, *J* = 10.8 Hz, 1H) C13 NMR (${\mathrm{CDCl}}_{3}$, 150 MHz) δ 165.92, 165.17, 165.02, 152.54, 152.50, 142.88, 142.82, 142.71, 137.44, 137.34, 136.49, 136.45, 136.36, 128.55, 128.51, 128.39, 128.32, 128.27, 128.17, 128.15, 128.10, 128.06, 128.02, 127.95, 127.92, 127.89, 127.81, 127.72, 127.56, 127.52, 124.08, 124.03, 109.20, 109.11, 109.02, 78.36, 75.12, 75.09, 75.07, 74.65, 73.77, 71.18, 71.10, 71.02, 70.75, 69.71, 69.00, 67.26; HRMS (ESI, *m/z*): ${\left[M+\mathrm{Na}\right]}^{+}$, calcd for ${{[C}_{97}H_{84}O_{17}\mathrm{Na}]}^{+}$: 1543.5606; found 1543.5601.

*1,5-Anhydro-2,3,6-tris-O-(3′,4′,5′-tribenzyloxybenzoyl)-4-O-benzyl-d-glucitol* (**28**); Compound **8** (0.38 mg, 1.5 mmol), compound **16** (3.0 g, mmol), 2-chloro-1-methylpyridinium iodide (1.8 g, 7.0 mmol), DMAP (0.12 g, 1.0 mmol), TEA (1.9 mL, 14 mmol) in 30 mL of DCM was stirred at rt for 1 d. By the same procedure previously described for the preparation of compound **17**, compound **28** (1.0 g, 45% yield) was obtained as a colorless amorphous oil. ${\left[\alpha \right]}_{D}^{20}$ = +47.2 (c 0.40, ${\mathrm{CHCl}}_{3}$); H1 NMR (${\mathrm{CDCl}}_{3}$, 600 MHz) δ 7.47–7.19 (m, 51H), 7.11–7.05 (m, 5H), 5.75 (t, *J* = 9.1 Hz, 1H), 5.19–5.03 (m, 15H), 5.00, 4.92 (ABq, *J* = 11.7 Hz, 4H), 4.60–4.53 (m, 2H), 4.45 (s, 2H), 4.41 (dd, *J* = 11.3, 5.5 Hz, 1H), 3.75–3.70 (m, 2H), 3.46 (t, *J* = 10.8 Hz, 1H); C13 NMR (${\mathrm{CDCl}}_{3}$, 150 MHz) δ 165.84, 165.51, 165.41, 152.59, 152.54, 142.94, 142.75, 142.65, 137.35, 137.32, 137.27, 136.86, 136.62, 136.51, 136.39, 128.60, 128.54, 128.46, 128.44, 128.38, 128.22, 128.18, 128.14, 128.08, 128.06, 128.01, 127.97, 127.90, 127.61, 127.50, 127.43, 124.81, 124.40, 124.05, 109.54, 109.29, 109.03, 78.11, 76.54, 76.04, 75.14, 75.11, 75.09, 74.96, 71.34, 71.20, 71.06, 70.81, 67.07, 63.58; HRMS (ESI, *m/z*): ${\left[M+\mathrm{Na}\right]}^{+}$, calcd for ${{[C}_{97}H_{84}O_{17}\mathrm{Na}]}^{+}$: 1543.5606; found 1543.5604.

*1,5-Anhydro-2,4,6-tris-O-(3′,4′,5′-tribenzyloxybenzoyl)-3-O-benzyl-d-glucitol* (**29**); Compound **7** (0.33 mg, 1.3 mmol), compound **16** (2.6 g, 6.0 mmol), 2-chloro-1-methylpyridinium iodide (1.5 g, 6.0 mmol), DMAP (0.73 g, 6.0 mmol), TEA (1.7 mL, 12 mmol) in 20 mL of DCM was stirred at rt for 2 d. By the same procedure previously described for the preparation of compound **17**, compound **29** (1.3 g, 65% yield) was obtained as a colorless amorphous oil. ${\left[\alpha \right]}_{D}^{20}$ = +26.7 (c 0.95, ${\mathrm{CHCl}}_{3}$); H1 NMR (${\mathrm{CDCl}}_{3}$, 600 MHz) δ 7.46–7.21 (m, 49H), 7.07–6.96 (m, 5H), 5.48 (t, *J* = 9.5 Hz, 1H), 5.34–5.29 (m, 1H), 5.17–5.07 (m, 14H), 5.04 (s, 4H), 4.68 (dd, *J* = 12.0, 3.1 Hz, 1H), 4.62, 4.54 (ABq, *J* = 11.7 Hz, 2H), 4.30 (dd, *J* = 11.2, 5.7 Hz, 1H), 4.22 (dd, *J* = 12.2, 5.0 Hz, 1H), 3.99 (t, *J* = 9.1 Hz, 1H), 3.90–3.87 (m, 1H), 3.42 (t, *J* = 10.8 Hz, 1H); C13 NMR (${\mathrm{CDCl}}_{3}$, 150 MHz) δ 165.81, 164.83, 164.61, 152.63, 152.55, 152.46, 142.94, 142.49, 137.46, 137.41, 137.33, 137.24, 136.67, 136.50, 136.46, 128.57, 128.53, 128.51, 128.46, 128.21, 128.19, 128.14, 128.10, 128.06, 128.02, 127.95, 127.85, 127.80, 127.62, 127.56, 127.47, 124.64, 124.41, 124.38, 109.47, 109.44, 109.13, 80.62, 76.53, 75.16, 75.08, 74.34, 72.02, 71.35, 71.30, 71.05, 70.98, 67.05, 63.33; HRMS (ESI, *m/z*): ${\left[M+\mathrm{Na}\right]}^{+}$, calcd for ${{[C}_{97}H_{84}O_{17}\mathrm{Na}]}^{+}$: 1543.5606; found 1543.5599.

*1,5-Anhydro-2-O-benzyl-3,4,6-tris-O-(3′,4′,5′-tribenzyloxybenzoyl)-d-glucitol* (**30**); Compound **6** (0.38 g, 1.5 mmol), compound **16** (3.0 g, 6.8 mmol), 2-chloro-1-methylpyridinium iodide (1.7 g, 6.8 mmol), DMAP (0.83 g, 6.8 mmol), TEA (2.0 mL, 14 mmol) in 30 mL of DCM was stirred at rt for 2 days. By the same procedure previously described for the preparation of compound **17**, compound **30** (2.0 g, 89% yield) was obtained as a colorless amorphous oil. ${\left[\alpha \right]}_{D}^{20}$ = −36.2 (c 1.00, ${\mathrm{CHCl}}_{3}$); H1 NMR (${\mathrm{CDCl}}_{3}$, 600 MHz) δ 7.42–7.14 (m, 54H), 5.67 (t, *J* = 9.6 Hz, 1H), 5.41 (t, *J* = 9.8 Hz, 1H), 5.13–4.94 (m, 18H), 4.69 (dd, *J* = 12.2, 2.9 Hz, 1H), 4.59, 4.51 (ABq, *J* = 12.4 Hz, 2H), 4.23 (dd, *J* = 12.4, 5.5 Hz, 1H), 4.19 (dd, *J* = 11.5, 5.3 Hz, 1H) 3.92 (m, 1H), 3.83 (td, *J* = 9.8, 5.3 Hz, 1H), 3.48 (t, *J* = 11.0 Hz, 1H); C13 NMR (${\mathrm{CDCl}}_{3}$, 150 MHz) δ 165.68, 165.65, 165.29, 152.51, 152.48, 152.40, 142.87, 142.66, 142.53, 137.54, 137.43, 137.38, 136.63, 136.46, 128.51, 128.46, 128.43, 128.38, 128.29, 128.15, 128.11, 127.98, 127.92, 127.89, 127.87, 127.80, 127.78, 127.57, 127.52, 124.65, 124.53, 123.97, 109.18, 76.69, 76.16, 75.17, 75.08, 72.95, 71.13, 71.03, 70.20, 68.38, 63.54; HRMS (ESI, *m/z*): ${\left[M+\mathrm{Na}\right]}^{+}$, calcd for ${{[C}_{97}H_{84}O_{17}\mathrm{Na}]}^{+}$: 1543.5606; found 1543.5607.

*1,5-Anhydro-2-O-(3′,4′,5′-trihydroxybenzoyl)-d-glucitol* (**31**) [31]; ${\mathrm{Pd}(\mathrm{OH})}_{2}$ on C (20 wt.%, 20 mg) was added to a solution of **17** (270 mg, 0.35 mmol) in 10 mL of MeOH and 10 mL of THF under the argon. After the replaced argon atmosphere to hydrogen gas, the suspension was stirred at rt for 6 h. The reaction mixture was filtered and concentrated under reduced pressure to obtain purple amorphous oil. The purple amorphous oil was dissolved by 2 mL acetone and filtered through a whatman™ puradisc 0.1 μM TF and concentrated under reduced pressure. In addition, the purple amorphous oil dissolved with 2 mL of MeOH and added acidic resin until becoming a clear solution. After the filtered through the whatman™ puradisc 0.1 μm TF, the solution was concentrated under reduced pressure to give **31** (106 mg, 96%) as pale yellow amorphous oil. ${\left[\alpha \right]}_{D}^{20}$ = +58.5 (c 0.70, MeOH); H1 NMR (${\mathrm{CD}}_{3}\mathrm{OD}$, 600 MHz) δ 7.08 (d, *J* = 5.5 Hz, 2H), 4.87–4.83 (overlap, 1H), 4.08 (dd, *J* = 10.5, 5.5 Hz, 1H), 3.86 (dd, *J* = 11.9, 2.2 Hz, 1H), 3.66–3.63 (m, 2H), 3.36 (t, *J* = 9.5 Hz, 1H), 3.29 (t, *J* = 10.7 Hz, 1H), 3.24 (ddd, *J* = 9.7, 5.9, 2.3 Hz, 1H). C13 NMR (${\mathrm{CD}}_{3}\mathrm{OD}$, 150 MHz) δ 167.80, 146.42, 139.92, 121.14, 110.25, 82.56, 77.06, 73.28, 71.97, 67.84, 62.93; HRMS (${\mathrm{ES}}^{-}$, *m*/*z*): ${[M-H]}^{-}$, calcd for ${{[C}_{13}H_{15}O_{9}]}^{-}$: 315.0716; found 315.0718. Please find H1 NMR, C13 NMR spectra of compounds **31**–**44**, **54**–**58**, **61** and **67** in the Supplementary Materials.

*1,5-Anhydro-3-O-(3′,4′,5′-trihydroxybenzoyl)-d-glucitol* (**32**) [29]; ${\mathrm{Pd}(\mathrm{OH})}_{2}$ on C (20 wt.%, 20 mg) was added to a solution of compound **18** (270 mg, 0.35 mmol) in 10 mL of MeOH and 10 mL of THF under the argon. By the same procedure previously described for the preparation of compound **31**, desired compound **32** (110 mg, quant.) was obtained as a colorless amorphous oil. ${\left[\alpha \right]}_{D}^{20}$ = +24.8 (c 1.0 MeOH); H1 NMR ${\mathrm{CD}}_{3}\mathrm{OD}$, 600 MHz) δ 7.13 (s, 2H), 5.04 (t, *J* = 9.3 Hz, 1H), 3.97 (dd, *J* = 11.3, 5.5 Hz, 1H), 3.85 (dd, *J* = 11.9, 2.2 Hz, 1H), 3.73–3.69 (m, 1H), 3.66 (dd, *J* = 12.0, 5.8 Hz, 1H), 3.51 (t, *J* = 9.5 Hz, 1H), 3.31–3.27 (overlap, 2H). C13 NMR (${\mathrm{CD}}_{3}\mathrm{OD}$, 150 MHz) δ 168.50, 146.39, 139.64, 121.88, 110.34, 82.51, 81.20, 70.95, 69.98, 69.89, 62.75; HRMS (${\mathrm{ES}}^{-}$, *m*/*z*): ${[M-H]}^{-}$, calcd for ${{[C}_{13}H_{15}O_{9}]}^{-}$: 315.0716; found 315.0721.

*1,5-Anhydro-4-O-(3′,4′,5′-trihydroxybenzoyl)-d-glucitol* (**33**) [29]; ${\mathrm{Pd}(\mathrm{OH})}_{2}$ on C (20 wt.%, 20 mg) was added to a solution of compound **19** (280 mg, 0.33 mmol) in 10 mL of MeOH and 10 mL of THF under the argon. By the same procedure previously described for the preparation of compound **31**, desired compound **33** (100 mg, 96%) was obtained as a colorless amorphous oil. ${\left[\alpha \right]}_{D}^{20}$ = −5.7 (c 0.90, MeOH); H1 NMR (${\mathrm{Acetone}-d}_{6},\;$600 MHz) δ 7.14 (s, 2H), 4.88 (t, *J* = 9.3 Hz, 1H), 3.94 (dd, *J* = 11.2, 5.3 Hz, 1H), 3.68 (t, *J* = 8.9 Hz, 1H), 3.64–3.60 (m, 1H), 3.57 (dd, *J* = 12.0, 2.1 Hz, 1H), 3.51–3.44 (m, 2H), 3.25 (t, *J* = 10.8 Hz, 1H); C13 NMR (Acetone-d6, 150 MHz)  δ 166.59, 145.90, 138.86, 121.46, 110.07, 80.56, 77.44, 72.70, 71.38, 70.45, 62.54; HRMS (${\mathrm{ES}}^{-}$, *m*/*z*): ${[M-H]}^{-}$, calcd for ${{[C}_{13}H_{15}O_{9}]}^{-}$: 315.0716; found 315.0718.

*1,5-Anhydro-6-O-(3′,4′,5′-trihydroxybenzoyl)-d-glucitol* (**34**) [31]; ${\mathrm{Pd}(\mathrm{OH})}_{2}$ on C (20 wt.%, 20 mg) was added to a solution of compound **20** (210 mg, 0.25 mmol) in 10 mL of MeOH and 10 mL of THF under the argon. By the same procedure previously described for the preparation of compound **31**, desired compound **55** (74 mg, 96%) was obtained as a colorless amorphous oil. ${\left[\alpha \right]}_{D}^{20}$ = +30.3 (c 1.00, MeOH); H1 NMR (${\mathrm{CD}}_{3}\mathrm{OD}$, 600 MHz) δ 7.09 (s, 2H), 4.54 (dd, *J* = 11.9, 1.9 Hz, 1H), 4.35 (dd, *J* = 12.0, 5.5 Hz, 1H), 3.93 (dd, *J* = 11.2, 5.3 Hz, 1H), 3.51–3.55 (m, 1H), 3.48–3.45 (m, 1H), 3.40 (t, *J* = 8.6 Hz, 1H), 3.37 (t, *J* = 8.6 Hz, 1H), 3.23 (t, *J* = 10.8 Hz, 1H). C13 NMR (${\mathrm{CD}}_{3}\mathrm{OD}$, 150 MHz) δ 168.32, 146.36, 139.71, 121.26, 110.09, 79.91, 79.63, 71.59, 71.20, 70.86, 65.02; HRMS (${\mathrm{ES}}^{-}$, *m*/*z*): ${[M-H]}^{-}$, calcd for ${{[C}_{13}H_{15}O_{9}]}^{-}$: 315.0716; found 315.0718.

*1,5-Anhydro-2,3-bis-O-(3′,4′,5′-trihydroxybenzoyl)-d-glucitol* (**35**) [29]; ${\mathrm{Pd}(\mathrm{OH})}_{2}$ on C (20 wt.%, 50 mg) was added to a solution of compound **21** (210 mg, 0.25 mmol) in 10 mL of MeOH and 10 mL of THF under the argon. By the same procedure previously described for the preparation of compound **31**, desired compound **35** (313 mg, quant.) was obtained as a colorless amorphous oil. ${\left[\alpha \right]}_{D}^{20}$ = +139.7 (c 1.00, MeOH); H1 NMR (${\mathrm{CD}}_{3}\mathrm{OD}$, 600 MHz) δ 7.05 (s, 2H), 6.96 (s, 2H), 5.41 (t, *J* = 9.5 Hz, 1H), 5.08 (td, *J* = 10.1, 5.3 Hz, 1H), 4.20 (dd, *J* = 11.0, 5.5 Hz, 1H), 3.90 (dd, *J* = 11.9, 2.2 Hz, 1H), 3.74–3.69 (m, 2H), 3.45 (t, *J* = 10.8 Hz, 1H), 3.40 (ddd, *J* = 9.6, 5.5, 2.1 Hz, 1H); C13 NMR (${\mathrm{CD}}_{3}\mathrm{OD}$, 150 MHz) δ 168.18, 167.38, 146.38, 146.34, 140.08, 139.85, 121.29, 120.55, 110.30, 110.25, 82.65, 77.91, 71.39, 69.83, 67.81, 62.57; HRMS (${\mathrm{ES}}^{-}$, *m*/*z*): ${[M-H]}^{-}$, calcd for ${{[C}_{20}H_{19}O_{13}]}^{-}$: 467.0826; found 467.0831.

*1,5-Anhydro-2,4-bis-O-(3′,4′,5′-trihydroxybenzoyl)-d-glucitol* (**36**) [29]; ${\mathrm{Pd}(\mathrm{OH})}_{2}$ on C (20 wt.%, 50 mg) was added to a solution of compound **22** (680 mg, 0.57 mmol) in 20 mL of MeOH and 20 mL of THF under the argon. By the same procedure previously described for the preparation of compound **31**, desired compound **36** (267 mg, quant.) was obtained as a colorless amorphous oil. ${\left[\alpha \right]}_{D}^{20}$ = +11.3 (c 0.70, MeOH); H1 NMR (${\mathrm{Acetone}-d}_{6},\;$600 MHz) δ 7.17 (s, 2H), 7.14 (dd, *J* = 9.8, 4.0 Hz, 2H), 5.04 (t, *J* = 9.5 Hz, 1H), 5.01–4.97 (m, 1H), 4.15–4.11 (m, 2H), 3.63–3.54 (m, 3H), 3.41 (t, *J* = 10.8 Hz, 1H); C13 NMR (Acetone-d6, 150 MHz) δ 166.39, 166.21, 146.00, 145.98, 138.99, 138.94, 121.43, 121.38, 110.15, 110.09, 80.72, 74.30, 73.19, 72.73, 67.38, 62.49; HRMS (${\mathrm{ES}}^{-}$, *m*/*z*): ${[M-H]}^{-}$, calcd for ${{[C}_{20}H_{19}O_{13}]}^{-}$: 467.0826; found 467.0827.

*1,5-Anhydro-2,6-bis-O-(3′,4′,5′-trihydroxybenzoyl)-d-glucitol* (**37**) [26]; ${\mathrm{Pd}(\mathrm{OH})}_{2}$ on C (20 wt.%, 50 mg) was added to a solution of compound **23** (440 mg, 0.37 mmol) in 20 mL of MeOH and 20 mL of THF under the argon. By the same procedure previously described for the preparation of compound **31**, desired compound **37** (170 mg, quant.) was obtained as a yellow amorphous oil. ${\left[\alpha \right]}_{D}^{20}$ = +19.4 (c 1.00 in MeOH); H1 NMR (${\mathrm{Acetone}-d}_{6},\;$600 MHz) δ 7.16 (s, 2H), 7.14 (s, 2H), 4.92–4.88 (m, 1H), 4.57 (d, *J* = 10.7 Hz, 1H), 4.41–4.35 (m, 1H), 4.07 (dd, *J* = 10.8, 5.3 Hz, 1H), 3.83–3.78 (m, 1H), 3.61–3.57 (m, 2H), 3.37 (t, *J* = 10.7 Hz, 1H); C13 NMR (Acetone-d6, 150 MHz) δ 166.63, 166.30, 146.02, 145.96, 138.89, 138.77, 121.74, 121.46, 110.07, 109.85, 79.47, 76.57, 72.94, 71.60, 67.45, 64.54; HRMS (${\mathrm{ES}}^{-}$, *m*/*z*): ${[M-H]}^{-}$, calcd for ${{[C}_{20}H_{19}O_{13}]}^{-}$: 467.0826; found 467.0831.

*1,5-Anhydro-3,4-bis-O-(3′,4′,5′-trihydroxybenzoyl)-d-glucitol* (**38**); ${\mathrm{Pd}(\mathrm{OH})}_{2}$ on C (20 wt.%, 50 mg) was added to a solution of compound **24** (710 mg, 0.60 mmol) in 20 mL of MeOH and 20 mL of THF under the argon. By the same procedure previously described for the preparation of compound **31**, desired compound **38** (280 mg, quant.) was obtained as a yellow amorphous oil. ${\left[\alpha \right]}_{D}^{20}$ = −78.2 (c 1.00, MeOH); H1 NMR (${\mathrm{Acetone}-d}_{6},\;$600 MHz) δ 7.06 (s, 2H), 7.04 (s, 2H), 5.40 (t, *J* = 9.5 Hz, 1H), 5.16 (t, *J* = 9.6 Hz, 1H), 4.06 (dd, *J* = 11.3, 5.5 Hz, 1H), 3.98–3.93 (m, 1H), 3.71–3.64 (m, 2H), 3.58 (dd, *J* = 12.5, 5.7 Hz, 1H), 3.45 (t, *J* = 10.8 Hz, 1H); C13 NMR (Acetone-d6, 150 MHz) δ 166.41, 166.12, 145.83, 145.72, 139.04, 138.66, 121.52, 120.78, 110.05, 110.01, 80.29, 78.04, 70.50, 70.17, 69.50, 62.25; HRMS (${\mathrm{ES}}^{-}$, *m*/*z*): ${[M-H]}^{-}$, calcd for ${{[C}_{20}H_{19}O_{13}]}^{-}$: 467.0826; found 467.0829.

*1,5-Anhydro-3,6-bis-O-(3′,4′,5′-trihydroxybenzoyl)-d-glucitol* (**39**) [35]; ${\mathrm{Pd}(\mathrm{OH})}_{2}$ on C (20 wt.%, 50 mg) was added to a solution of compound **25** (800 mg, 0.67 mmol) in 20 mL of MeOH and 20 mL of THF under the argon. By the same procedure previously described for the preparation of compound **31**, desired compound **39** (310 mg, quant.) was obtained as a yellow amorphous oil. ${\left[\alpha \right]}_{D}^{20}$ = +28.7 (c 0.60, MeOH); H1 NMR (${\mathrm{Acetone}-d}_{6},\;$600 MHz) δ 7.17 (s, 2H), 7.16 (s, 2H), 5.11 (t, *J* = 9.1 Hz, 1H), 4.57 (dd, *J* = 11.9, 1.9 Hz, 1H), 4.40 (dd, *J* = 11.9, 5.3 Hz, 1H), 3.99 (dd, *J* = 11.0, 5.5 Hz, 1H), 3.84–3.79 (m, 1H), 3.72 (t, *J* = 9.5 Hz, 1H), 3.65–3.63 (m, 1H), 3.38 (t, *J* = 10.8 Hz, 1H); C13 NMR (Acetone-d6, 150 MHz) δ 166.90, 166.63, 145.97, 145.82, 138.71, 138.52, 122.16, 121.76, 110.10, 109.85, 81.10, 79.61, 70.73, 69.66, 69.46, 64.54; HRMS (${\mathrm{ES}}^{-}$, *m*/*z*): ${[M-H]}^{-}$, calcd for ${{[C}_{20}H_{19}O_{13}]}^{-}$: 467.0826; found 467.0828.

*1,5-Anhydro-4,6-bis-O-(3′,4′,5′-trihydroxybenzoyl)-d-glucitol* (**40**) [35]; ${\mathrm{Pd}(\mathrm{OH})}_{2}$ on C (20 wt.%, 50 mg) was added to a solution of compound **26** (430 mg, 0.36 mmol) in 20 mL of MeOH and 20 mL of THF under the argon. By the same procedure previously described for the preparation of compound **31**, desired compound **40** (160 mg, 95%) was obtained as a yellow amorphous oil. ${\left[\alpha \right]}_{D}^{20}$ = +41.7 (c 0.60, MeOH); H1 NMR (${\mathrm{Acetone}-d}_{6},\;$600 MHz) δ 7.15 (s, 2H), 7.14 (s, 2H), 5.09 (t, *J* = 9.5 Hz, 1H), 4.41 (dd, *J* = 12.0, 2.1 Hz, 1H), 4.13 (dd, *J* = 12.0, 5.8 Hz, 1H), 3.97 (dd, *J* = 11.2, 5.3 Hz, 1H), 3.82–3.79 (m, 1H), 3.75 (t, *J* = 9.1 Hz, 1H), 3.71–3.66 (m, 1H), 3.33 (t, *J* = 10.8 Hz, 1H); C13 NMR (Acetone-d6, 150 MHz) δ 166.47, 166.13, 145.92, 138.90, 138.77, 121.53, 121.38, 110.12, 109.91, 77.56, 77.42, 72.24, 71.28, 70.51, 69.79, 63.98, 55.32; HRMS (${\mathrm{ES}}^{-}$, *m*/*z*): ${[M-H]}^{-}$, calcd for ${{[C}_{20}H_{19}O_{13}]}^{-}$: 467.0826; found 467.0829.

*1,5-Anhydro-2,3,4-tris-O-(3′,4′,5′-trihydroxybenzoyl)-d-glucitol* (**41**) [35]; ${\mathrm{Pd}(\mathrm{OH})}_{2}$ on C (20 wt.%, 50 mg) was added to a solution of compound **27** (520 mg, 0.34 mmol) in 20 mL of MeOH and 20 mL of THF under the argon. By the same procedure previously described for the preparation of compound **31**, desired compound **41** (210 mg, quant.) was obtained as a yellow amorphous oil. ${\left[\alpha \right]}_{D}^{20}$ = −4.8 (c 1.00, MeOH); H1 NMR (${\mathrm{Acetone}-d}_{6},\;$600 MHz) δ 7.07 (d, *J* = 3.8 Hz, 4H), 7.01 (d, *J* = 3.8 Hz, 2H), 5.77 (t, *J* = 9.6 Hz, 1H), 5.36 (t, *J* = 9.8 Hz, 1H), 5.23 (td, *J* = 10.1, 5.4 Hz, 1H), 4.28 (dd, *J* = 11.2, 5.7 Hz, 1H), 3.83–3.80 (m, 1H), 3.72 (dd, *J* = 12.4, 2.1 Hz, 1H), 3.62–3.65 (m, 2H); C13 NMR (Acetone-d6, 150 MHz) δ 166.12, 165.91, 165.89, 145.80, 145.65, 139.00, 138.76, 120.93, 120.70, 120.62, 110.03, 109.96, 109.91, 80.37, 74.46, 70.83, 69.96, 67.27, 61.99; HRMS (${\mathrm{ES}}^{-}$, *m*/*z*): ${[M-H]}^{-}$, calcd for ${{[C}_{27}H_{23}O_{17}]}^{-}$: 619.0935; found 619.0934.

*1,5-Anhydro-2,3,6-tris-O-(3′,4′,5′-trihydroxybenzoyl)-d-glucitol* (**42**) [30]; ${\mathrm{Pd}(\mathrm{OH})}_{2}$ on C (20 wt.%, 50 mg) was added to a solution of compound **28** (410 mg, 0.27 mmol) in 20 mL of MeOH and 20 mL of THF under the argon. By the same procedure previously described for the preparation of compound **31**, desired compound **42** (170 mg, quant.) was obtained as a yellow amorphous oil. ${\left[\alpha \right]}_{D}^{20}$ = +80.9 (c 0.70, MeOH); H1 NMR (${\mathrm{Acetone}-d}_{6},\;$600 MHz) δ 7.19 (s, 2H), 7.10 (s, 2H), 7.04 (s, 2H), 5.50 (t, *J* = 9.5 Hz, 1H), 5.13–5.09 (m, 1H), 4.60 (dd, *J* = 11.9, 1.9 Hz, 1H), 4.47 (dd, *J* = 12.0, 4.8 Hz, 1H), 4.20 (dd, *J* = 11.0, 5.5 Hz, 1H), 3.93 (t, *J* = 9.5 Hz, 1H), 3.79–3.77 (m, 1H), 3.56 (t, *J* = 10.8 Hz, 1H) C13 NMR (Acetone-d6, 150 MHz) δ 166.60, 166.39, 166.03, 146.05, 145.96, 145.87, 139.15, 138.86, 138.81, 121.64, 121.56, 120.75, 110.06, 110.00, 109.89, 79.67, 77.07, 70.84, 69.60, 67.44, 64.23; HRMS (${\mathrm{ES}}^{-}$, *m*/*z*): ${[M-H]}^{-}$, calcd for ${{[C}_{27}H_{23}O_{17}]}^{-}$: 619.0935; found 619.0931.

*1,5-Anhydro-2,4,6-tris-O-(3′,4′,5′-trihydroxybenzoyl)-d-glucitol* (**43**) [29]; ${\mathrm{Pd}(\mathrm{OH})}_{2}$ on C (20 wt.%, 50 mg) was added to a solution of compound **29** (480 mg, 0.32 mmol) in 20 mL of MeOH and 20 mL of THF under the argon. B By the same procedure previously described for the preparation of compound **31**, desired compound **37** (200 mg, quant.) was obtained as a yellow amorphous oil. ${\left[\alpha \right]}_{D}^{20}$ = +36.3 (c 1.00, MeOH); H1 NMR (${\mathrm{Acetone}-d}_{6},\;$600 MHz) δ 7.17 (s, 2H), 7.16 (s, 2H), 7.15 (s, 2H), 5.25 (dd, *J* = 10.0, 9.3 Hz, 1H), 5.07–5.03 (m, 1H), 4.46 (dd, *J* = 12.2, 1.9 Hz, 1H), 4.20–4.16 (m, 3H), 3.95–3.92 (m, 1H), 3.50 (t, *J* = 10.8 Hz, 1H); C13 NMR (Acetone-d6, 150 MHz) δ 166.44, 166.19, 165.95, 145.98, 138.98, 138.83, 121.56, 121.35, 121.31, 110.20, 110.11, 109.96, 77.63, 74.27, 72.96, 72.22, 67.45, 63.76; HRMS (${\mathrm{ES}}^{-}$, *m*/*z*): ${[M-H]}^{-}$, calcd for ${{[C}_{27}H_{23}O_{17}]}^{-}$: 619.0935; found 619.0934.

*1,5-Anhydro-3,4,6-tris-O-(3′,4′,5′-trihydroxybenzoyl)-d-glucitol* (**44**) [35]; ${\mathrm{Pd}(\mathrm{OH})}_{2}$ on C (20 wt.%, 50 mg) was added to a solution of compound **30** (765 mg, 0.50 mmol) in 20 mL of MeOH and 20 mL of THF under the argon. By the same procedure previously described for the preparation of compound **31**, desired compound **37** (310 mg, quant.) was obtained as a colorless amorphous oil. ${\left[\alpha \right]}_{D}^{20}$ = −6.12 (c 0.80, MeOH); H1 NMR (${\mathrm{Acetone}-d}_{6},\;$600 MHz) δ 7.20 (s, 2H), 7.07 (s, 2H), 7.06 (s, 2H), 5.46 (t, *J* = 9.5 Hz, 1H), 5.34 (t, *J* = 9.8 Hz, 1H), 4.47 (dd, *J* = 12.4, 2.1 Hz, 1H), 4.27 (dd, *J* = 12.2, 5.3 Hz, 1H), 4.12 (dd, *J* = 11.3, 5.5 Hz, 1H), 4.04–4.00 (m, 2H), 3.55 (t, *J* = 10.8 Hz, 1H) C13 NMR (Acetone-d6, 150 MHz)  δ 166.39, 166.35, 165.72, 145.83, 145.73, 145.66, 138.98, 138.74, 138.64, 121.44, 121.38, 120.67, 110.05, 109.97, 109.94, 77.86, 77.41, 70.54, 69.73, 69.41, 63.52; HRMS (${\mathrm{ES}}^{-}$, *m*/*z*): ${[M-H]}^{-}$, calcd for ${{[C}_{27}H_{23}O_{17}]}^{-}$: 619.0935; found 619.0941.

*3,4-bis(Benzyloxy)benzoic acid* (**45**) [51]; Methyl 3,4-dihydroxybenzoate (2.5 g, 15 mmol) and ${\;K}_{2}{\mathrm{CO}}_{3}$ (8.2 g, 60 mmol) and KI (2.0 g, 12 mmol) in 100 mL of acetone was stirred at rt. The reaction suspension was slowly added BnCl (4.2 mL, 36 mmol) and refluxed for 7 h. TLC indicated full conversion of the start material, added MeOH and stirred 1 h. The reaction mixture was filtered by celite and filtrate was evaporated under reduced pressure. The residue was purified by recrystallization with hexane, and the mother liquid was purified by C.C (Hex/EtOAc = 100/1–4/1) to afford methyl ester of **45** (total 5.0 g, 97%) as a white solid. Further on, methyl ester of **45** (3.0 g, 9.0 mmol), KOH (5.0 g, 90 mmol) in 90 mL of 1,4-dioxane and 30 mL of MeOH was stirred at 85 °C for 2 h. TLC indicated full conversion of the start material, the reaction mixture was cooled to 0 °C and slowly added 6 M HCl until pH = 1. The precipitating muddy suspension was filtrated, and the white residue was washed by water and MeOH until pH = 7. The white solid was dried *in vacuo*, purified by recrystallizing with MeOH to obtain desired compound **45** (2.4 g, 79%) as colorless needles. m.p. 211 °C; H1 NMR (DMSO-*d*6, 600 MHz) δ 7.56–7.15 (m, 13H), 5.22 (s, 2H), 5.18 (s, 2H); C13 NMR (DMSO-d6, 150 MHz) δ 166.88, 151.94, 147.50, 136.93, 136.62, 128.36, 128.30, 127.82, 127.71, 127.46, 127.34, 123.36, 123.22, 114.43, 112.97, 69.87, 69.73.

*3,5-bis(Benzyloxy)benzoic acid* (**46**) [53]; Methyl 3,5-dihydroxybenzoate (2.5 g, 15 mmol) and ${\;K}_{2}{\mathrm{CO}}_{3}$ (8.2 g, 60 mmol) and KI (2.0 g, 12 mmol) in 100 mL of acetone was stirred at rt. The reaction suspension was slowly added BnCl (4.2 mL, 36 mmol) and refluxed for 9 h. By the same procedure previously described for the preparation of compound **45**, methyl ester of **46** (5.2 g, quant.) was obtained as a white solid. Further on, methyl ester of **46** (3.0 g, 9.0 mmol), KOH (5.0 g, 90 mmol) in 90 mL of 1,4-dioxane and 30 mL of MeOH was stirred at 85 °C for 2 h. By the same procedure previously described for the preparation of compound **45**, desire compound **46** (2.5 g, 81%) was obtained as colorless needles. m.p. 185 °C; H1 NMR (DMSO-d6, 600 MHz) δ 7.46–7.30 (m, 11H), 7.19–7.16 (m, 2H), 6.93–6.93 (m, 1H), 5.15 (s, 4H); C13 NMR (DMSO-d6, 150 MHz) δ 166.80, 159.30, 136.64, 132.80, 128.36, 127.79, 127.58, 107.87, 106.43, 69.38.

*3-(Benzyloxy)benzoic acid* (**47**) [52]; Methyl 3-hydroxybenzoate (2.3 g, 15 mmol) and ${\;K}_{2}{\mathrm{CO}}_{3}$ (4.2 g, 30 mmol) and KI (1.0 g, 6.0 mmol) in 100 mL of acetone was stirred at rt. The reaction suspension was slowly added BnCl (2.1 mL, 18 mmol) and refluxed for 10 h. By the same procedure previously described for the preparation of compound **45**, methyl ester of **47** (3.7 g, 95%) was obtained as a white solid. Further on, methyl ester of **47** (1.9 g, 7.4 mmol), KOH (4.2 g, 74 mmol) in 90 mL of 1,4-dioxane and 30 mL of MeOH was stirred at 85 °C for 2 h. By the same procedure previously described for the preparation of compound procedure described for previously **45** preparation, desire compound **47** (1.4 g, 85%) was obtained as colorless needles. m.p. 136 °C; H1 NMR (600 MHz, DMSO-*d*6) δ 7.57–7.53 (m, 2H), 7.48–7.39 (m, 5H), 7.35–7.32 (m, 1H), 7.28–7.26 (m, 1H), 5.17 (s, 2H); C13-NMR (150 MHz, DMSO-*d*6) δ 167.12, 158.34, 136.84, 132.23, 129.79, 128.50, 127.92, 127.70, 121.82, 119.77, 114.89, 69.35.

*4-(Benzyloxy)benzoic acid* (**48**) [51]; Methyl 4-hydroxybenzoate (2.3 g, 15 mmol) and ${\;K}_{2}{\mathrm{CO}}_{3}$ (4.2 g, 30 mmol) and KI (1.0 g, 6.0 mmol) in 100 mL of acetone was stirred at rt. The reaction suspension was slowly added BnCl (2.1 mL, 18 mmol) and refluxed for 9 h. By the same procedure previously described for the preparation of compound **45**, methyl ester of **48** (3.6 g, quant.) was obtained as a white solid. Further on, methyl ester of **48** (2.3 g, 9.0 mmol), KOH (5.0 g, 90 mmol) in 90 mL of 1,4-dioxane and 30 mL of MeOH was stirred at 85 °C for 2 h. By the same procedure previously described for the preparation of compound procedure described for previously **45** preparation, desired compound **48** (1.4 g, 85%) was obtained as colorless needles. m.p. 192 °C; H1 NMR (600 MHz, DMSO-*d*6) δ 7.95–7.90 (m, 2H), 7.48–7.46 (m, 2H), 7.42–7.40 (m, 2H), 7.36–7.34 (m, 1H), 7.12–7.09 (m, 2H), 5.19 (s, 2H); C13-NMR (150 MHz, DMSO-*d*6) δ 166.90, 161.83, 136.43, 131.27, 128.40, 127.92, 127.73, 123.09, 114.50, 69.35.

*1,5-Anhydro-2,3,4,6-tetrakis-O-(3′,4′,5′-tribenzyloxybenzoyl)-d-glucitol* (**49**) [50]; 1,5-AG (82 mg, 0.50 mmol), compound **16** (1.1 g, 2.4 mmol), 2-chloro-1-methylpyridinium iodide (0.61 g, 2.4 mmol), DMAP (0.29 g, 2.4 mmol), TEA (0.67 mL, 4.8 mmol) in 20 mL of DCM was stirred at rt for 2 d. By the same procedure previously described for the preparation of compound **17**, desire compound **49** (0.86 g, 92%) was obtained as a colorless amorphous oil. ${\left[\alpha \right]}_{D}^{20}$ = +7.0 (c 1.00, ${\mathrm{CHCl}}_{3}$); H1 NMR (600 MHz, CHLOROFORM-D) δ 7.56–7.07 (m, 68H), 5.89 (t, *J* = 9.8 Hz, 1H), 5.63 (t, *J* = 9.8 Hz, 1H), 5.35–5.31 (m, 1H), 5.18–4.94 (m, 22H), 4.88–4.84 (m, 4H), 4.76 (dd, *J* = 12.2, 2.9 Hz, 1H), 4.52 (dd, *J* = 11.3, 5.5 Hz, 1H), 4.30 (dd, *J* = 12.4, 5.2 Hz, 1H), 4.06–4.03 (m, 1H), 3.60 (t, *J* = 10.8 Hz, 1H); C13 NMR (${\mathrm{CDCl}}_{3}$, 150 MHz) δ 165.91, 165.71, 165.14, 165.10, 152.54, 152.43, 143.06, 142.93, 142.79, 142.62, 137.41, 137.31, 136.59, 136.44, 136.35, 136.25, 128.54, 128.45, 128.44, 128.37, 128.27, 128.23, 128.14, 128.11, 128.08, 128.02, 128.00, 127.95, 127.90, 127.87, 127.81, 127.55, 127.52, 124.56, 123.95, 123.79, 109.22, 109.14, 109.03, 75.10, 75.06, 74.53, 71.18, 71.11, 71.05, 70.51, 69.78, 67.31, 63.36 HRMS (ESI, *m/z*): ${\left[M+\mathrm{Na}\right]}^{+}$, calcd for ${{[C}_{118}H_{100}O_{21}\mathrm{Na}]}^{+}$: 1875.6644; found 1875.6653.

*1,5-Anhydro-2,3,4,6-tetrakis-O-(3′,4′-dibenzyloxybenzoyl)-d-glucitol* (**50**); 1,5-AG (0.12 g, 0.7 mmol), compound **45** (1.4 g, 4.2 mmol), 2-chloro-1-methylpyridinium iodide (1.1 g, 4.2 mmol), DMAP (0.52 g, 4.2 mmol), TEA (1.1 mL, 8.0 mmol) in 20 mL of DCM was stirred at rt for 2 d. By the same procedure previously described for the preparation of compound **17**, desired compound **50** (0.61 g, 72%) was obtained as a colorless amorphous oil. ${\left[\alpha \right]}_{D}^{20}$ = +18.8 (c 0.90, ${\mathrm{CHCl}}_{3}$); H1 NMR (${\mathrm{CDCl}}_{3}$, 600 MHz) δ 7.65–7.23 (m, 48H), 6.89–6.86 (m, 2H), 6.82–6.80 (m, 1H), 6.77–6.75 (m, 1H), 5.81 (t, *J* = 9.8 Hz, 1H), 5.56 (t, *J* = 9.6 Hz, 1H), 5.29 (td, *J* = 10.1, 5.5 Hz, 1H), 5.22–5.03 (m, 14H), 4.99 (s, 2H), 4.63 (dd, *J* = 12.4, 2.7 Hz, 1H), 4.43 (dd, *J* = 11.3, 5.5 Hz, 1H), 4.29 (dd, *J* = 12.4, 5.2 Hz, 1H), 3.97–3.94 (m, 1H), 3.53 (t, *J* = 10.8 Hz, 1H); C13 NMR (${\mathrm{CDCl}}_{3}$, 150 MHz) δ 165.83, 165.69, 165.17, 164.91, 153.26, 153.14, 152.96, 148.27, 148.17, 136.87, 136.68, 136.51, 136.38, 128.57, 128.53, 128.46, 128.40, 127.94, 127.86, 127.46, 127.41, 127.09, 127.01, 126.98, 124.36, 124.25, 121.79, 115.37, 115.20, 113.01, 76.91, 73.90, 71.07, 71.04, 70.95, 70.70, 70.64, 70.16, 69.38, 67.29, 63.16; HRMS (ESI, *m/z*): ${\left[M+\mathrm{Na}\right]}^{+}$, calcd for ${{[C}_{62}H_{56}O_{13}\mathrm{Na}]}^{+}$: 1451.4980; found 1451.4977.

*1,5-Anhydro-2,3,4,6-tetrakis-O-(3′,5′-dibenzyloxybenzoyl)-d-glucitol* (**51**); 1,5-AG (0.15 g, 0.9 mmol), compound **46** (1.8 g, 5.4 mmol), 2-chloro-1-methylpyridinium iodide (1.3 g, 5.4 mmol), DMAP (0.66 g, 5.4 mmol), TEA (1.5 mL, 10.8 mmol) in 20 mL of DCM was stirred at rt for 2 d. By the same procedure previously described for the preparation of compound **17**, desired compound **51** (0.69 g, 63%) was obtained as a colorless amorphous oil. ${\left[\alpha \right]}_{D}^{20}$ = +8.3 (c 0.90, ${\mathrm{CHCl}}_{3}$); H1 NMR (${\mathrm{CDCl}}_{3}$, 600 MHz) δ 7.45–7.18 (m, 48H), 6.79–6.76 (m, 2H), 6.72–6.69 (m, 2H), 5.89 (t, *J* = 9.6 Hz, 1H), 5.62 (t, *J* = 9.8 Hz, 1H), 5.37 (td, *J* = 10.1, 5.6 Hz, 1H), 5.06–4.98 (m, 8H), 4.95–4.84 (m, 8H), 4.66 (dd, *J* = 12.2, 2.9 Hz, 1H), 4.49 (dd, *J* = 11.3, 5.5 Hz, 1H), 4.40 (dd, *J* = 12.2, 5.3 Hz, 1H), 4.04–4.01 (m, 1H), 3.58 (t, *J* = 10.8 Hz, 1H); C13 NMR (${\mathrm{CDCl}}_{3}$, 150 MHz) δ 165.84, 165.80, 165.20, 165.06, 159.76, 159.75, 159.70, 136.44, 136.31, 136.26, 136.22, 131.45, 130.91, 130.87, 130.71, 128.60, 128.57, 128.51, 128.09, 128.06, 127.59, 127.57, 108.53, 108.49, 108.41, 108.11, 107.74, 107.70, 107.51, 74.28, 70.41, 70.23, 70.19, 69.73, 67.21, 63.51; HRMS (ESI, *m/z*): ${\left[M+\mathrm{Na}\right]}^{+}$, calcd for ${{[C}_{62}H_{56}O_{13}\mathrm{Na}]}^{+}$: 1451.4980; found 1451.4980.

*1,5-Anhydro-2,3,4,6-tetrakis-O-(3′-benzyloxybenzoyl)-d-glucitol* (**52**); 1,5-AG (0.10 g, 0.6 mmol), compound **47** (0.82 g, 3.6 mmol), 2-chloro-1-methylpyridinium iodide (0.81 g, 3.6 mmol), DMAP (0.44 g, 3.6 mmol), TEA (1.0 mL, 7.2 mmol) in 20 mL of DCM was stirred at rt for 2 d. By the same procedure previously described for the preparation of compound **17**, desire compound **52** (0.56 g, 92%) was obtained as a colorless amorphous oil. ${\left[\alpha \right]}_{D}^{20}$ = +22.7 (c 1.00, ${\mathrm{CHCl}}_{3}$); H1 NMR (${\mathrm{CDCl}}_{3}$, 600 MHz) δ 7.68–7.05 (m, 36H), 5.90 (t, *J* = 9.6 Hz, 1H), 5.65 (t, *J* = 9.8 Hz, 1H), 5.40 (td, *J* = 10.1, 5.6 Hz, 1H), 5.02–5.13 (m, 4H), 5.00 (s, 2H), 4.96 (s, 2H), 4.65 (dd, *J* = 12.0, 2.7 Hz, 1H), 4.48 (dd, *J* = 11.2, 5.7 Hz, 1H), 4.42 (dd, *J* = 12.2, 5.3 Hz, 1H), 4.02–4.05 (m, 1H), 3.59 (t, *J* = 10.8 Hz, 1H); C13 NMR (${\mathrm{CDCl}}_{3}$, 150 MHz) δ 166.03, 165.85, 165.34, 165.11, 158.66, 136.57, 136.37, 130.91, 130.30, 130.12, 129.56, 129.49, 128.61, 128.56, 128.13, 127.62, 127.58, 122.52, 121.12, 120.85, 120.71, 120.53, 115.16, 115.12, 115.00, 114.84, 76.89, 74.07, 70.27, 70.12, 69.50, 67.21, 63.31; HRMS (ESI, *m/z*): ${\left[M+\mathrm{Na}\right]}^{+}$, calcd for ${{[C}_{62}H_{56}O_{13}\mathrm{Na}]}^{+}$: 1027.3306; found 1027.3301.

*1,5-Anhydro-2,3,4,6-tetrakis-O-(4′-benzyloxybenzoyl)-d-glucitol* (**53**); 1,5-AG (0.16 g, 1.0 mmol), compound **48** (1.4 g, 6.1 mmol), 2-chloro-1-methylpyridinium iodide (1.4 g, 6.0 mmol), DMAP (0.73 g, 6.0 mmol), TEA (1.7 mL, 12 mmol) in 30 mL of DCM was stirred at rt for 2 d. By the same procedure previously described for the preparation of compound **17**, desire compound **53** (0.99 g, 98%) was obtained as a colorless amorphous oil. ${\left[\alpha \right]}_{D}^{20}$ = +38.2 (c 0.90, ${\mathrm{CHCl}}_{3}$); H1 NMR (${\mathrm{CDCl}}_{3}$, 600 MHz) δ 8.06–7.96 (m, 2H), 7.92–7.86 (m, 6H), 7.44–7.17 (m, 20H), 6.93–6.78 (m, 8H), 5.89 (t, *J* = 9.5 Hz, 1H), 5.63 (t, *J* = 9.8 Hz, 1H), 5.40 (td, *J* = 10.0, 5.7 Hz, 1H), 5.02–4.85 (m, 8H), 4.61–4.59 (m, 1H), 4.41 (td, *J* = 12.5, 5.4 Hz, 2H), 3.99–3.96 (m, 1H), 3.54 (t, *J* = 10.8 Hz, 1H); C13 NMR (${\mathrm{CDCl}}_{3}$, 150 MHz) δ 165.83, 165.55, 165.15, 164.86, 162.78, 162.62, 162.57, 136.22, 136.07, 131.93, 131.86, 128.63, 128.61, 128.17, 127.46, 127.41, 127.19, 122.30, 121.65, 121.59, 121.46, 114.52, 114.47, 114.43, 114.38, 73.69, 69.96, 69.90, 69.24, 67.23, 63.05; HRMS (ESI, *m/z*): ${\left[M+\mathrm{Na}\right]}^{+}$, calcd for ${{[C}_{62}H_{56}O_{13}\mathrm{Na}]}^{+}$: 1027.3306; found 1027.3304.

*2,3,4,6-tetrakis-O-(3′,4′,5′-Trihydroxybenzoyl)-d-glucitol* (**54**) [50]; ${\mathrm{Pd}(\mathrm{OH})}_{2}$ on C (20 wt.%, 50 mg) was added to a solution of compound **50** (860 mg, 0.46 mmol) in 20 mL of MeOH and 20 mL of THF under the argon. By the same procedure previously described for the preparation of compound **31**, desired compound **54** (321 mg, 90%) was obtained as a yellow amorphous oil. ${\left[\alpha \right]}_{D}^{20}$ = +58.0 (c 1.04, MeOH); H1 NMR (${\mathrm{Acetone}-d}_{6},\;$600 MHz) δ 7.19 (s, 2H), 7.06 (s, 2H) × 2, 6.99 (s, 2H), 5.81 (t, *J* = 9.6 Hz, 1H), 5.50 (t, *J* = 9.8 Hz, 1H), 5.28–5.24 (m, 1H), 4.49 (dd, *J* = 12.4, 2.1 Hz, 1H), 4.33–4.29 (m, 2H), 4.17–4.15 (m, 1H), 3.72 (t, *J* = 10.8 Hz, 1H); C13 NMR (Acetone-d6, 150 MHz) δ 166.36, 166.05, 165.90, 165.63, 146.00, 145.97, 145.92, 145.80, 139.22, 138.97, 138.90, 121.50, 120.90, 120.63, 110.17, 110.08, 110.01, 109.98, 77.57, 74.31, 70.74, 69.61, 67.44, 63.32; HRMS-ESI (*m/z*): ${\left[M+\mathrm{Na}\right]}^{+}$, calcd for ${{[C}_{34}H_{28}O_{21}\mathrm{Na}]}^{+}$: 795.1021; found 795.1019.

*1,5-Anhydro-2,3,4,6-tetrakis-O-(3′,4′-dihydroxybenzoyl)-d-glucitol* (**55**); ${\mathrm{Pd}(\mathrm{OH})}_{2}$ on C (20 wt.%, 50 mg) was added to a solution of compound **50** (365 mg, 0.30 mmol) in 10 mL of MeOH and 10 mL of THF under the argon. By the same procedure previously described for the preparation of compound **31**, desired compound **55** (196 mg, 92%) was obtained as a colorless amorphous oil. ${\left[\alpha \right]}_{D}^{20}$ = +48.3 (c 0.65, MeOH); H1 NMR (${\mathrm{Acetone}-d}_{6},\;$600 MHz); δ 7.56 (d, *J* = 2.1 Hz, 1H), 7.49 (dd, *J* = 8.4, 1.9 Hz, 1H), 7.43–7.33 (m, 6H), 6.90 (d, *J* = 8.2 Hz, 1H), 6.84 (dd, *J* = 11.3, 8.2 Hz, 2H), 6.76 (d, *J* = 8.2 Hz, 1H), 5.85 (t, *J* = 9.6 Hz, 1H), 5.55 (t, *J* = 9.8 Hz, 1H), 5.30 (td, *J* = 10.1, 5.4 Hz, 1H), 4.52 (dd, *J* = 12.0, 2.4 Hz, 1H), 4.37–4.32 (m, 2H), 4.20–4.17 (m, 1H), 3.75 (t, *J* = 10.8 Hz, 1H); C13 NMR (Acetone-d6, 150 MHz) δ 166.22, 166.00, 165.76, 165.56, 151.21, 151.01, 145.55, 145.39, 123.78, 123.71, 123.62, 122.53, 121.96, 121.74, 117.41, 117.29, 117.19, 117.09, 115.74, 115.68, 77.53, 74.40, 70.78, 69.87, 67.48, 63.52; HRMS (${\mathrm{ES}}^{-}$, *m*/*z*): ${[M-H]}^{-}$, calcd for ${{[C}_{34}H_{27}O_{17}]}^{-}$: 707.1248; found 707.1255.

*1,5-Anhydro-2,3,4,6-tetrakis-O-(3,5-dihydroxybenzoyl)-d-glucitol* (**56**); ${\mathrm{Pd}(\mathrm{OH})}_{2}$ on C (20 wt.%, 50 mg) was added to a solution of compound **51** (365 mg, 0.30 mmol) in 20 mL of MeOH and 20 mL of THF under the argon. By the same procedure previously described for the preparation of compound **31**, desired compound **56** (210 mg, quant.) was obtained as a colorless amorphous oil. ${\left[\alpha \right]}_{D}^{20}$ = +39.1 (c 0.50, MeOH); H1 NMR (${\mathrm{Acetone}-d}_{6},\;$600 MHz); δ 7.09 (s, 2H), 6.95 (d, *J* = 1.4 Hz, 4H), 6.89 (d, *J* = 1.7 Hz, 2H), 6.63–6.49 (m, 4H), 5.90 (t, *J* = 9.6 Hz, 1H), 5.59 (t, *J* = 9.6 Hz, 1H), 5.37 (td, *J* = 10.0, 5.6 Hz, 1H), 4.55 (d, *J* = 12.4 Hz, 1H), 4.43 (dd, *J* = 12.2, 4.6 Hz, 1H), 4.37 (dd, *J* = 11.3, 5.5 Hz, 1H), 4.25 (dd, *J* = 9.8, 4.3 Hz, 1H), 3.80 (t, *J* = 10.8 Hz, 1H) C13 NMR (Acetone-d6, 150 MHz) δ 166.33, 166.13, 165.82, 165.67, 159.36, 159.31, 159.24, 132.73, 132.10, 131.91, 129.67, 128.96, 108.81, 108.74, 108.65, 108.39, 108.24, 108.08, 77.25, 74.65, 70.85, 69.79, 67.25, 63.45; HRMS (${\mathrm{ES}}^{-}$, *m*/*z*): ${[M-H]}^{-}$, calcd for ${{[C}_{34}H_{27}O_{17}]}^{-}$: 707.1248; found 707.1255.

*1,5-Anhydro-2,3,4,6-tetrakis-O-(3-hydroxybenzoyl)-d-glucitol* (**57**); ${\mathrm{Pd}(\mathrm{OH})}_{2}$ on C (20 wt.%, 50 mg) was added to a solution of compound **52** (300 mg, 0.30 mmol) in 20 mL of MeOH and 20 mL of THF under the argon. By the same procedure previously described for the preparation of compound **31**, desired compound **57** (181 mg, 94%) was obtained as a colorless amorphous oil. ${\left[\alpha \right]}_{D}^{20}$ = +36.4 (c 0.70, MeOH); H1 NMR $({\mathrm{Acetone}-d}_{6},\;$600 MHz); δ 7.55–6.97 (m, 20H), 5.94 (t, *J* = 9.5 Hz, 1H), 5.65 (t, *J* = 9.6 Hz, 1H), 5.41 (td, *J* = 10.1, 5.4 Hz, 1H), 4.57 (dd, *J* = 12.2, 2.6 Hz, 1H), 4.47 (dd, *J* = 12.4, 4.8 Hz, 1H), 4.39 (dd, *J* = 11.2, 5.7 Hz, 1H), 4.29–4.26 (m, 1H), 3.83 (t, *J* = 10.8 Hz, 1H); C13 NMR (Acetone-d6, 150 MHz) δ 166.38, 166.23, 165.90, 165.82, 158.46, 158.25, 132.14, 131.57, 131.43, 130.49, 130.44, 130.40, 129.72, 129.00, 126.08, 121.43, 121.39, 121.21, 121.08, 117.04, 116.93, 116.88, 116.73, 77.23, 74.83, 70.96, 70.23, 67.33, 63.83; HRMS (${\mathrm{ES}}^{-}$, *m*/*z*): ${[M-H]}^{-}$, calcd for ${{[C}_{34}H_{27}O_{13}]}^{-}$: 643.1452; found 643.1459.

*1,5-Anhydro-2,3,4,6-tetrakis-O-(4-hydroxybenzoyl)-d-glucitol* (**58**); ${\mathrm{Pd}(\mathrm{OH})}_{2}$ on C (20 wt.%, 50 mg) was added to a solution of compound **54** (288 mg, 0.29 mmol) in 15 mL of MeOH and 15 mL of THF under the argon. By the same procedure previously described for the preparation of compound **31**, desired compound **58** (190 mg, quant.) was obtained as a colorless amorphous oil. ${\left[\alpha \right]}_{D}^{20}$ = +45.3 (c 0.50, MeOH); H1 NMR (${\mathrm{Acetone}-d}_{6},\;$600 MHz); δ 7.93–7.76 (m, 8H), 6.92–6.77 (m, 8H), 5.89 (t, *J* = 9.6 Hz, 1H), 5.61 (t, *J* = 9.8 Hz, 1H), 5.34 (td, *J* = 10.0, 5.3 Hz, 1H), 4.55 (dd, *J* = 12.4, 2.7 Hz, 1H), 4.41–4.35 (m, 2H), 4.23–4.20 (m, 1H), 3.77 (t, *J* = 10.8 Hz, 1H); C13 NMR (Acetone-d6, 150 MHz)  δ 166.17, 165.99, 165.69, 165.54, 163.17, 163.13, 162.88, 162.82, 132.75, 132.68, 132.61, 132.58, 121.99, 121.44, 121.21, 121.19, 116.09, 116.05, 116.00, 115.94, 77.45, 74.47, 70.76, 70.05, 67.50, 63.65; HRMS (${\mathrm{ES}}^{-}$, *m*/*z*): ${[M-H]}^{-}$, calcd for ${{[C}_{34}H_{27}O_{13}]}^{-}$: 643.1452; found 643.1459.

*Benzyl 2,3,4,6-tetrakis-O-(3′,4′,5′-tribenzyloxybenzoyl)-α,β-d-glucopyranoside* (**60**); Compound **59** [43] (211 mg, 0.78 mmol), EDC·HCl (1.2 g, 6.3 mmol), compound **16** (2.06 g, 4.7 mmol), DMAP (47.7 mg, 0.39 mmol) in 30 mL of DCM was stirred at rt for overnight. After the addition 20 mL of water, the reaction mixture was extracted with DCM (20 mL). The combined organic layer was washed with water (30 mL × 4) and brine, dried over ${\;\mathrm{Na}}_{2}{\mathrm{SO}}_{4}$, filtered and concentrated under reduced pressure. The crude was purified by C.C (${\mathrm{CHCl}}_{3}$/MeOH = 200/1) to obtain desired compound **60** (791 mg, 52%) as a colorless amorphous oil. ${\left[\alpha \right]}_{D}^{20}$ = +18.1 (c 1.00, ${\mathrm{CHCl}}_{3}$); H1 NMR (${\mathrm{CDCl}}_{3}$, 600 MHz) (assigned for the major anomer; α) δ 7.42–7.16 (m, 80H), 6.21 (t, *J* = 10.0, 1H), 5.69 (t, *J* = 10.0 Hz, 1H), 5.45 (d, *J* = 3.8 Hz, 1H), 5.12–4.95 (m, 16H), 4.73 (dd, *J* = 3.1, 12.4 Hz, 1H), 4.54–4.51 (m, 1H), 4.73 (dd, *J* = 3.1, 12.4 Hz, 1H); C13 NMR (${\mathrm{CDCl}}_{3}$, 150 MHz) (assigned for the major anomer; α) δ 165.68, 165.16, 152.55, 152.52, 152.49, 143.00, 142.86, 142.69, 142.64, 137.41, 137.36, 137.32, 136.56, 136.53, 136.48, 136.40, 136.29, 128.54, 128.46, 128.38, 128.29, 128.24, 128.16, 128.12, 128.09, 128.05, 127.98, 127.94, 127.92, 127.88, 127.81, 127.58, 127.54, 127.52, 124.64, 124.15, 124.02, 123.85, 109.23, 109.16, 109.01, 95.27, 75.11, 75.09, 75.06, 72.17, 71.18, 71.15, 71.05, 70.16, 69.96, 67.96, 63.17. HRMS (ESI, *m/z*): ${\left[M+\mathrm{Na}\right]}^{+}$, calcd for ${{[C}_{125}H_{106}O_{22}\mathrm{Na}]}^{+}$: 1981.7073; found 1981.7067.

*2,3,4,6-tetrakis-O-(3′,4′,5′-Trihydroxybenzoyl)-α,β-d-glucopyranose* (**61**) [55]; ${\mathrm{Pd}(\mathrm{OH})}_{2}$ on C (20 wt.%, 20 mg) was added to a solution of compound **54** (100 mg, 0.51 mmol) in 10 mL of MeOH and 10 mL of THF under the argon. By the same procedure previously described for the preparation of compound **31**, desire compound **58** (34 mg, 85%) was obtained as a colorless amorphous oil. ${\left[\alpha \right]}_{D}^{20}$ = +54.4 (c 0.30, MeOH); H1 NMR (${\mathrm{CDCl}}_{3}$, 600 MHz) δ [α-form] 7.20 (s, 2H), 7.09 (s, 2H), 7.07 (s, 2H), 7.00 (s, 2H), 6.11 (t, *J* = 9.6 Hz, 1H), 5.62 (d, *J* = 3.4 Hz, 1H), 5.56 (t, *J* = 10.2 Hz, 1H), 5.16 (dd, *J* = 10.3, 3.5 Hz, 1H), 4.47 (dd, *J* = 2.4, 11.4 Hz, 1H), 4.35 (dd, *J* = 12.6, 4.8 Hz, 1H), 4.32–4.29 (m, 1H); [β-form] 7.19 (s, 2H), 7.08 (s, 2H), 7.05 (s, 2H), 6.95 (s, 2H), 5.80 (t, *J* = 9.6 Hz, 1H), 5.51 (t, *J* = 9.6 Hz, 1H), 5.29 (t, *J* = 9.0 Hz, 1H), 5.24 (d, *J* = 9.0 Hz, 1H), 4.50 (d, *J* = 1.7, 11.4 Hz, 1H), 4.31 (m, 2H); C13 NMR (Acetone-d6, 150 MHz) (assigned for the major anomer; α) δ 166.48, 166.22, 165.99, 165.73, 146.04, 145.99, 145.84, 139.27, 139.24, 139.00, 138.93, 121.61, 121.02, 120.78, 120.67, 110.21, 110.17, 110.06, 110.01, 91.04, 73.02, 70.70, 69.88, 68.55, 63.27; HRMS (ESI, *m/z*): ${\left[M+\mathrm{Na}\right]}^{+}$, calcd for ${{[C}_{62}H_{56}O_{13}\mathrm{Na}]}^{+}$: 811.0970; found 811.0970.

*1,5-Anhydro-2,3-bis-O-(3′,4′,5′-tribenzyloxybenzoyl)-d-glucitol* (**62**); Compound **21** (3.5 g, 3.2 mmol), iodine (0.71 g, 5.6 mmol) in 100 mL of DCM and 50 mL of MeOH was stirred at 70 °C for 7 days. The reaction mixture was washed with sodium thiosulfate solution and brine. The crude product was purified by C.C (DCM/MeOH = 4/1) to obtain desired compound **62** (3.0 g, 93%) as a white solid. ${\left[\alpha \right]}_{D}^{20}$ = +80.3 (c 1.06, ${\mathrm{CHCl}}_{3}$); H1 NMR (${\mathrm{CDCl}}_{3}$, 600 MHz) δ 7.38–7.21 (m, 32H), 5.34 (t, *J* = 9.0 Hz, 1H), 5.28–5.26 (m, 1H), 5.07–4.97 (m, 12H), 4.35–4.32 (m, 1H), 4.00–3.99 (m, 1H), 3.91–3.88 (m, 2H), 3.51–3.48 (m, 2H), 3.07 (d, *J* = 4.0 Hz, 1H), 2.01 (t, *J* = 6.0 Hz, 1H); C13 NMR (${\mathrm{CDCl}}_{3}$, 150 MHz) δ 167.2, 165.3, 152.6, 143.1, 142.9, 137.3, 136.5, 136.4, 128.5, 128.4, 128.2, 128.1, 128.0, 127.7, 127.5, 109.3, 109.1, 80.4, 78.5, 75.1, 71.1, 67.0, 69.8, 66.9, 62.4; HRMS-ESI (*m/z*): ${\left[M+\mathrm{Na}\right]}^{+}$, calcd for ${{[C}_{62}H_{56}O_{13}\mathrm{Na}]}^{+}$: 1031.3612; found 1031.3619.

*1,5-Anhydro-2,3-bis-O-(3′,4′,5′-tribenzyloxybenzoyl)-4,6-bis-O-(3′,5′-dimetoxymetoxy-4′-benzyloxybenzoyl)-d-glucitol* (**64**); Compound **62** (2.5 g, 2.5 mmol), **63** [46] (2.5 g, 6.2 mmol), EDC·HCl (1.4 g, 7.2 mmol), DMAP (0.15 g, 1.2 mmol) in 15 mL of DCM was stirred at rt for 18 h. After the addition 30 mL of water, the reaction mixture was extracted with EtOAc (3 × 50 mL). The combined organic layer was washed with brine, dried over ${\;\mathrm{Na}}_{2}{\mathrm{SO}}_{4}$, filtered and concentrated under reduced pressure. The crude was purified by C.C (${\mathrm{CHCl}}_{3}$/acetone = 100/1) to obtain **64** (2.3 g, 55% yield) as a colorless amorphous oil. ${\left[\alpha \right]}_{D}^{20}$ = +24.7 (c 2.15 in ${\mathrm{CHCl}}_{3}$); H1 NMR (${\mathrm{CDCl}}_{3}$, 600 MHz) δ 7.58 (s, 2H), 7.46–7.25 (m, 50H), 5.85 (t, *J* = 10Hz, 1H), 5.61 (t, *J* = 10.0Hz, 1H), 5.28–5.26 (m, 1H), 5.22–4.93 (m, 24H), 4.73–4.71 (m, 1H), 4.50–4.47 (m, 1H), 4.35–4.32 (m, 1H), 4.06–4.03 (m, 1H), 3.56 (t, *J* = 11.0 Hz, 1H), 3.48 (s, 6H), 3.42(s, 6H); C13 NMR (${\mathrm{CDCl}}_{3}$, 150 MHz) δ 165.7, 165.5, 165.1, 164.8, 152.5, 150.9, 150.8, 143.7, 143.4, 142.8, 142.7, 137.4, 137.3, 137.2, 136.5, 136.4, 132.4, 130.8, 128.7, 128.5, 128.4, 128.3, 128.2, 128.1, 128.0, 127.9, 127.8, 127.6, 127.5, 125.1, 124.2, 124.1, 124.0, 112.4, 112.3, 109.1, 109.0, 95.4, 75.2, 75.1, 75.0, 74.4, 71.1, 71.0, 70.6, 69.3, 68.1, 67.1, 63.3, 56.4; HRMS-ESI (*m/z*): ${\left[M+\mathrm{Na}\right]}^{+}$, calcd for ${{[C}_{98}H_{92}O_{25}\mathrm{Na}]}^{+}$: 1691.5824; found 1691.5825.

*1,5-Anhydro-2,3-bis-O-(3′,4′,5′-tribenzyloxybenzoyl)-4,6-bis-O-(3′,5′-dihydroxy-4′-benzyloxybenzoyl)-d-glucitol* (**65**); Compound **64** (2.0 g, 1.2 mmol) in 8 mL of THF solution was added 8.8 mL of 2-propanol and 0.2 mL of conc. HCl solution, and the mixture was stirred at 60 °C for 6 h. After the addition 3 mL of ${\mathrm{NaHCO}}_{3}$, the mixture was extracted with EtOAc (3 × 100 mL). The combined organic layer was washed with brine, dried over ${\;\mathrm{Na}}_{2}{\mathrm{SO}}_{4}$, filtered and concentrated under reduced pressure. The crude was purified by C.C (${\mathrm{CHCl}}_{3}$/MeOH = 1000/1–500/1) to obtain **65** (1.7 g, 93% yield) as a white amorphous oil. ${\left[\alpha \right]}_{D}^{20}$ = +26.8 (c 2.36, ${\mathrm{CHCl}}_{3}$); H1 NMR (${\mathrm{CDCl}}_{3}$, 600 MHz) δ 7.43–7.14 (m, 48H), 6.00 (s, 4H), 5.82 (t, *J* = 9.5 Hz, 1H), 5.59 (t, *J* = 9.5 Hz, 1H), 5.37–5.30 (m, 1H), 5.10–4.82 (m, 16H), 4.70–4.68 (m, 1H), 4.54–4.52 (m, 1H), 4.45–4.42 (m, 1H), 3.97–3.95 (m, 1H), 3.57 (t, *J* = 10.5 Hz, 1H); C13 NMR (${\mathrm{CDCl}}_{3}$, 150 MHz) δ 166.2, 165.8, 165.5, 165.2, 152.5, 149.0, 148.9, 142.8, 142.7, 138.2, 137.8, 137.3, 137.2, 136.6, 136.4, 136.2,128.8, 128.7, 128.5, 128.4, 128.3, 128.1, 128.0, 127.9, 127.7, 127.5, 124.5, 123.9, 123.8, 109.9, 109.8, 109.1, 108.9, 77.6, 75.2, 75.1, 75.0, 74.3, 71.1, 71.0, 70.7, 69.7, 68.6, 67.3, 62.6; HRMS-ESI (*m/z*): ${\left[M+\mathrm{Na}\right]}^{+}$, calcd for ${{[C}_{90}H_{76}O_{21}\mathrm{Na}]}^{+}$: 1515.4775; found 1515.4777.

*Benzyl protected 1,5-AG-tellimagrandin analog* (**66**); To a solution of ${\mathrm{CuCl}}_{2}$ (135 mg, 1.0 mmol) in 10 mL of MeOH was added *n*-butylamine (400 μL, 4.0 mmol). After the stirred at rt for 1.5 h, the mixture was added to solution of compound **65** (500 mg, 0.34 mmol) in 20 mL of 1,2-dichloroethane (DCE) and stirred at rt for 30 min. The reaction mixture was diluted with 50 mL of diethyl ether, and 50 mL of 5M aq. HCl and 50 mL of diethyl ether were added. The separating organic layer washed with water, ${\mathrm{NaHCO}}_{3}$ and brine. The organic layer was dried over ${\mathrm{Na}}_{2}{\mathrm{SO}}_{4}$, filtered and concentrated under reduced pressure. The crude was purified by C.C (DCM/MeOH = 250/1) and HPLC (column, FNED01048, 250 × 20 mm, SG80–5 μm, eluant DCM/MeOH = 200/1) to afford **66** (237 mg, 48%) as a peal yellow amorphous oil. ${\left[\alpha \right]}_{D}^{20}$ = +69.5 (c 1.43, ${\mathrm{CHCl}}_{3}$); H1 NMR (${\mathrm{CDCl}}_{3}$, 600 MHz) δ 7.43–7.23 (m, 50H), 6.75 (s, 1H), 6.67 (s, 1H), 5.67 (t, *J* = 10.0 Hz, 1H), 5.37–5.34 (m, 1H), 5.29 (t, *J* = 10.0 Hz, 1H), 5.25–5.24 (m, 1H), 5.13–4.85 (m, 16H), 4.47–4.45 (m, 1H), 3.98 (d, *J* = 12.5 Hz, 1H), 3.96–3.94 (m. 1H), 3.46 (t, *J* = 11.0 Hz, 1H); C13 NMR (CDCl3, 150 MHz) δ 167.1, 166.6, 165.8, 165.2, 152.5, 149.0, 147.0, 142.8, 137.4, 137.3, 136.5, 136.4, 136.3, 136.2, 135.7, 135.5, 130.3, 129.6, 129.0, 128.9, 128.6, 128.5, 128.4, 128.3, 128.1, 128.0, 127.9, 127.8, 127.6, 127.5, 123.9, 113.7, 113.2, 109.2, 108.4, 107.9, 75.6, 75.1, 75.0, 74.4, 71.1, 71.0, 70.8, 70.1, 67.7, 63.6; HRMS-ESI (*m/z*): ${\left[M+\mathrm{Na}\right]}^{+}$, calcd for ${{[C}_{90}H_{74}O_{21}\mathrm{Na}]}^{+}:\;1513.4619$; found 1513.4620.

*1,5-Anhydro-2,3-bis-O-(3′,4′,5′-trihydroxybenzoyl)-4,6-O-hexahydroxydiphenyl-d-glucitol* (**67**); ${\mathrm{Pd}(\mathrm{OH})}_{2}$ on C (20 wt.%, 20 mg) was added to a solution of compound **66** (60 mg, 0.40 mmol) in 2 mL of MeOH and 2 mL of THF under the argon. By the same procedure previously described for the preparation of compound procedure described for previously **31** preparation, desired compound **67** (32 mg, quant.) was obtained as a peal yellow amorphous oil. ${\left[\alpha \right]}_{D}^{20}$ = +113.3 (c 0.29, MeOH); H1 NMR (${\mathrm{CD}}_{3}\mathrm{OD},\;$600 MHz) δ 7.00 (s, 2H), 6.95 (s, 2H), 6.60 (s, 1H), 6.40 (s, 1H), 5.61 (t, *J* = 9.5 Hz, 1H), 5.27–5.19 (m, 2H), 5.03 (t, *J* = 10.0 Hz, 1H), 4.25–4.22 (m, 1H), 4.08–4.07 (m, 1H), 3.80 (t, *J* = 13.0 Hz, 1H), 3.54 (t, *J* = 11.0 Hz, 1H); C13 NMR (${\mathrm{CD}}_{3}\mathrm{OD},$ 150 MHz); 171.1, 170.7, 168.8, 168.3, 150.6, 147.6, 147.1, 144.6, 143.5, 141.8, 139.7, 126.8, 126.7, 126.3, 120.7, 120.3, 120.1, 119.4, 111.1, 110.8, 108.3, 107.7, 78.7, 78.5, 76.2, 72.1, 72.0, 71.8, 71.4, 71.3, 69.4, 68.7, 64.7; HRMS (ESI, *m/z*): ${\left[M+\mathrm{Na}\right]}^{+}$, calcd for ${{[C}_{34}H_{26}O_{21}\mathrm{Na}]}^{+}$: 793.0864; found 793.0865.

## Figures and Tables

**Table 1:** α-Glucosidase inhibitory and antioxidant activities.

Compounds	α-Glucosidase	Antioxidant	
	IC_50_ ${(\mu M)\;}^{a}$	EC_50_ ${\left(\mu M\right)}^{\;b}$	${\mathrm{Trolox}-\mathrm{eq}\;}^{b}$
Mono-			
2-galloyl (Ginnalin C) [31] **31**	95.1 ± 0.22	19.40 ± 1.02	2.04 ± 0.08
3-galloyl (Maplexin A) [29] **32**	137.9 ± 1.50	20.50 ± 2.05	2.38 ± 0.24
4-galloyl (Maplexin B) [29] **33**	143.9 ± 0.52	20.80 ± 1.54	2.09 ± 0.19
6-galloyl (Ginnalin B) [31] **34**	127.1 ± 0.88	22.90 ± 1.61	2.13 ± 0.12
Di-			
2,3-galloyl (Maplexin C) [29] **35**	20.50 ± 0.50	11.30 ± 0.98	4.29 ± 0.23
2,4-galloyl (Maplexin D) [29] **36**	48.30 ± 1.25	14.30 ± 0.29	3.36 ± 0.13
2,6-galloyl (Acertannin) [25] **37**	35.60 ± 2.58	15.20 ± 0.53	3.32 ± 0.37
3,4-galloyl **38** ${}^{c}$	91.40 ± 6.56	14.80 ± 0.56	3.55 ± 0.42
3,6-galloyl **39**	55.00 ± 8.84	11.90 ± 0.71	3.67 ± 0.19
4,6-galloyl **40**	26.60 ± 2.37	13.00 ± 1.12	3.78 ± 0.33
Tri-			
2,3,4-galloyl **41**	6.72 ± 0.21	7.94 ± 0.39	5.59 ± 0.26
2,3,6-galloyl (Maplexin F) [30] **42**	5.34 ± 0.55	8.09 ± 0.41	5.75 ± 0.18
2,4,6-galloyl (Maplexin E) [29] **43**	9.34 ± 0.99	10.30 ± 0.76	4.31 ± 0.35
3,4,6-galloyl **44**	12.60 ± 0.61	8.48 ± 0.21	5.36 ± 0.20
Tetra-			
2,3,4,6-galloyl (Maplexin J) [32] **54**	2.56 ± 0.10	6.61 ± 0.68	6.77 ± 0.63
3′,4′-dihydroxybenzoyl **55** ${}^{c}$	3.28 ± 0.16	6.99 ± 0.11	6.54 ± 0.15
3′,5′-dihydroxybenzoyl **56** ${}^{c}$	9.34 ± 0.02	70<	n.d ${}^{e}$
3′-hydroxybenzoyl **57** ${}^{c}$	${-}^{d}$	78<	n.d ${}^{e}$
4′-hydroxybenzoyl **58** ${}^{c}$	${-}^{d}$	78<	n.d ${}^{e}$
1-OH-2,3,4,6-galloyl **61**	1.68 ± 0.21	5.60 ± 0.28	8.33 ± 0.49
2,3-galloyl-4,6-HHDP **67** ${}^{c}$	3.22 ± 0.51	6.79 ± 0.21	6.44 ± 0.24
Tellimagrandin I	3.37 ± 0.04	6.31 ± 0.27	7.04 ± 0.37
Methyl gallate	90.50 ± 6.97	19.60 ± 0.11	2.42 ± 0.27
Methyl 3,4-dihydroxybenzoate	300<<	21.10 ± 1.08	2.20 ± 0.05
Methyl 3,5-dihydroxybenzoate	300<<	n.d ${}^{e}$	n.d ${}^{e}$
Methyl 3-hydroxybenzoate	660<<	n.d ${}^{e}$	n.d ${}^{e}$
Methyl 4-hydroxybenzoate	660<<	n.d ${}^{e}$	n.d ${}^{e}$
Acarbose	0.11 ± 0.02	-	-
Trolox	-	49.40 ± 3.87	-

${}^{a}$ IC_50_ data represents mean ± S.D. of n = 2. ${}^{b}$ EC_50_ and trolox-eq data represents mean ± S.D. of n = 3. ${}^{c}$ Novel compound. ${}^{d}$ Did not dissolve in water. ${}^{e}$ n.d.: not detected.

## Supplementary Materials

The following are available online H1 NMR, C13 NMR spectra of compounds **31**–**44**, **54**–**58**, **61** and **67**.
