# Supplementary material for: Synthesis and Comparative Structure–Activity Study of Carbohydrate-Based Phenolic Compounds as α-Glucosidase Inhibitors and Antioxidants
Source: Molecules. 2019 Nov 27;24(23):4340. doi: 10.3390/molecules24234340 (PMC6930660; doi:10.3390/molecules24234340)

Supporting Information

Synthesis and Comparative Structure-Activity Study of Carbohydrate-Based Phenolic Compounds as α-Glucosidase Inhibitors and Antioxidants

Shota Machida, Saki Mukai, Rina Kono, Megumi Hunato, Hiroaki Saito and Taketo Uchiyama *

School of Pharmacy, Nihon University, 7-7-1 Narashinodai, Funabashi, Chiba 274-8555, Japan
*Correspondence: taketo.uchiyama@nihon-u.ac.jp; Tel.: +81-047-465-2111 (F.L.)

Contents

^{1}H and ^{13}C NMR spectra of compound **31 – 44**, **54**, – **58**, **61**, and **67**.

^{1}H NMR of compound **31** in CD_3OD

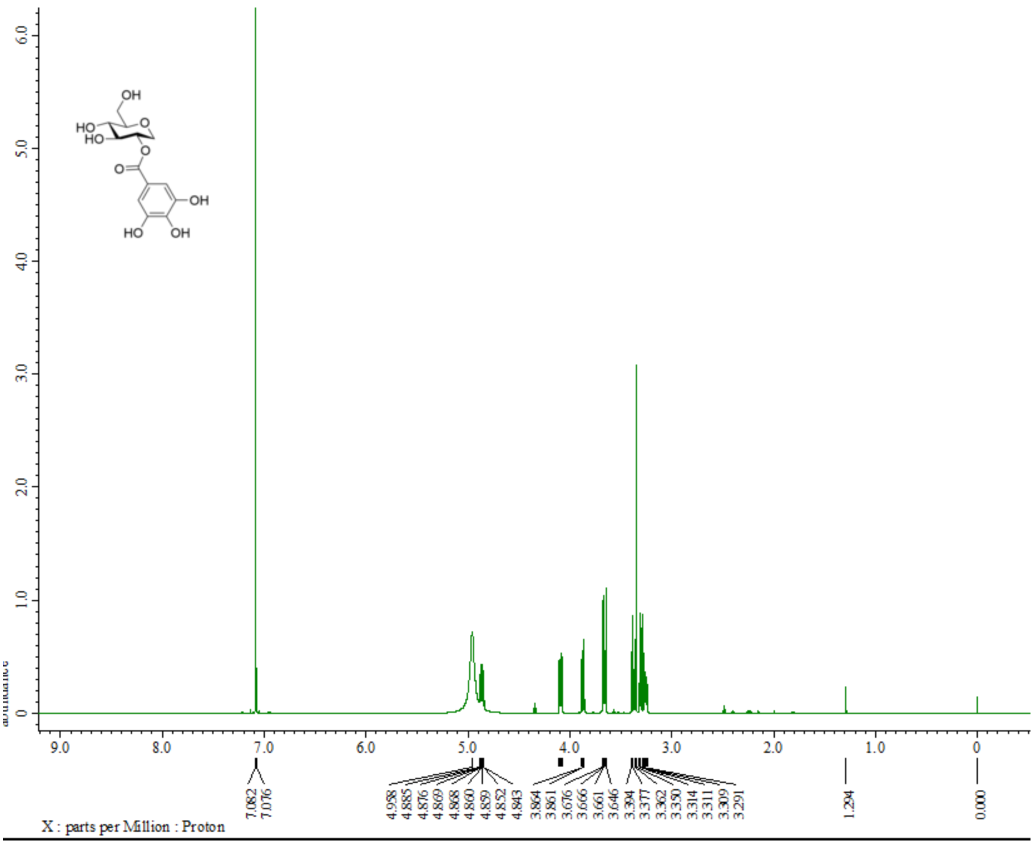

^{13}C NMR of compound **31** in CD_3OD

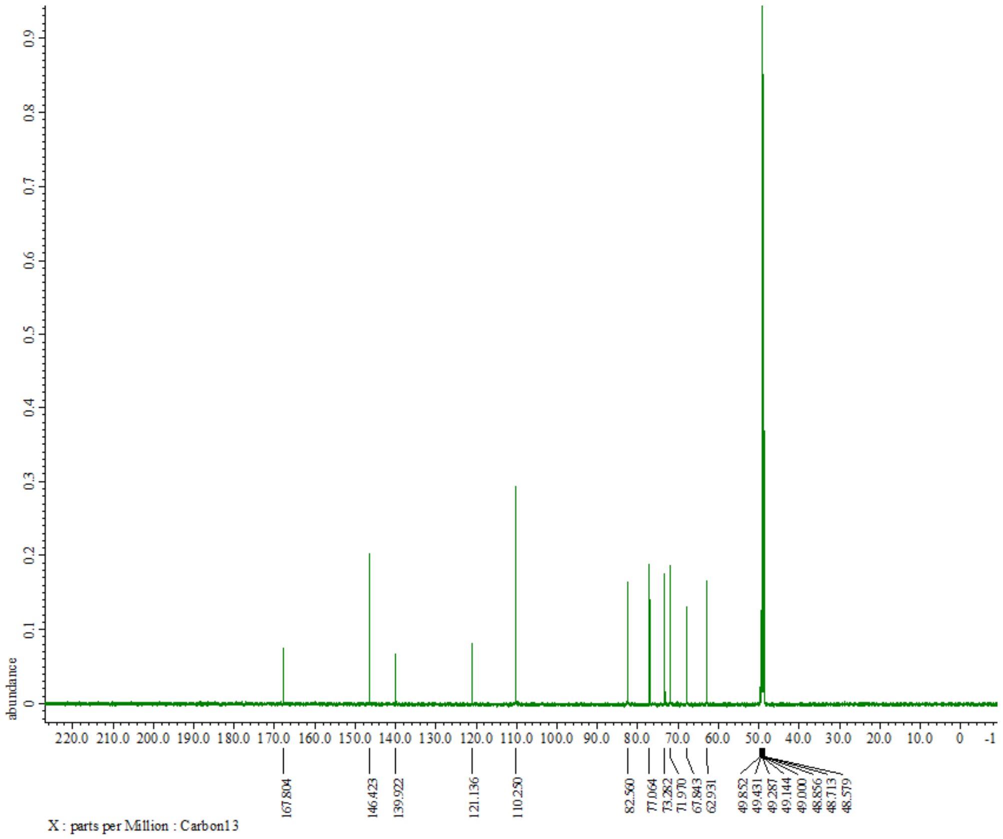

^{1}H NMR of compound **32** in CD_3OD

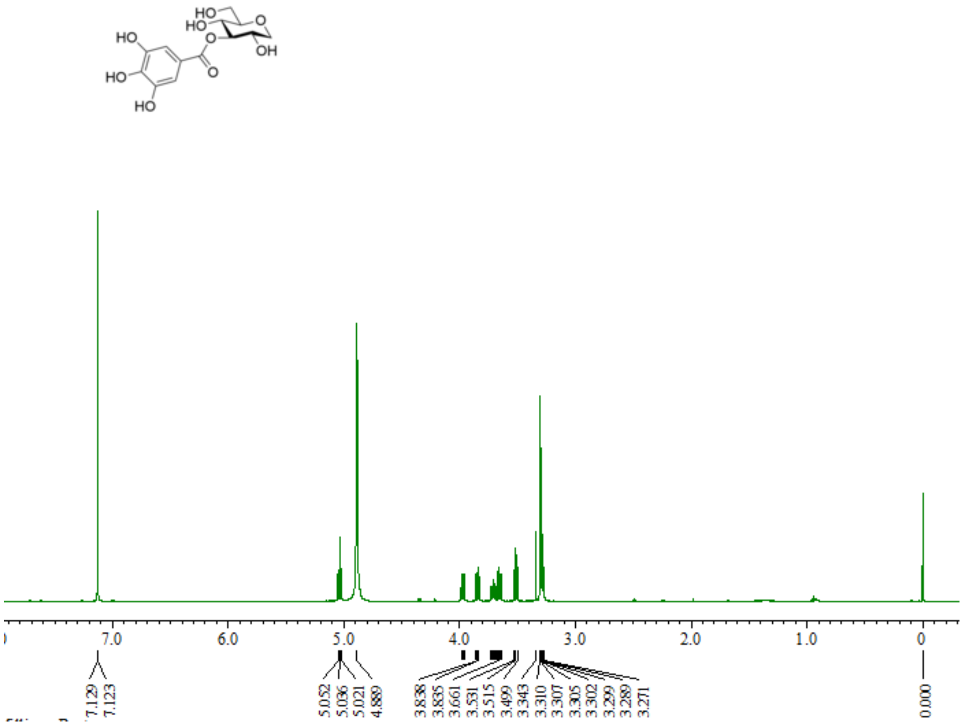

^{13}C NMR of compound **32** in CD_3OD

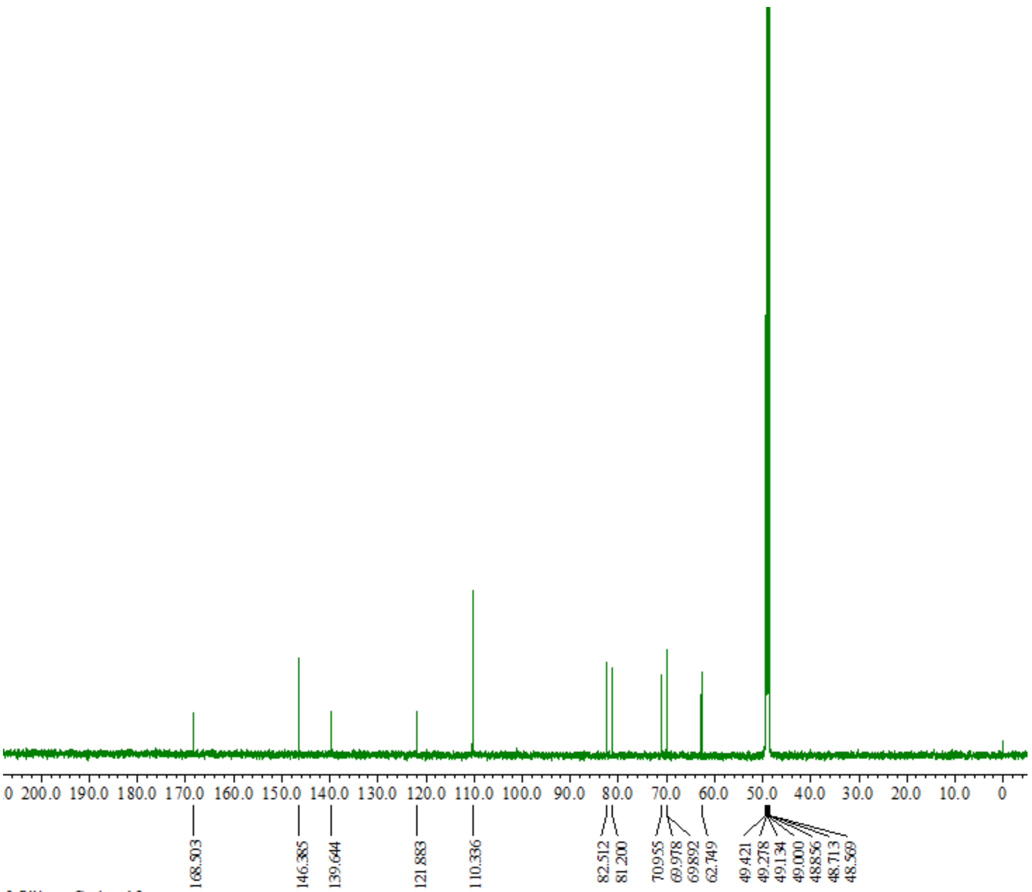

^{1}H NMR of compound **33** in Acetone-d_6

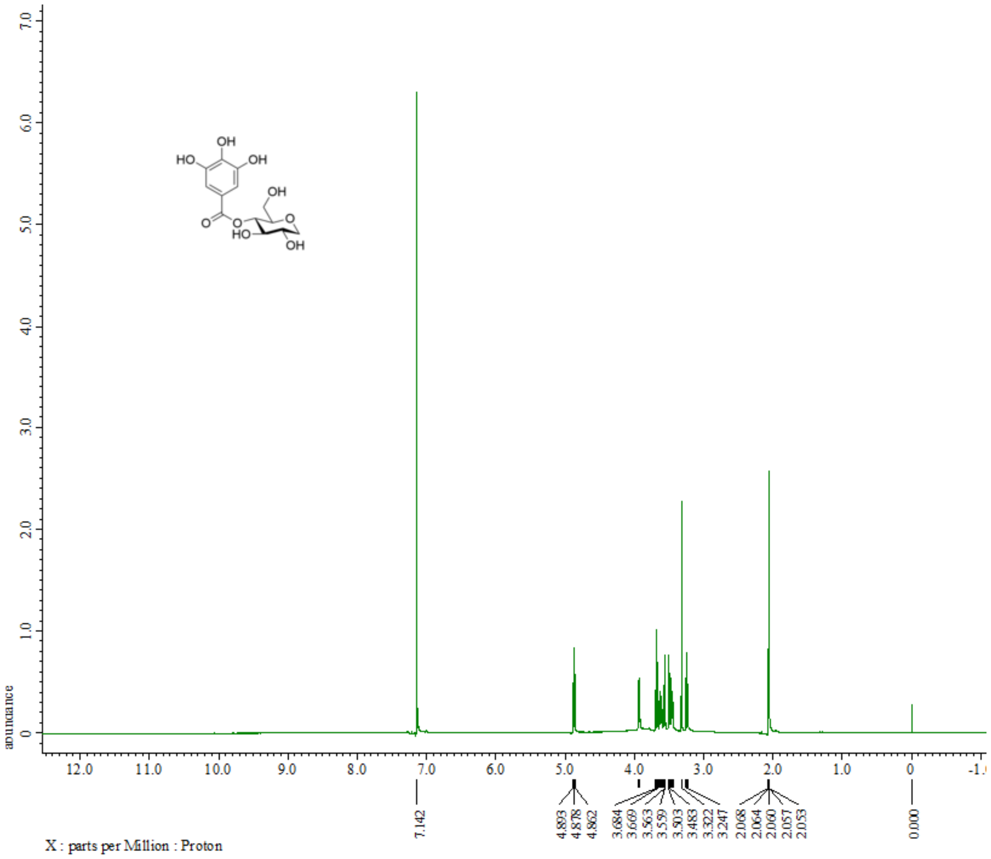

^{13}C NMR of compound **33** in Acetone-d_6

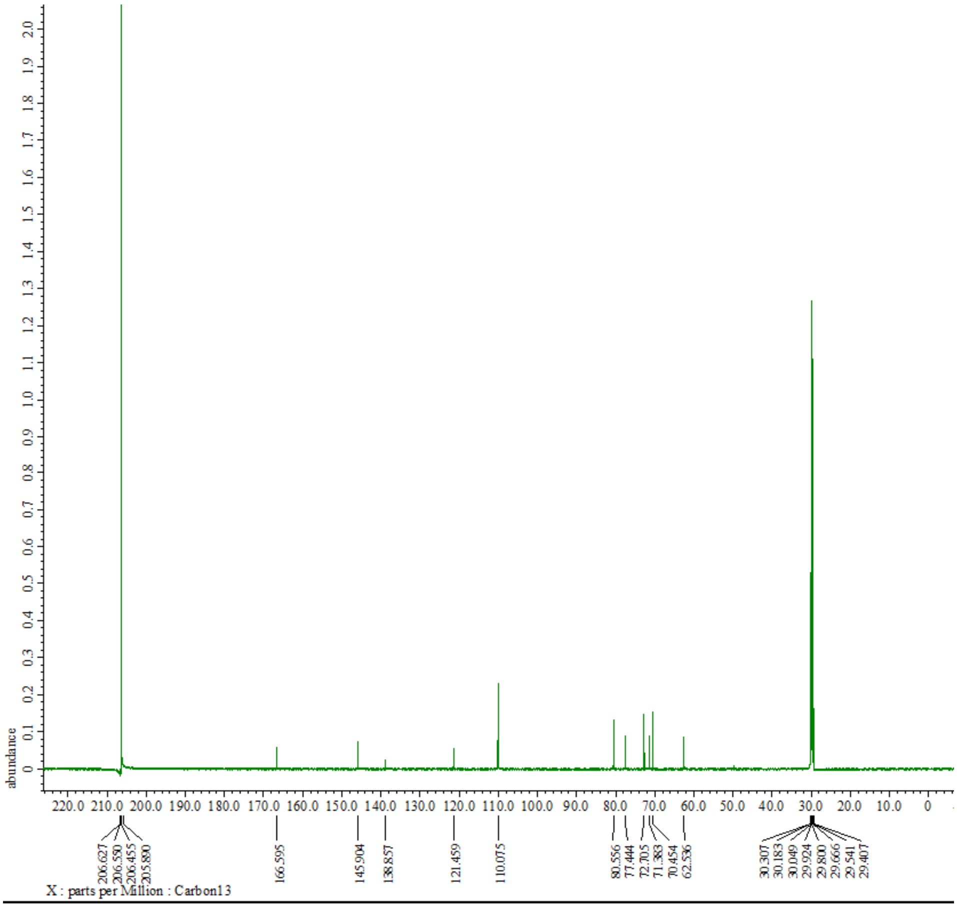

^{1}H NMR of compound **34** in CD_3OD

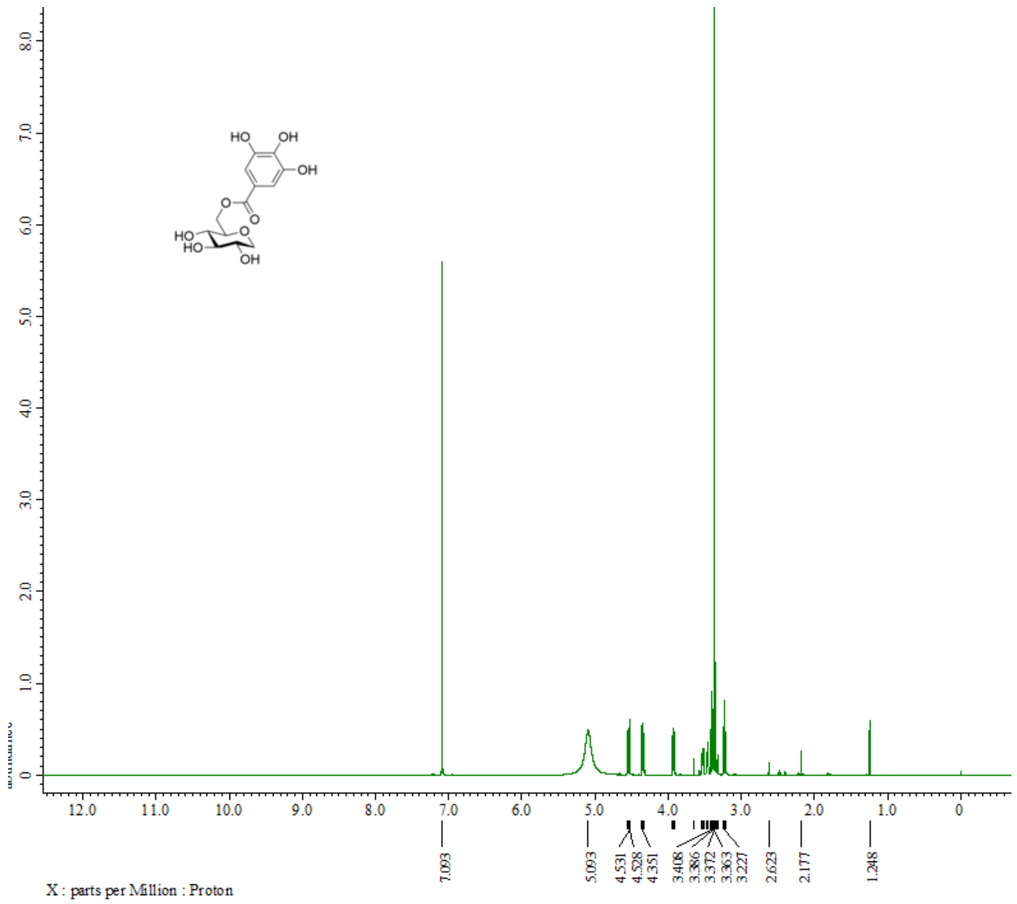

^{13}C NMR of compound **34** in CD_3OD

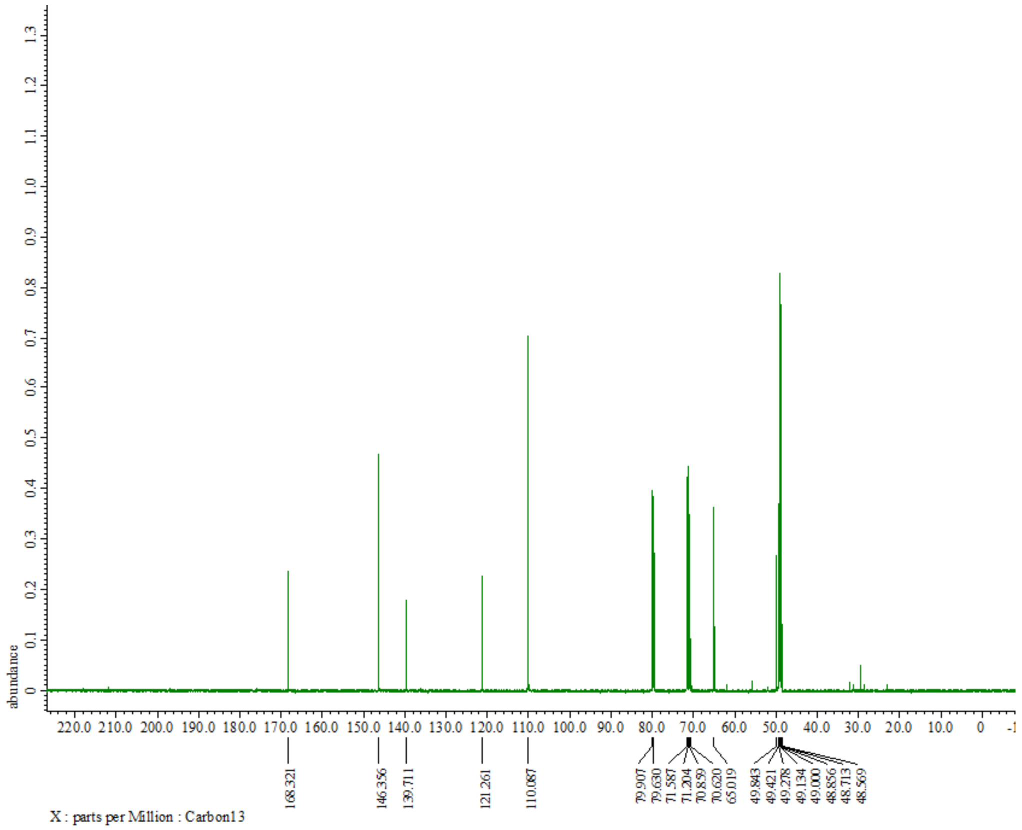

^{1}H NMR of compound **35** in CD_3OD

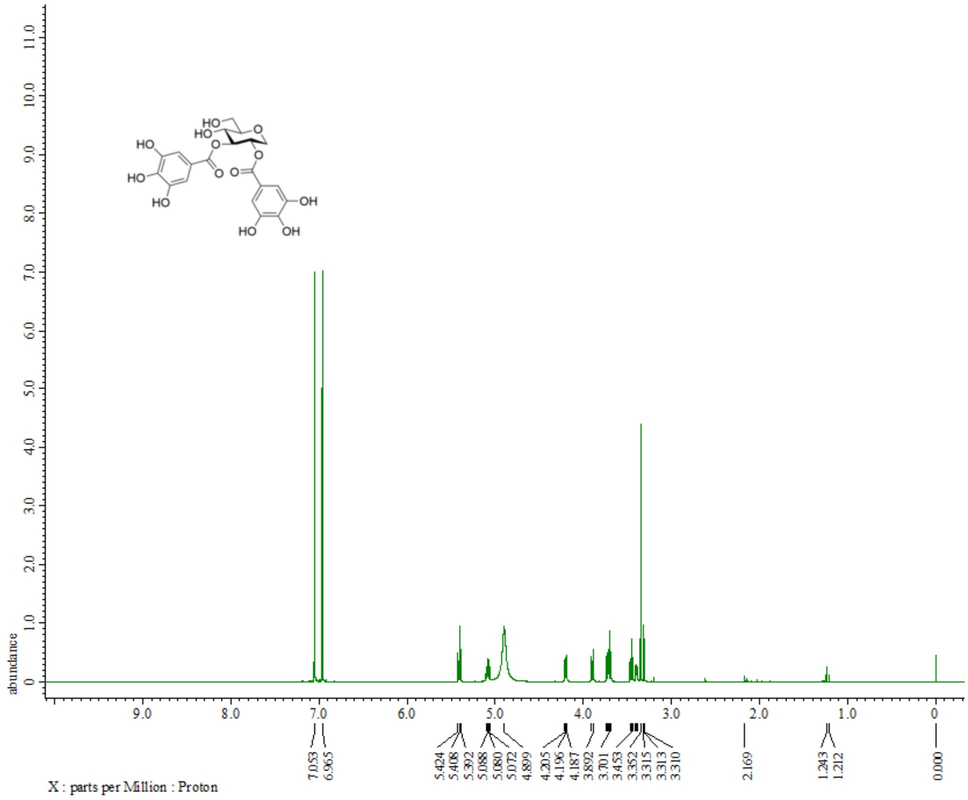

^{13}C NMR of compound **35** in CD_3OD

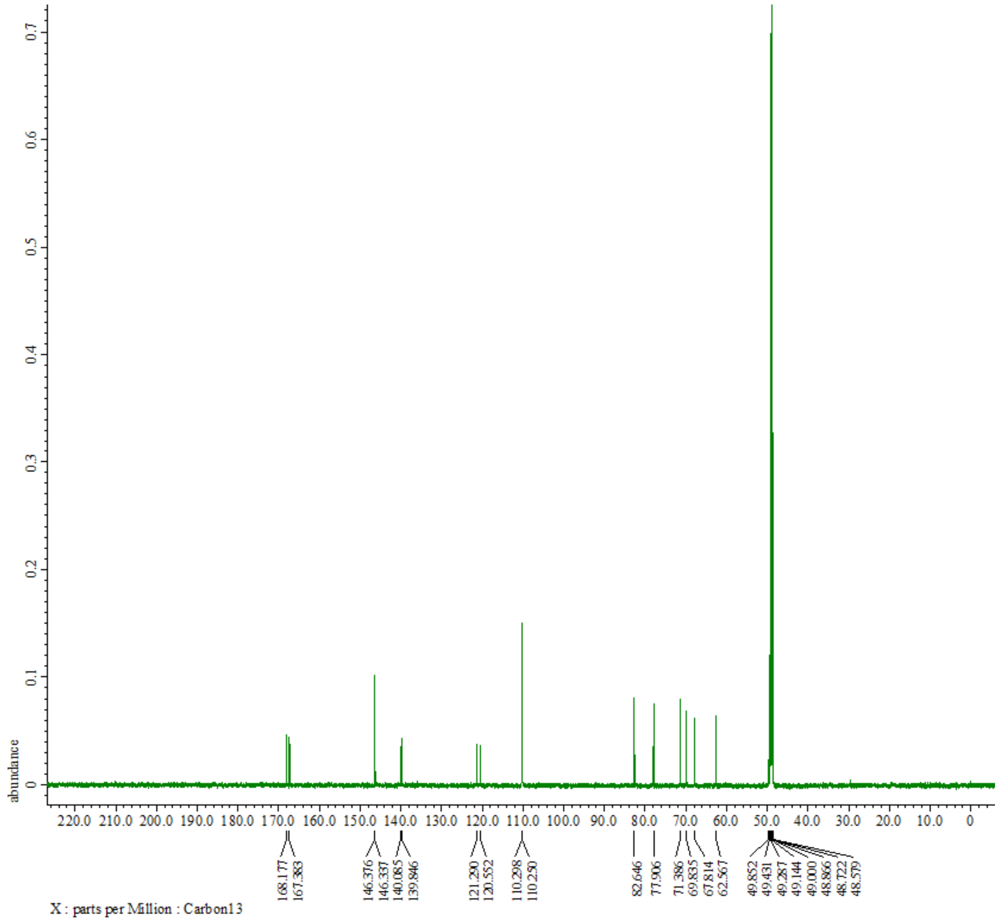

^{1}H NMR of compound **36** in Acetone-d_6

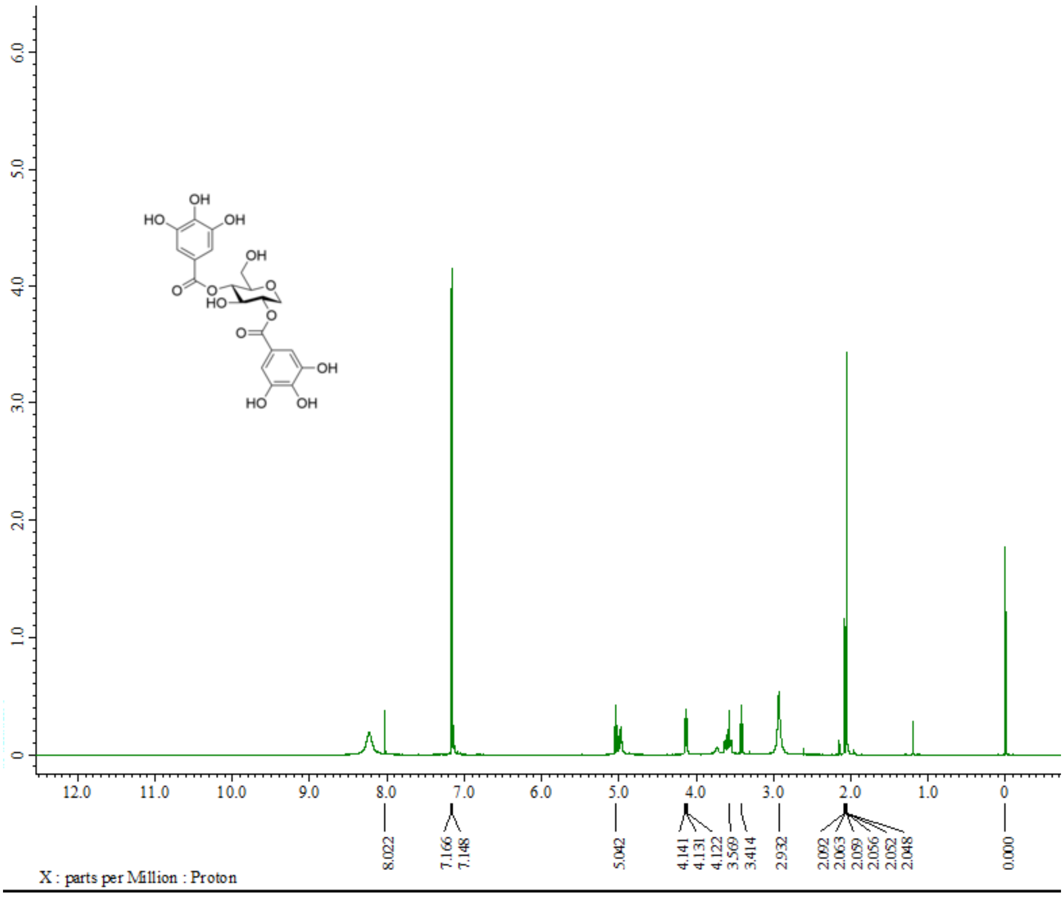

^{13}C NMR of compound **36** in Acetone-d_6

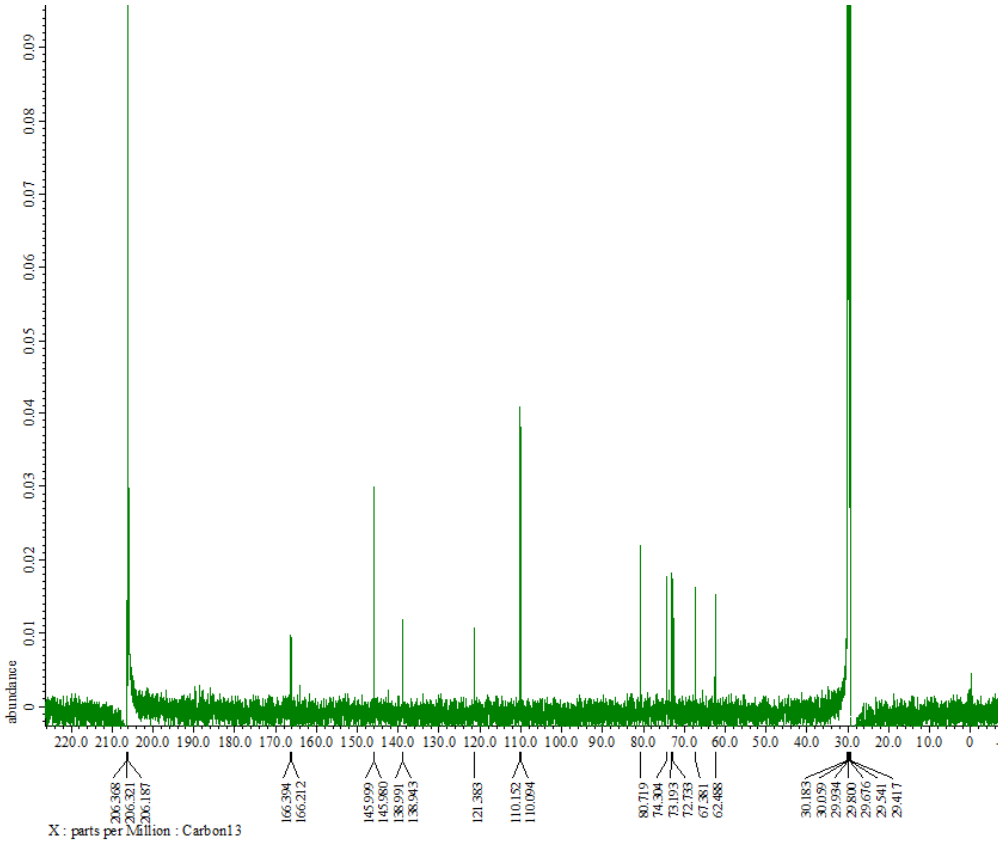

^{1}H NMR of compound **37** in Acetone-d_6

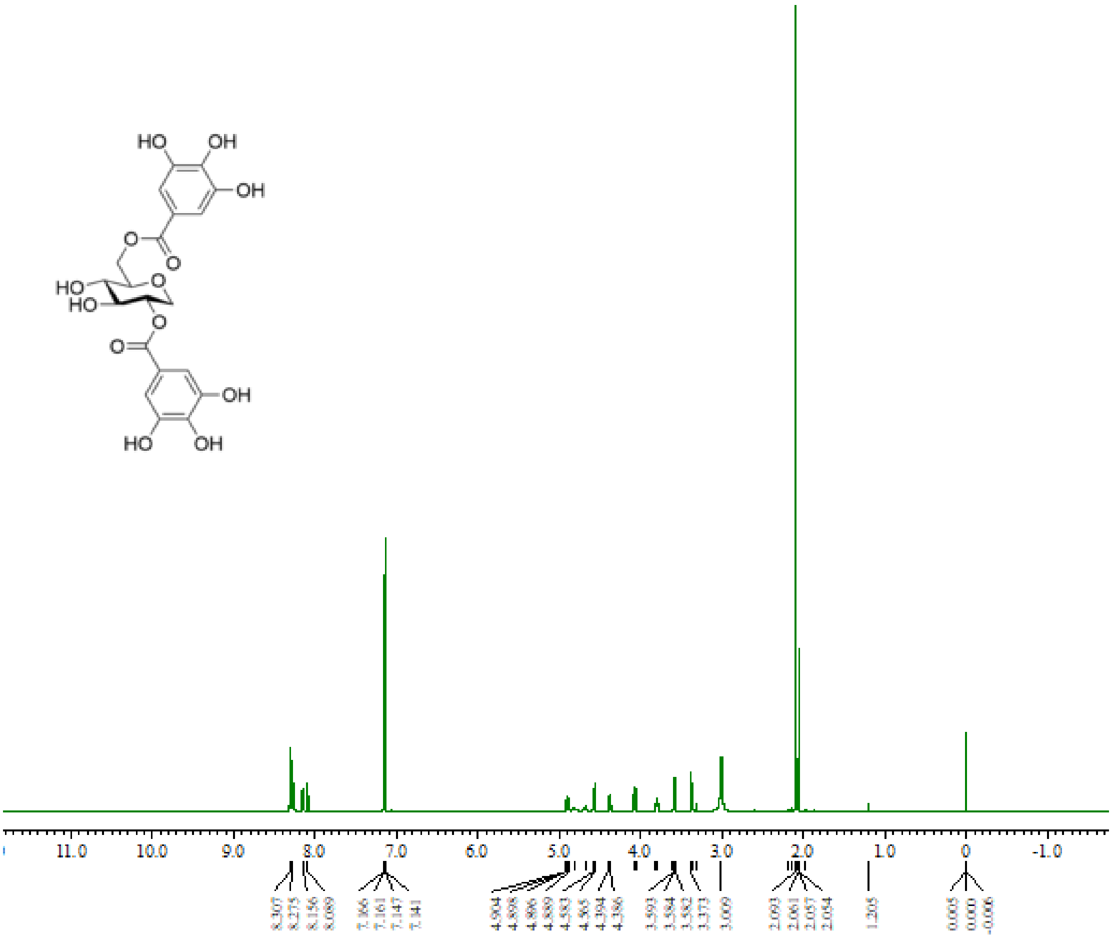

^{13}C NMR of compound **37** in Acetone-d_6

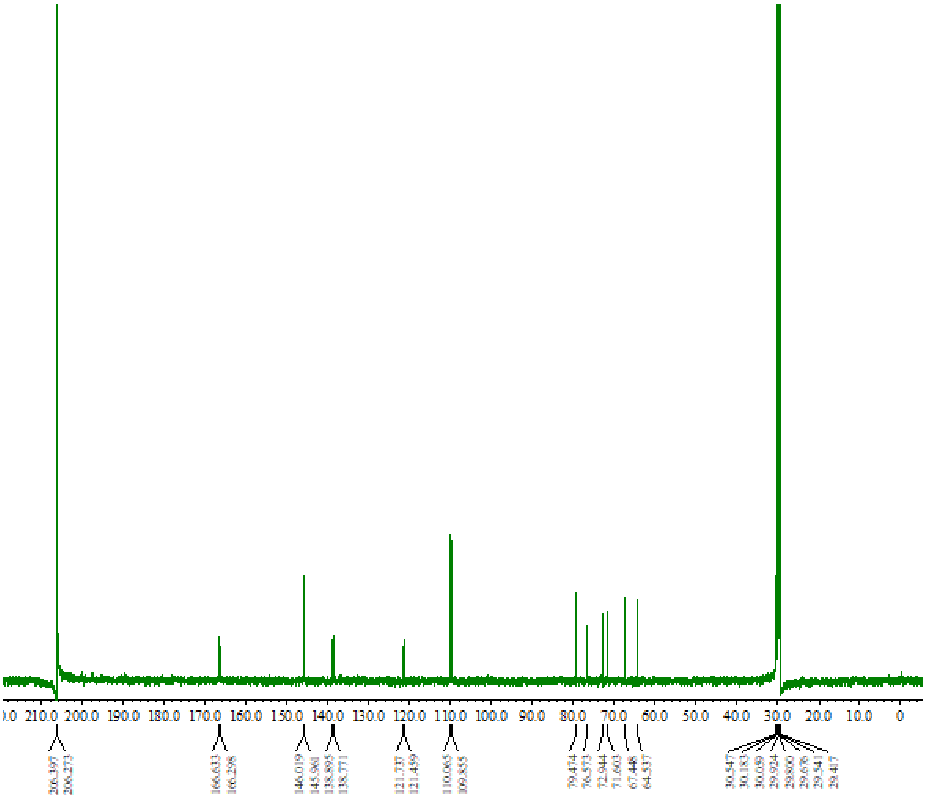

^{1}H NMR of compound **38** in Acetone-d_6

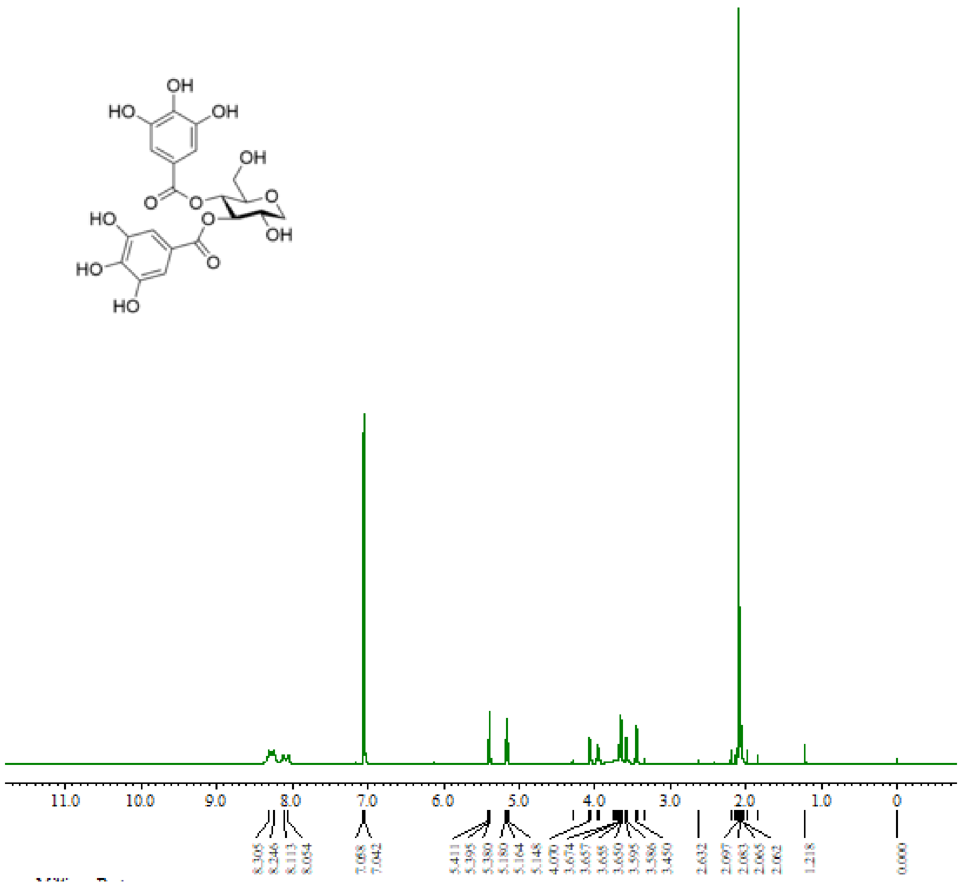

^{13}C NMR of compound **38** in Acetone-d_6

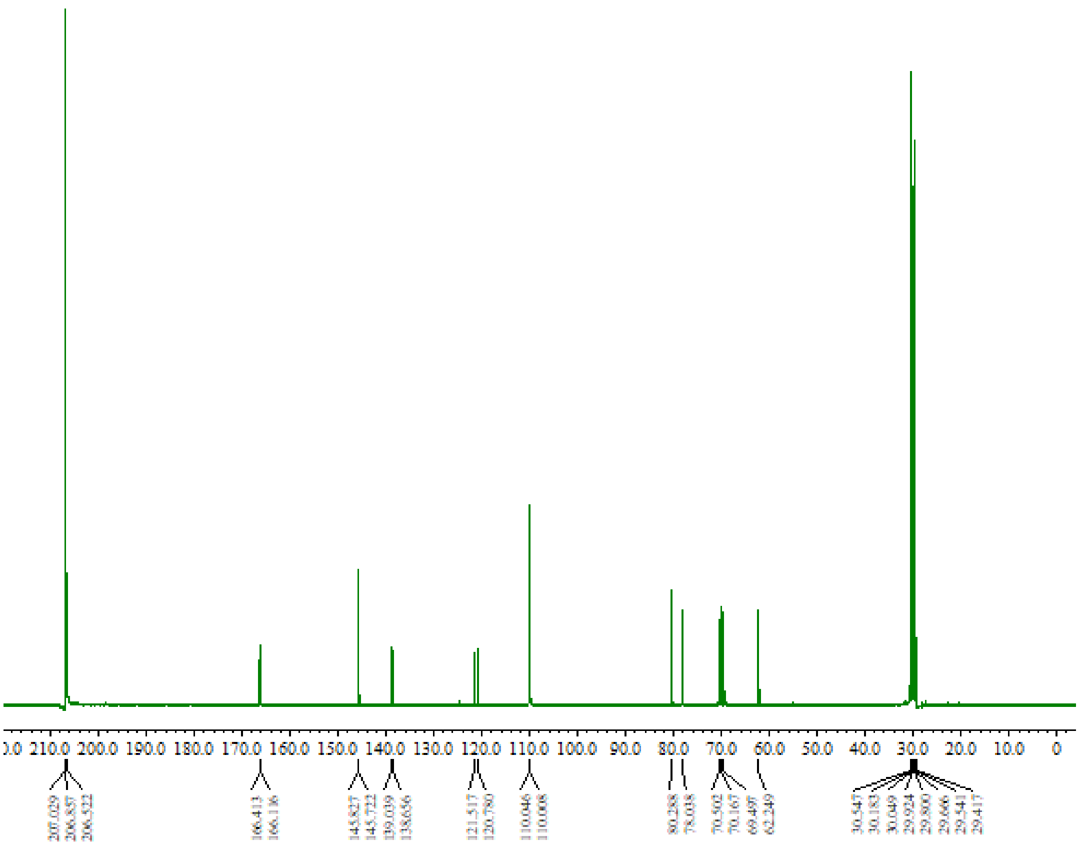

^{1}H NMR of compound **39** in Acetone-d_6

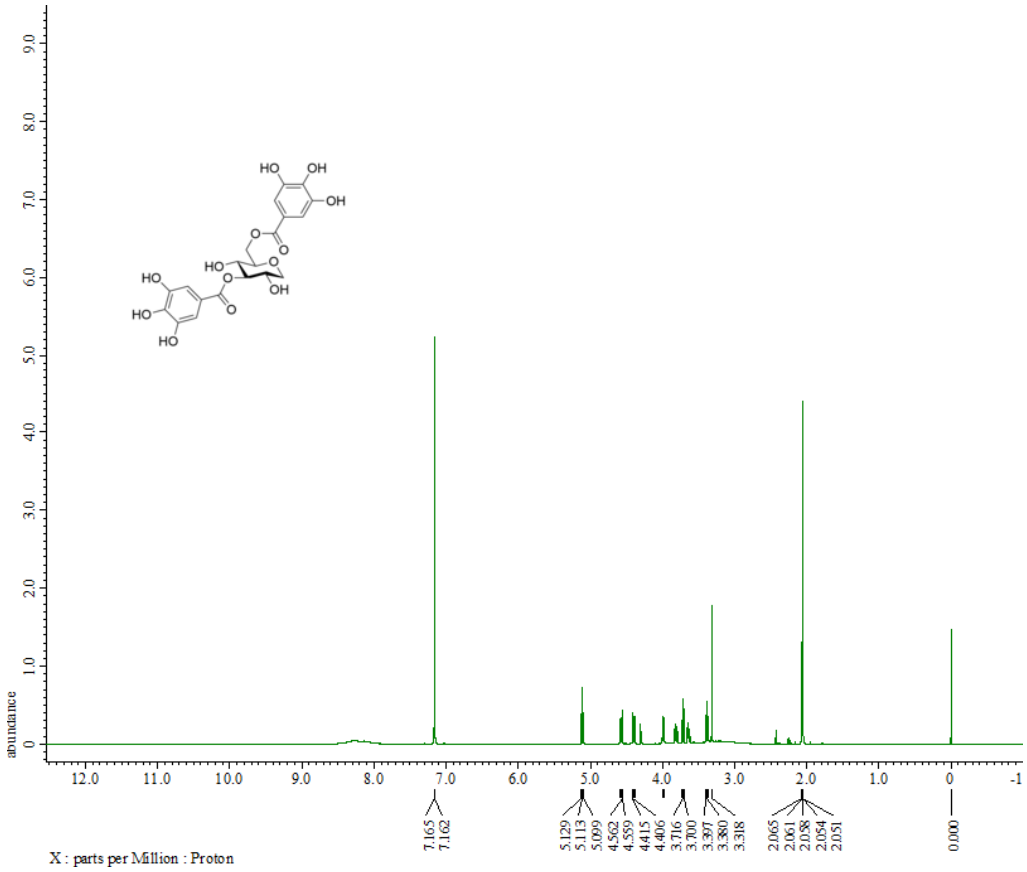

^{13}C NMR of compound **39** in Acetone-d_6

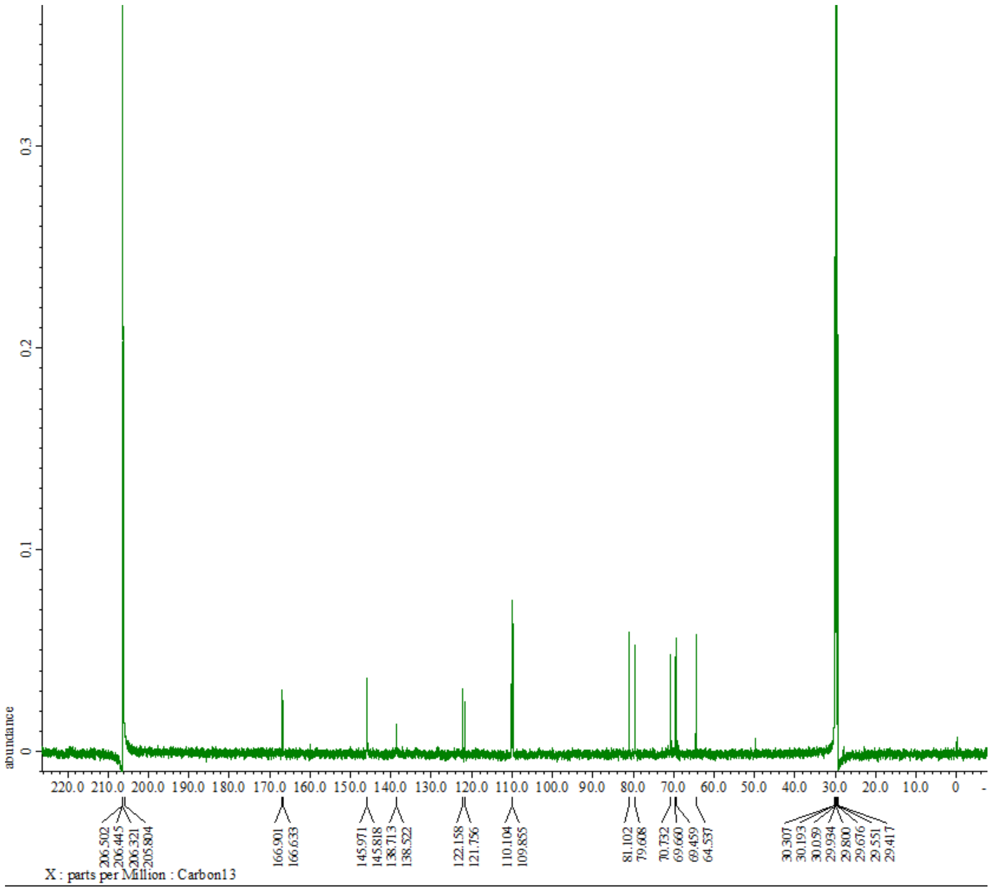

^{1}H NMR of compound **40** in Acetone-d_6

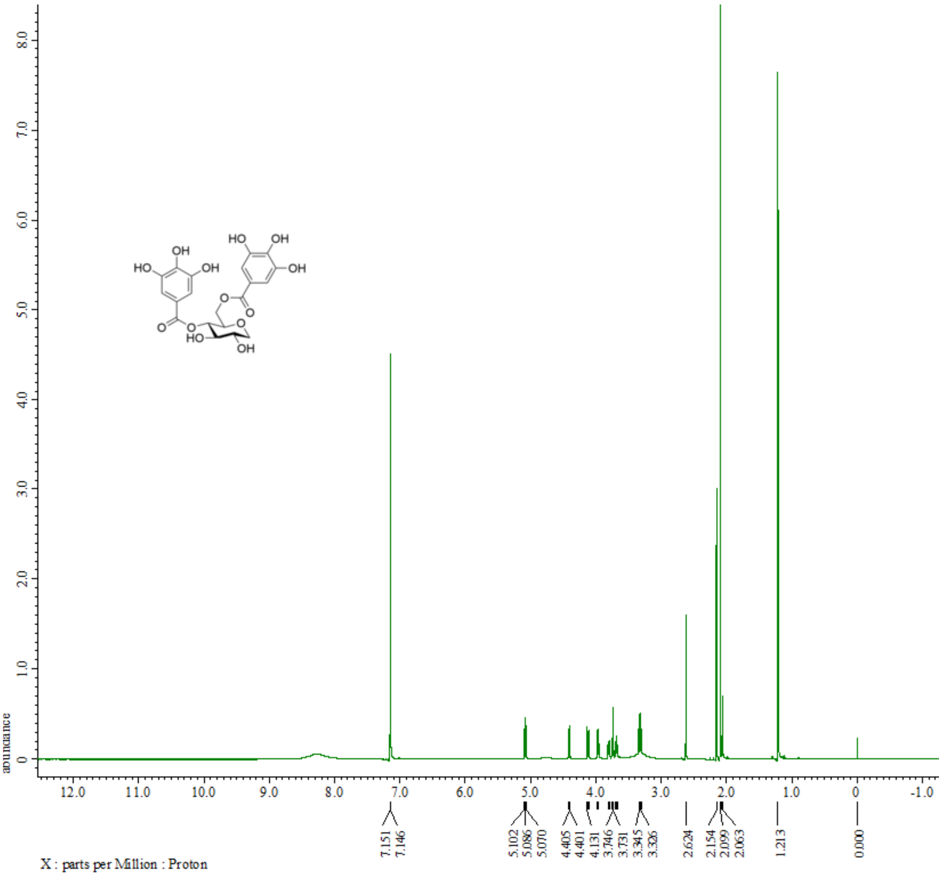

^{13}C NMR of compound **40** in Acetone-d_6

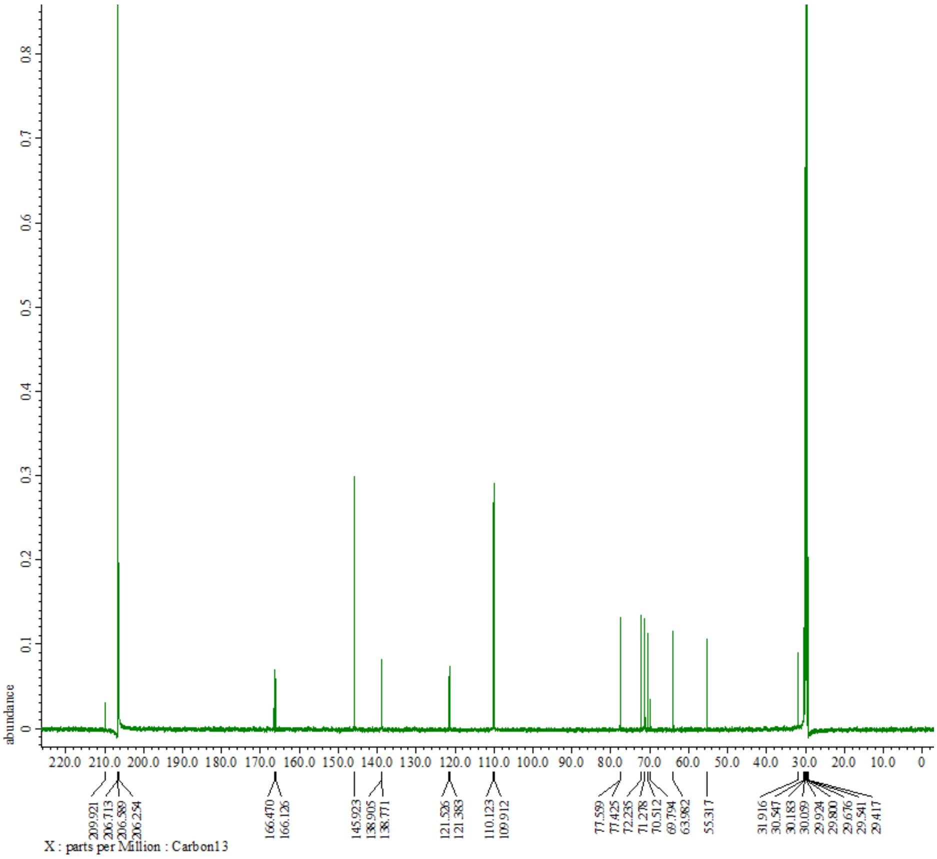

^{1}H NMR of compound **41** in Acetone-d_6

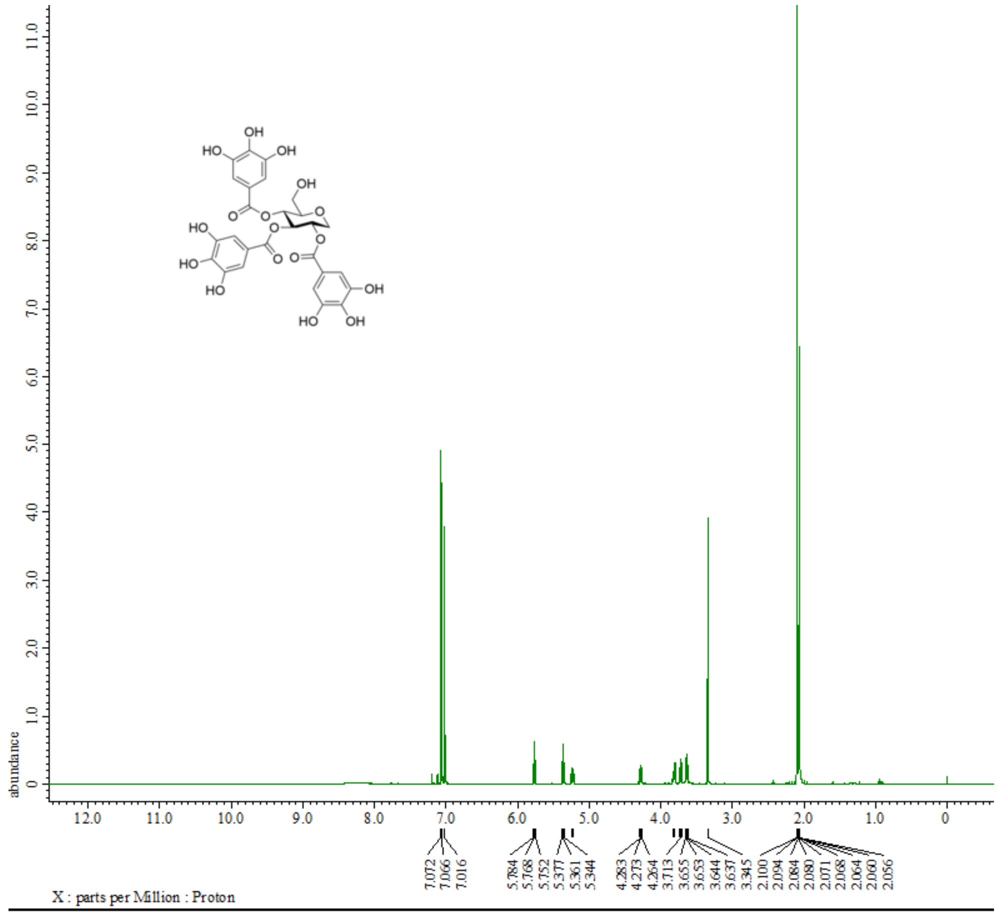

^{13}C NMR of compound **41** in Acetone-d_6

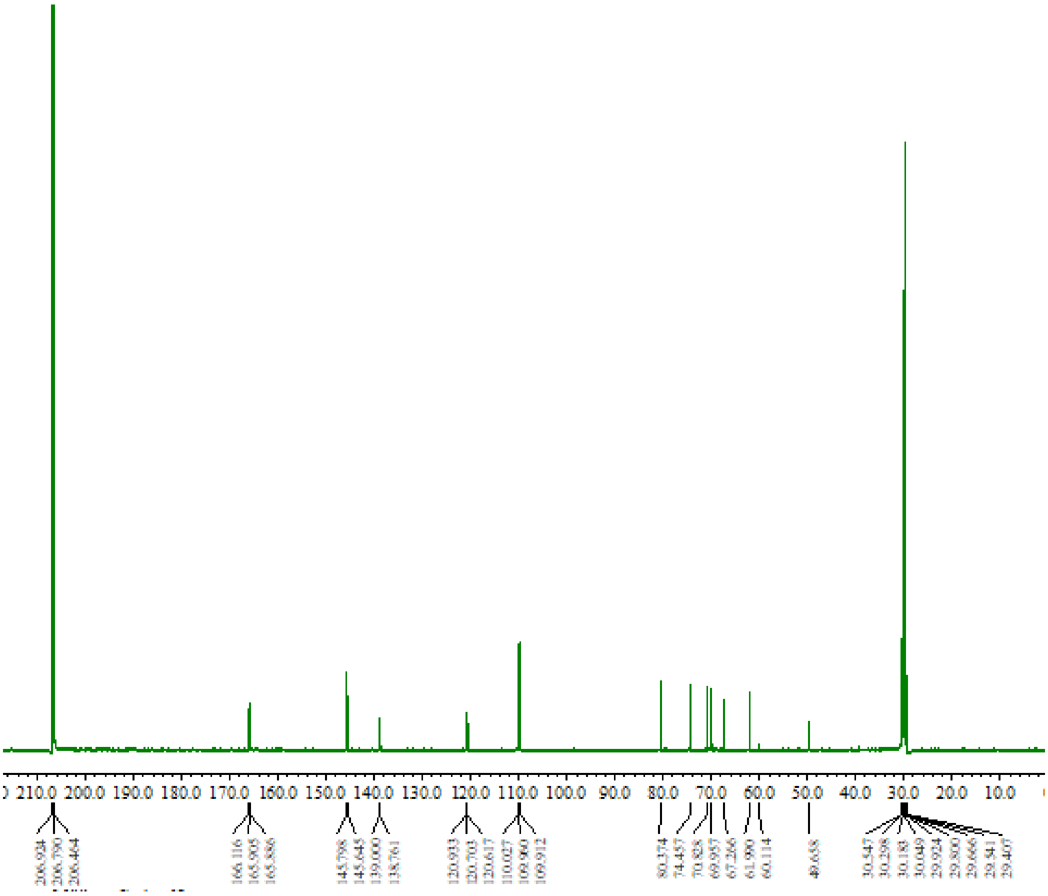

^{1}H NMR of compound **42** in Acetone-d_6

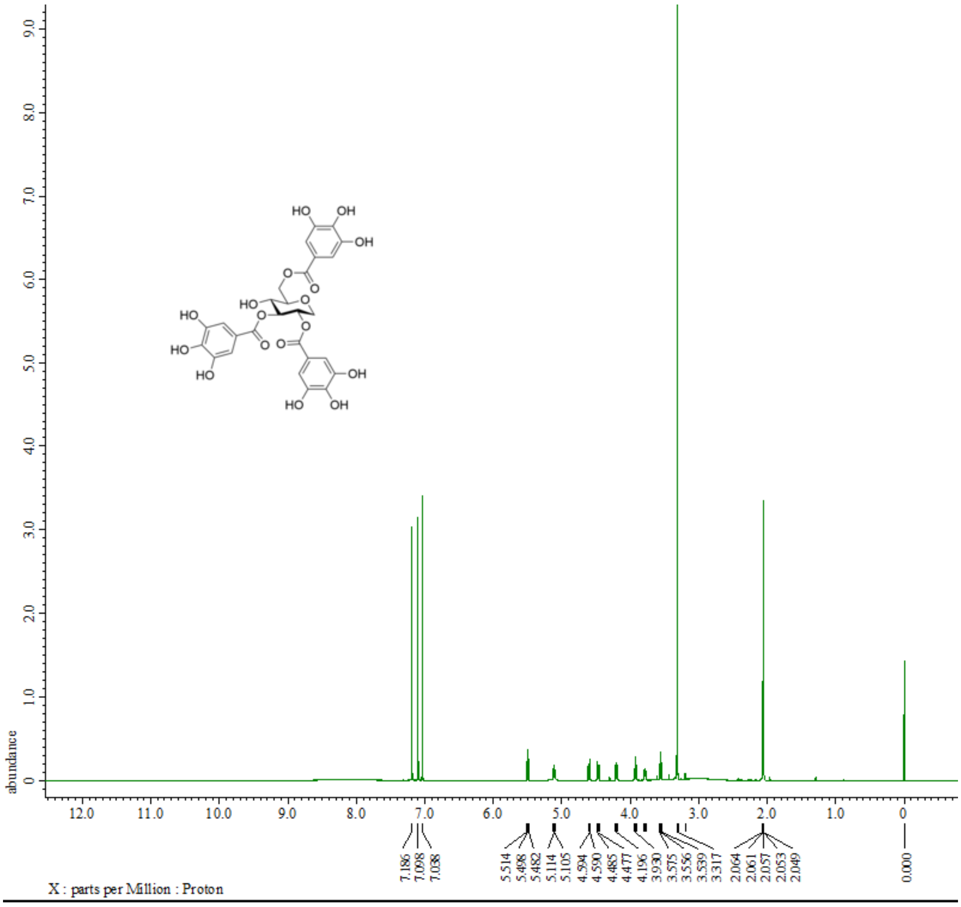

^{13}C NMR of compound **42** in Acetone-d_6

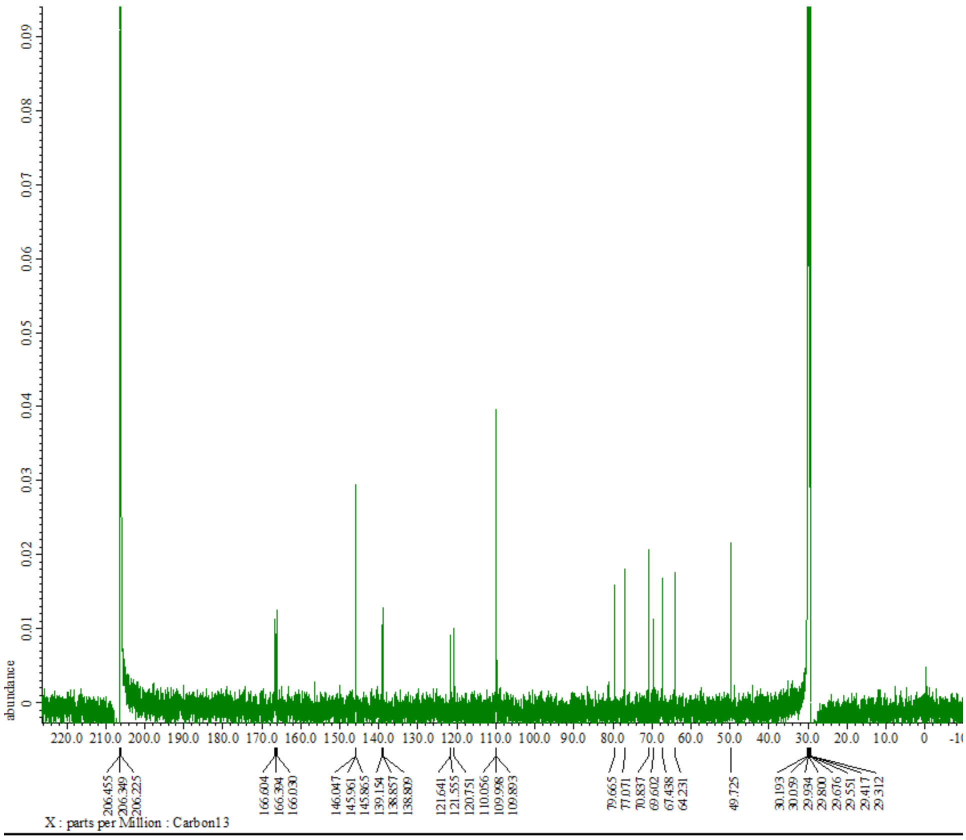

^{1}H NMR of compound **43** in Acetone-d_6

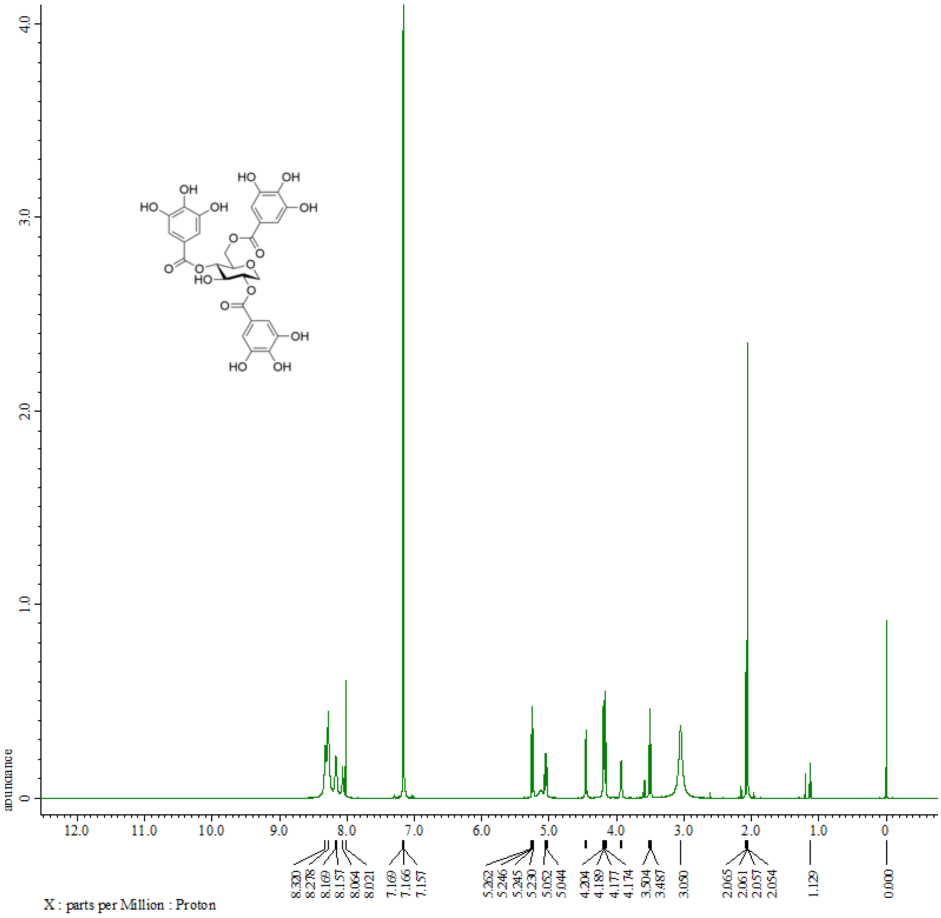

^{13}C NMR of compound **43** in Acetone-d_6

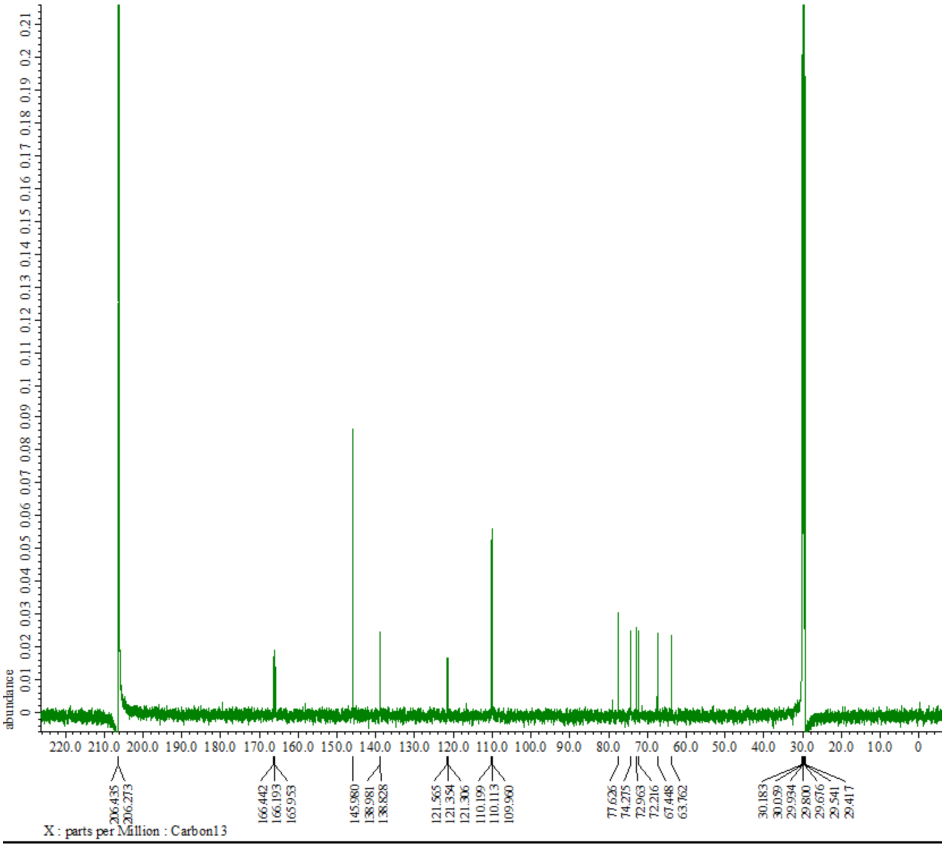

^{1}H NMR of compound **44** in Acetone-d_6

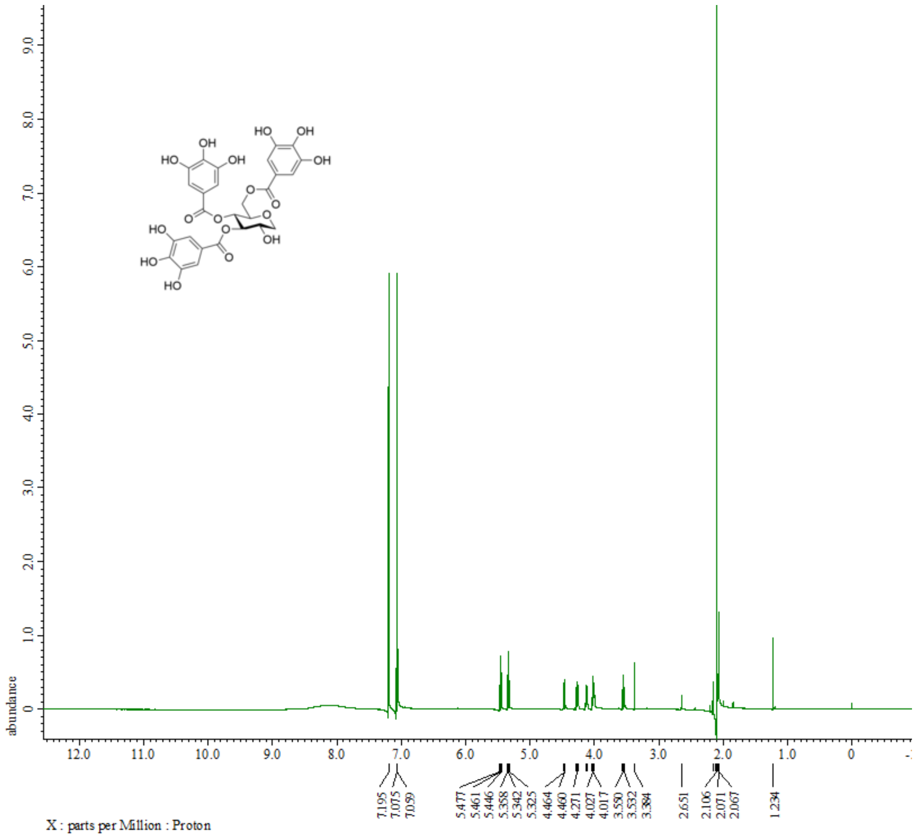

^{13}C NMR of compound **44** in Acetone-d_6

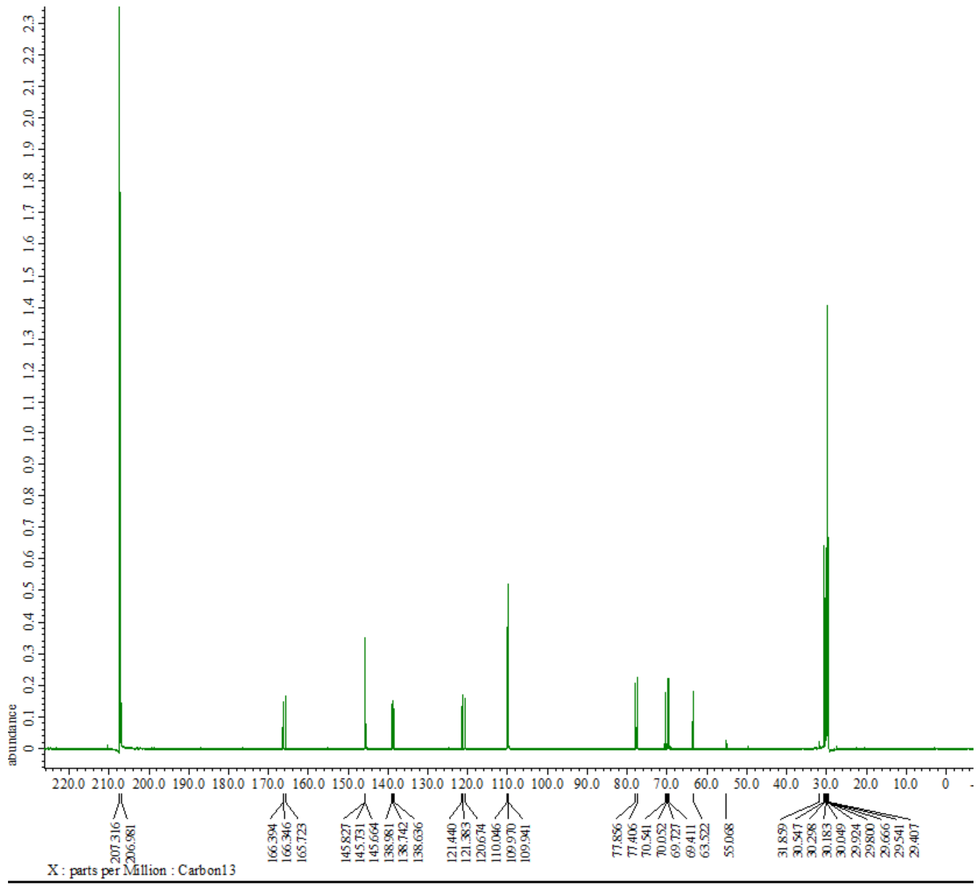

^{1}H NMR of compound **54** in Acetone-d_6

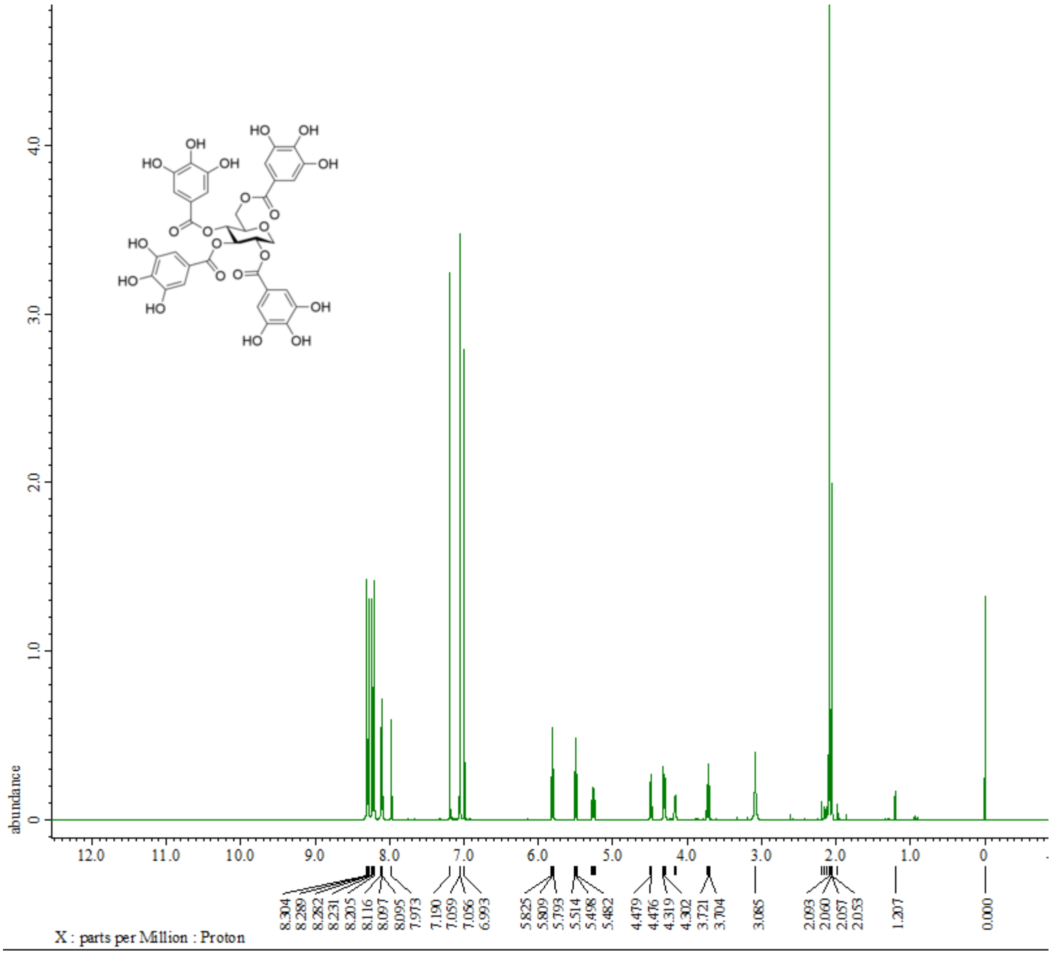

^{13}C NMR of compound **54** in Acetone-d_6

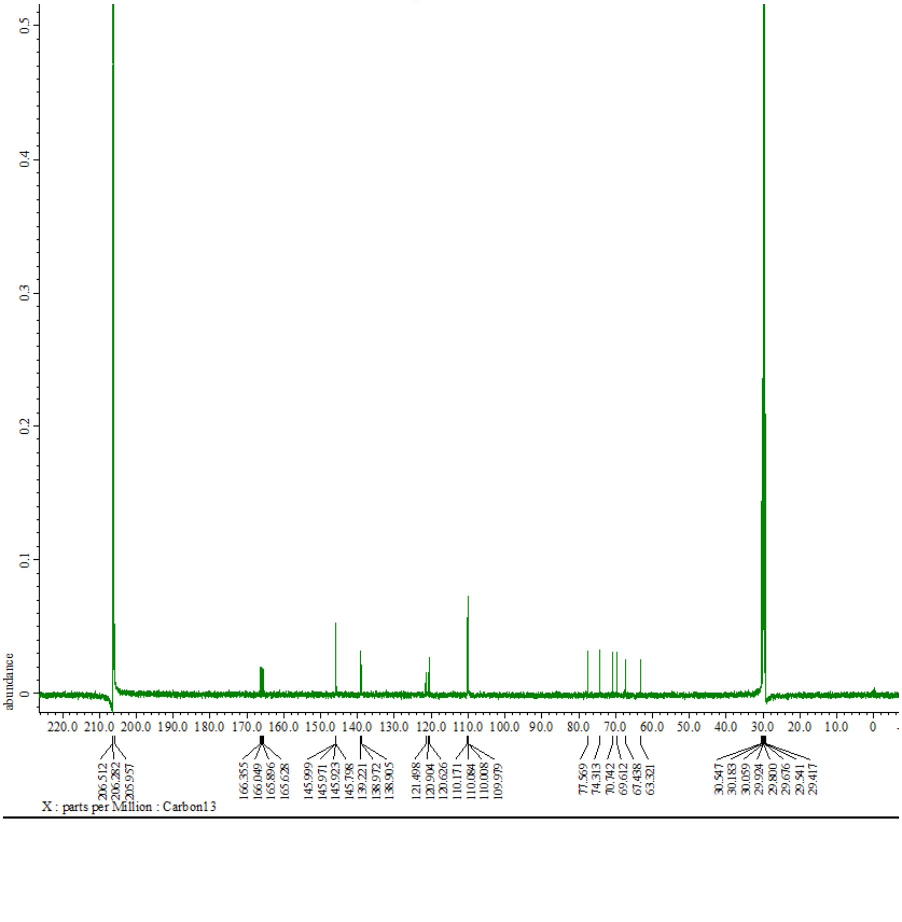

^{1}H NMR of compound **55** in Acetone-d_6

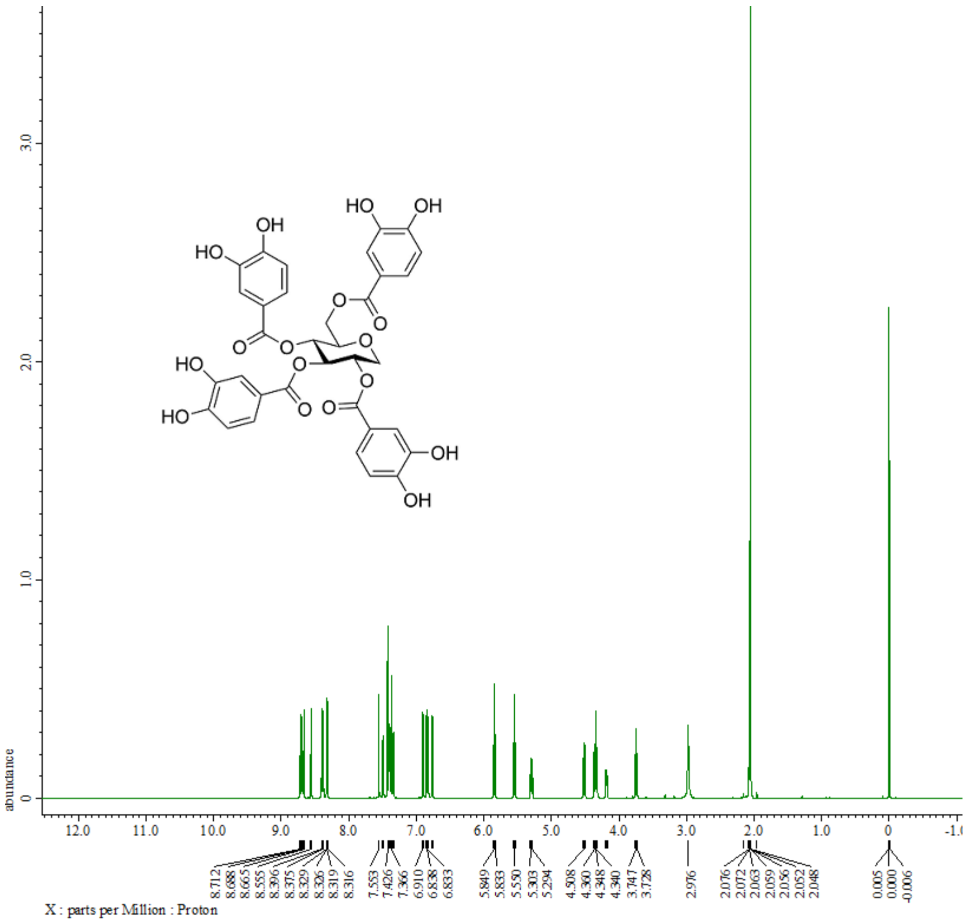

^{13}C NMR of compound **55** in Acetone-d_6

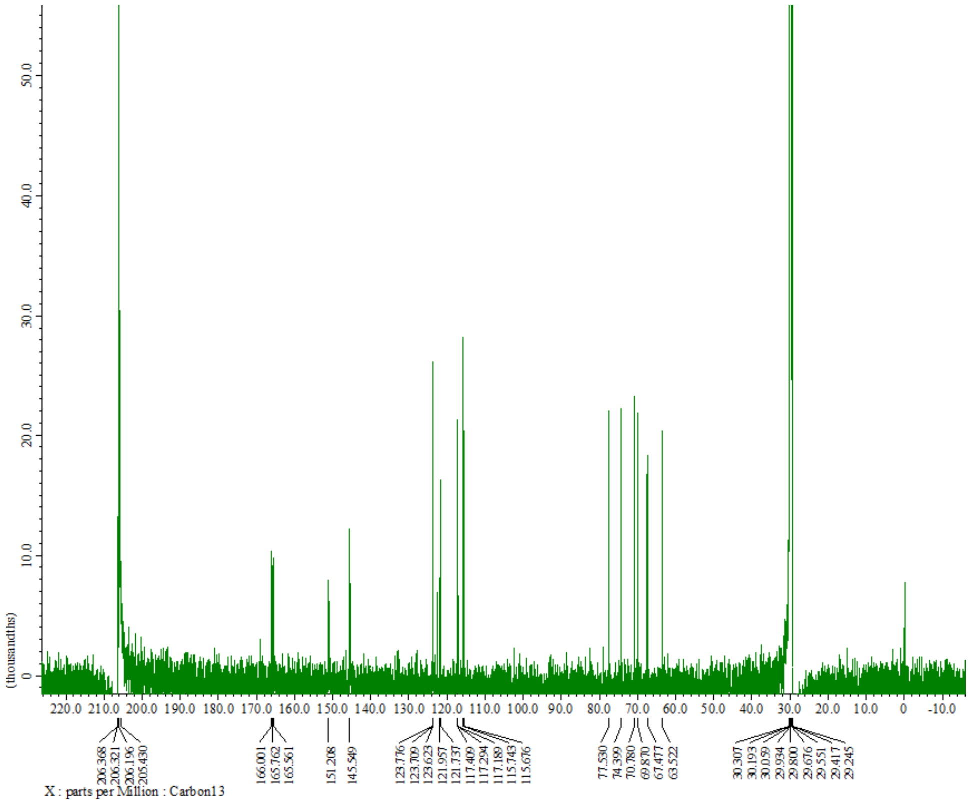

^{1}H NMR of compound **56** in Acetone-d_6

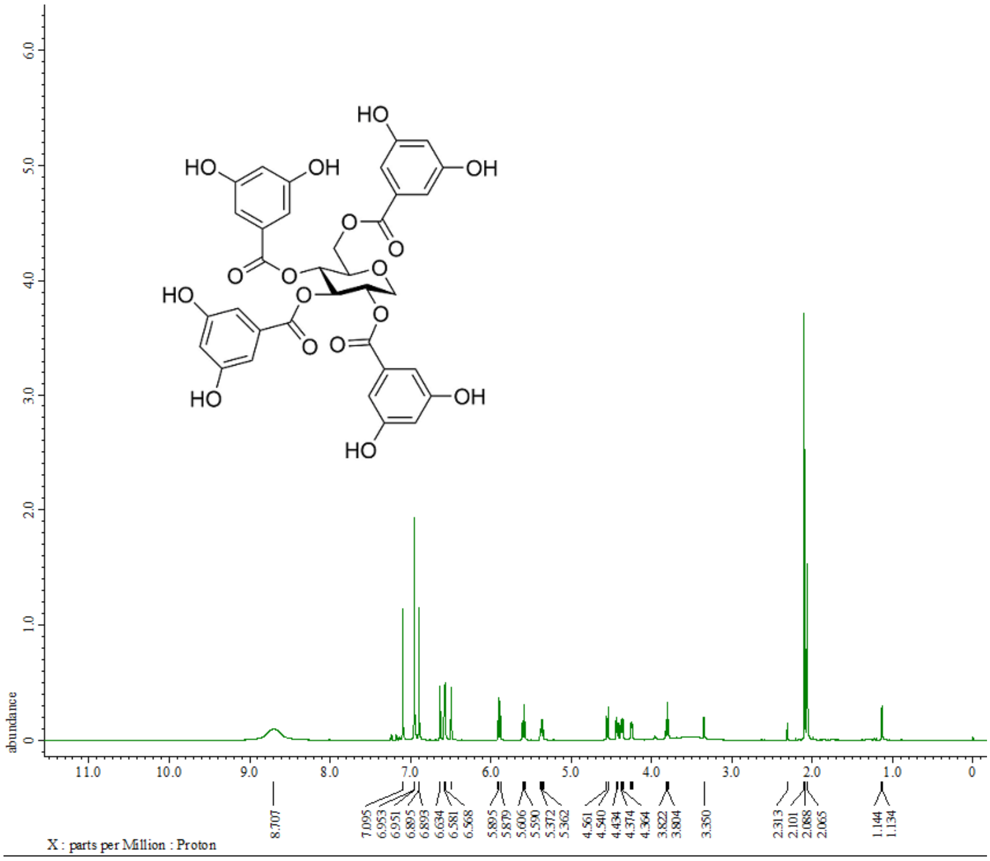

^{13}C NMR of compound **56** in Acetone-d_6

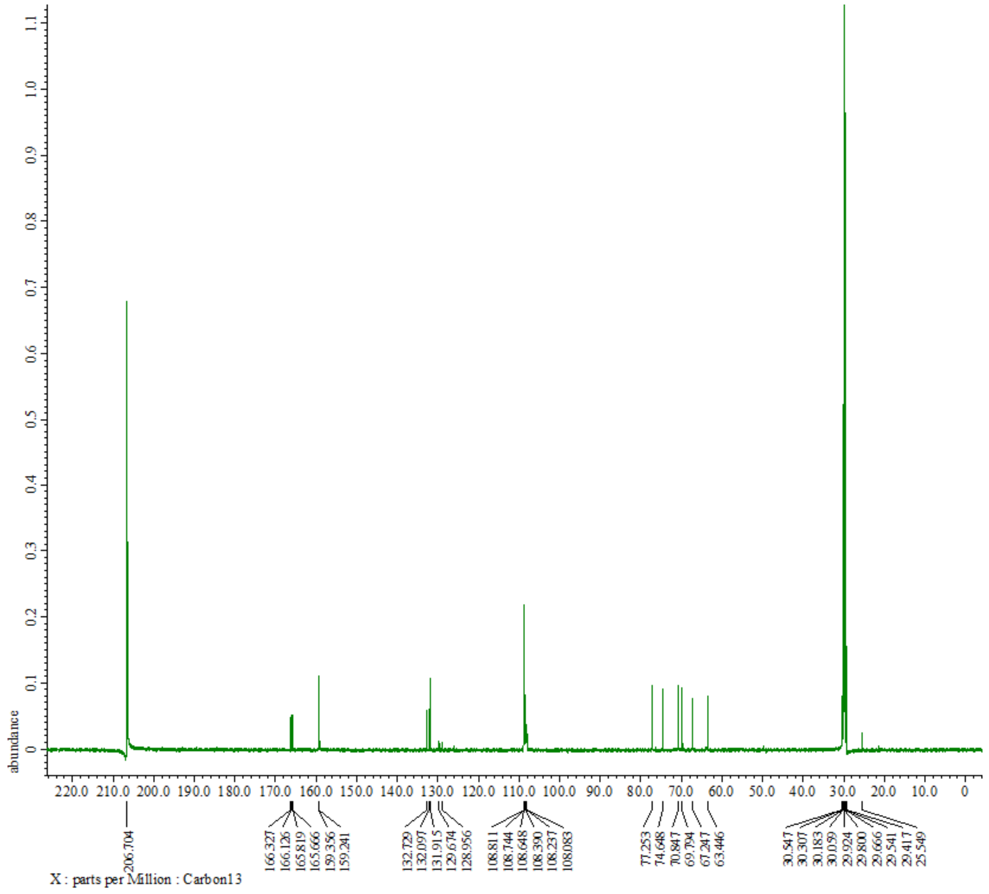

^{1}H NMR of compound **57** in Acetone-d_6

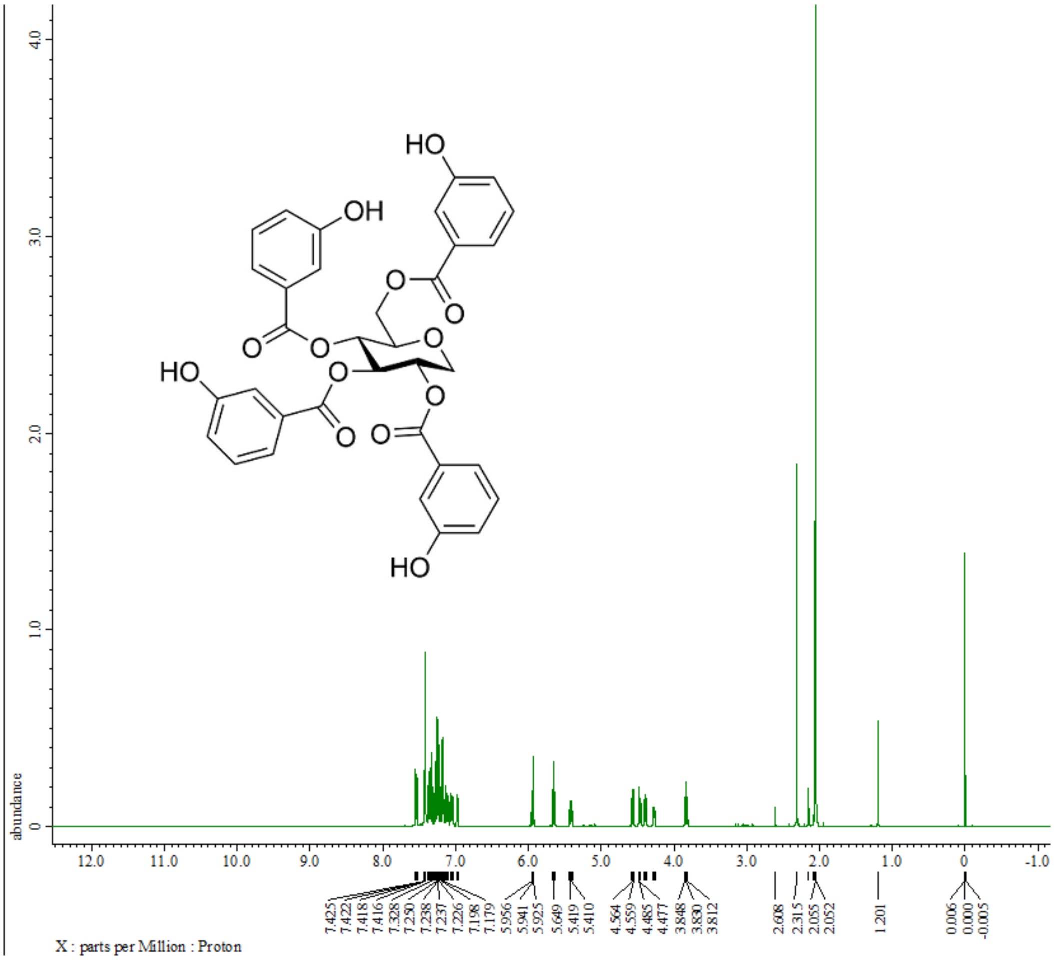

^{13}C NMR of compound **57** in Acetone-d_6

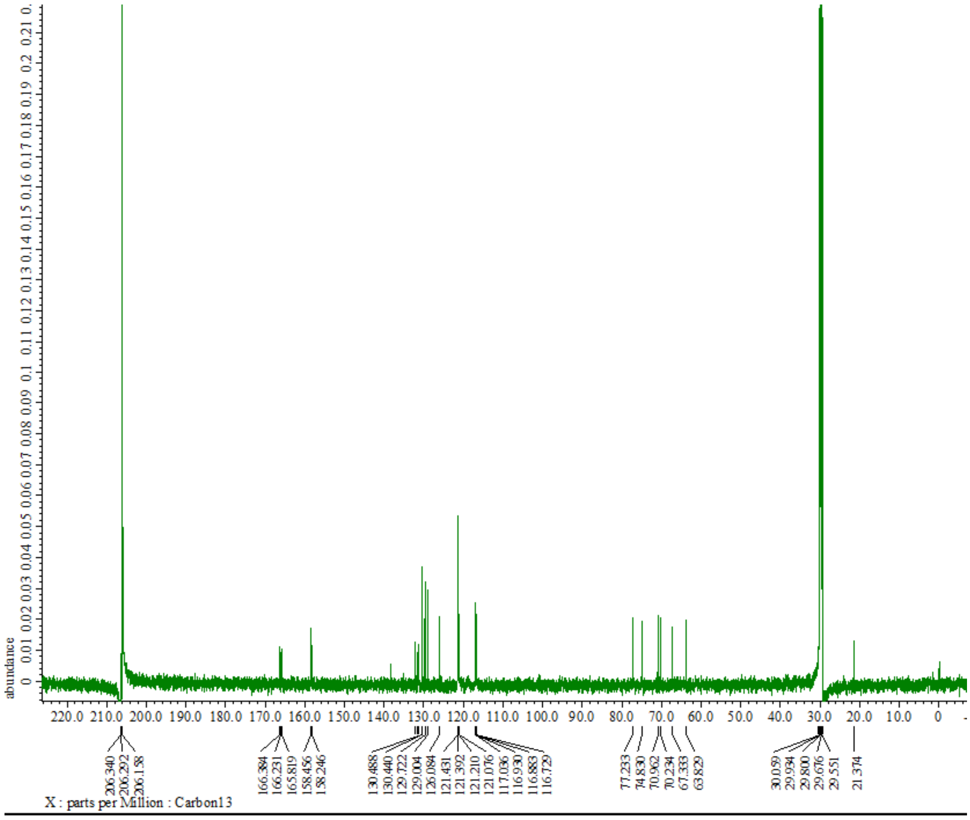

^1H NMR of compound **58** in Acetone-d_6

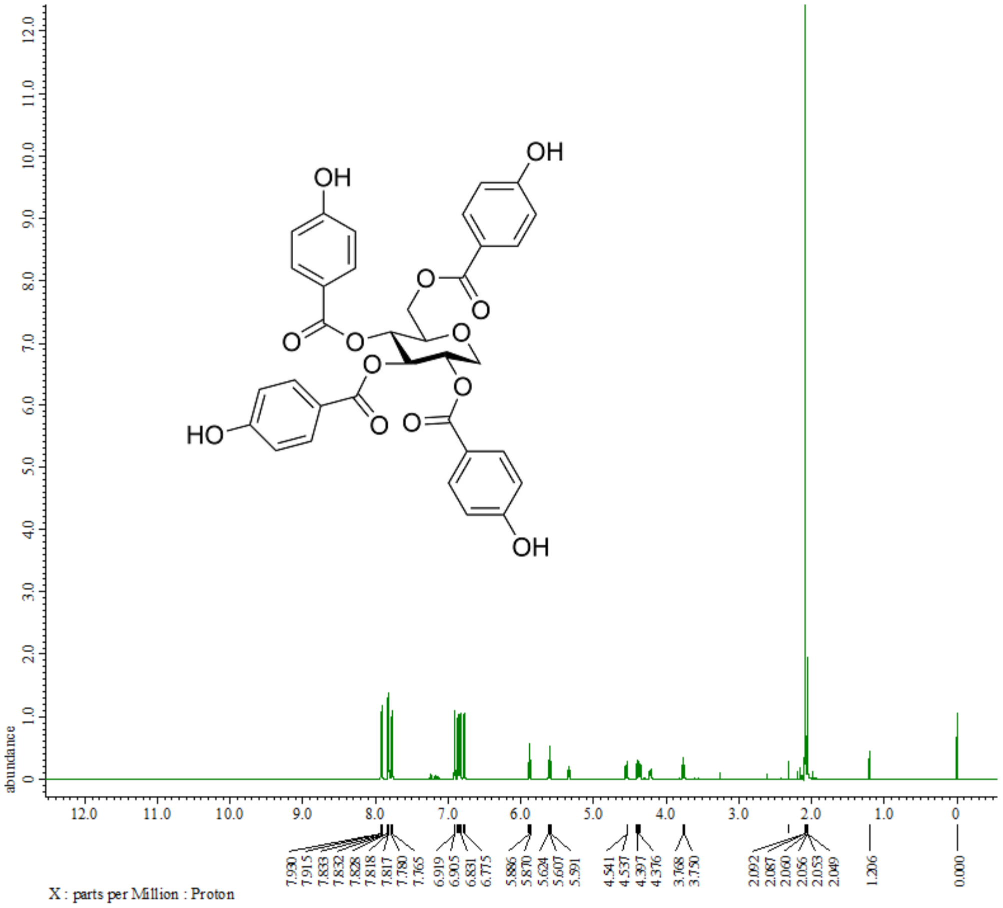

^{13}C NMR of compound **58** in Acetone-d_6

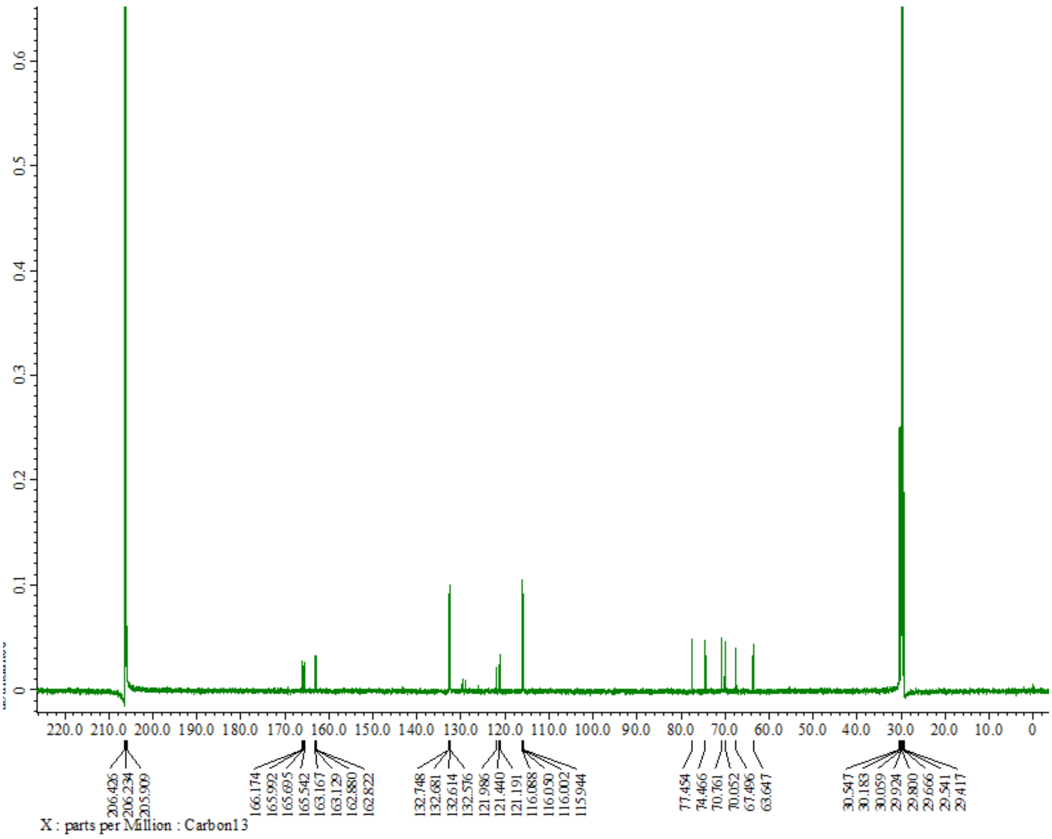

^{1}H NMR of compound **61** in Acetone-d_6

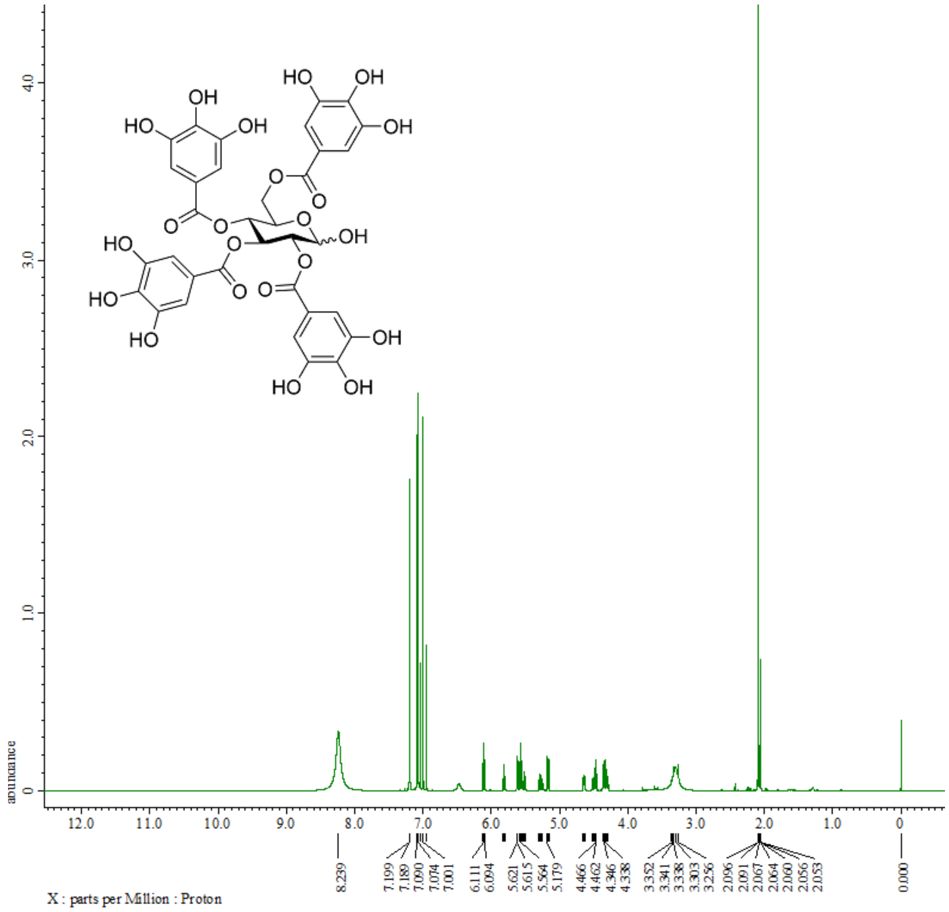

^{13}C NMR of compound **61** in Acetone-d_6

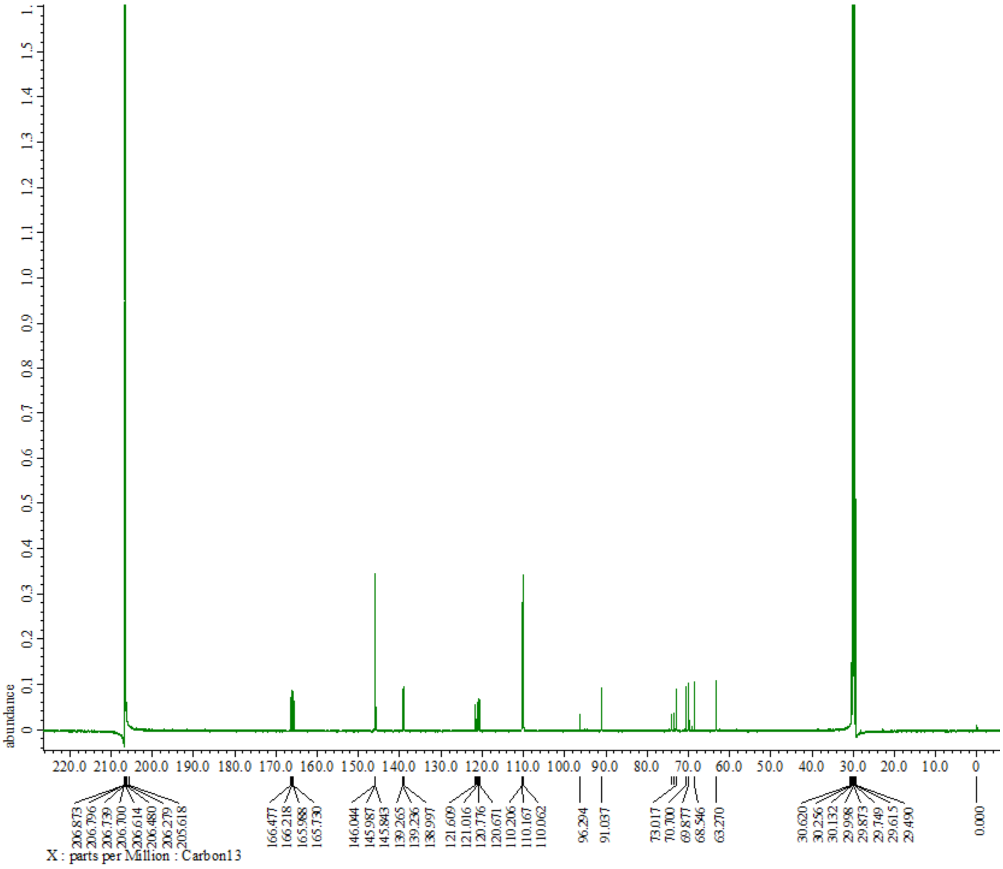

^{1}H NMR of compound **67** in Acetone-d_6

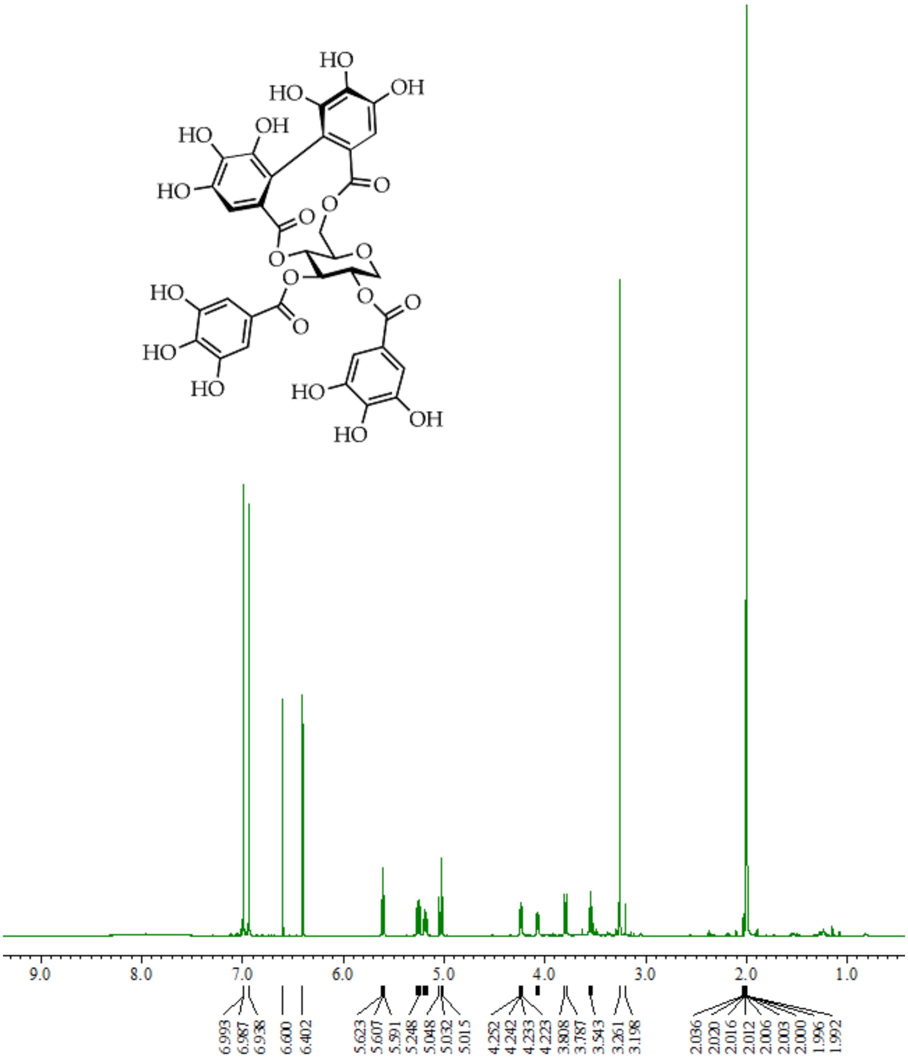

^{13}C NMR of compound **67** in CD_3OD

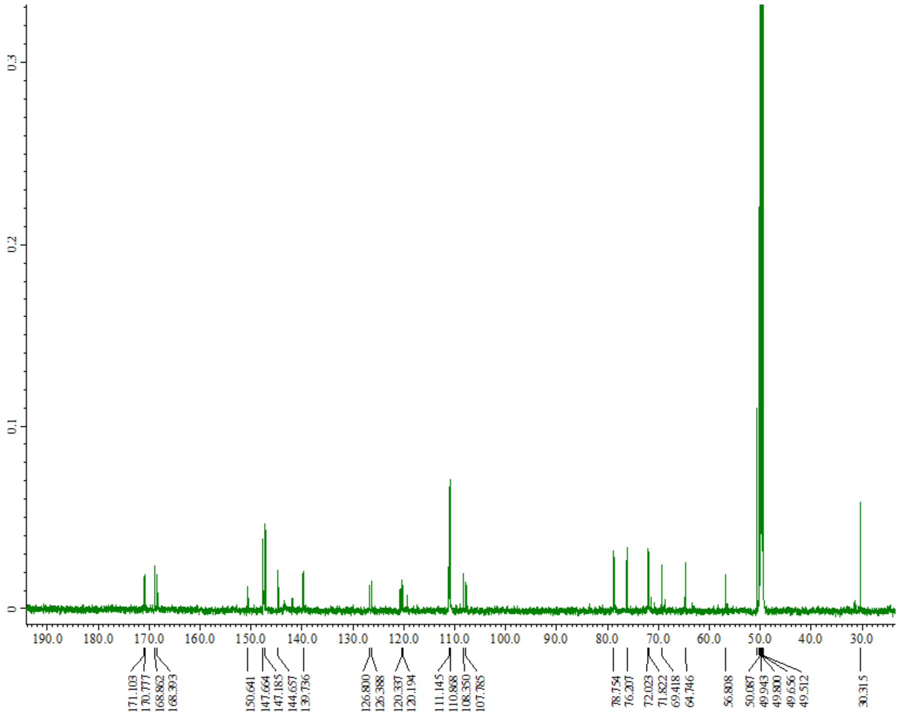

Supplement: Supplementary file 1 [file molecules-24-04340-s001.pdf]
